# Anti-Cancer Potential of a new Derivative of Caffeic Acid Phenethyl Ester targeting the Centrosome

**DOI:** 10.1016/j.redox.2025.103582

**Published:** 2025-03-05

**Authors:** Catello Giordano, Jonatan Kendler, Maximilian Sexl, Sebastian Kollman, Maxim Varenicja, Boglárka Szabó, Gerald Timelthaler, Dominik Kirchhofer, Oldamur Hollóczki, Suzanne D. Turner, Richard Moriggl, Lukas Kenner, Mohamed Touaibia, Olaf Merkel

**Affiliations:** a Department of Pathology, Medical University of Vienna, Vienna, Austria; b Department of Biological Sciences and Pathobiology, Pharmacology and Toxicology, University of Veterinary Medicine Vienna, Vienna, Austria; c Department of Physical Chemistry, University of Debrecen, Debrecen, Hungary; d Center for Cancer Research, Medical University of Vienna, Vienna, Austria; e European Research Initiative on ALK-Related Malignancies (ERIA), Cambridge, UK; f Division of Cellular and Molecular Pathology, Department of Pathology, University of Cambridge, Addenbrooke's Hospital, Cambridge, UK; g Faculty of Medicine, Masaryk University, Brno, Czech Republic; h Department of Biosciences and Medical Biology, Paris Lodron University of Salzburg, Salzburg, Austria; i Christian Doppler Laboratory (CDL) for Applied Metabolomics, Medical University of Vienna, Vienna, Austria; j Unit of Laboratory Animal Pathology, University of Veterinary Medicine, Vienna, Austria; k Center for Biomarker Research in Medicine (CBMed) Core Lab 2, Medical University of Vienna, Vienna, Austria; l Department of Molecular Biology, Umeå University, Umeå, Sweden; m Chemistry and Biochemistry Department, Université de Moncton, Moncton, New Brunswick, Canada

## Abstract

Anaplastic Large Cell Lymphoma (ALCL) is an aggressive T-cell lymphoma affecting children and young adults. About 30% of patients develop therapy resistance therefore new precision medicine drugs are highly warranted. Multiple rounds of structure-activity optimization of Caffeic Acid Phenethyl Ester have resulted in CM14. CM14 causes upregulation of genes involved in oxidative stress response and downregulation of DNA replication genes leading to G2/M arrest and subsequent apoptosis induction. In accordance with this, an unbiased proteomics approach, confocal microscopy and molecular modeling showed that TUBGCP2, member of the centrosomal γ-TuRC complex, is a direct interaction partner of CM14. CM14 overcomes ALK inhibitor resistance in ALCL and is also active in T-cell Acute Lymphoblastic Leukemia and Acute Myeloid Leukemia. Interestingly, CM14 also induced cell death in docetaxel-resistant prostate cancer cells thus suggesting an unexpected role in solid cancers. Thus, we synthesized and thoroughly characterized a novel TUBGCP2 targeting drug that is active in ALCL but has also potential for other malignancies.

## Graphical abstract

**Figure:**
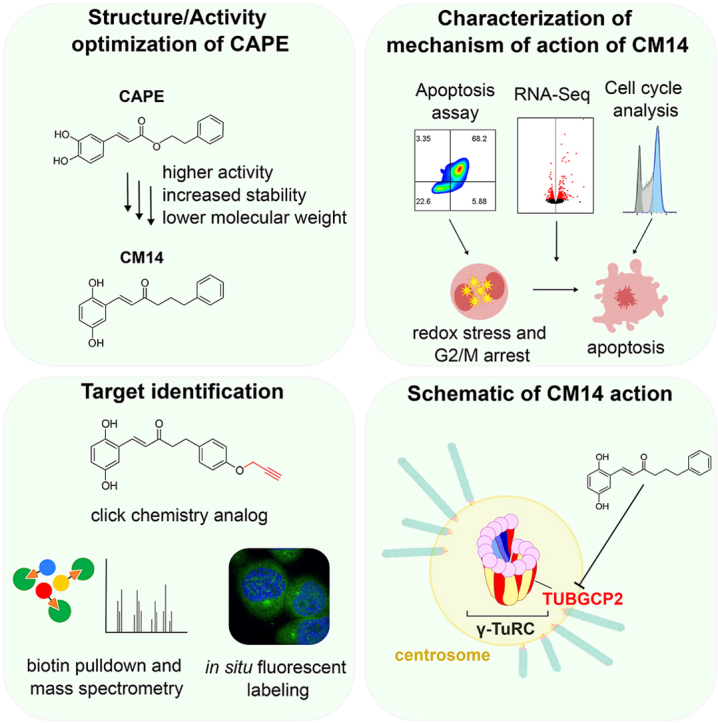

## 1 Introduction

Anaplastic Large Cell Lymphoma (ALCL) is an aggressive CD30^+^ peripheral T-cell lymphoma. The typical fusion gene product Nucleophosmin 1 (NPM)- Anaplastic Lymphoma Kinase (ALK), a constitutively active tyrosine kinase, is present in about half of the cases (ALK+) [1]. Patients without this translocation (ALK-) can be divided into systemic, cutaneous, and recently described breast-implant associated ALCL. Key transcription factors for ALCL pathogenesis are Signal Transductor and Activator of Transcription (STAT3 [2], STAT5 [3]) and the members of the Activator Protein (AP)-1 family (JunB, BATF3 [4], IRF4 [5]). In addition to NPM-ALK [6] they can be activated by tyrosine kinases like TYK2 [7] and Platelet-Derived Growth Factor Receptor β (PDGFRβ) [3]. The ALCL99 trial highlighted that while the standard CHOP regimen (cyclophosphamide, doxorubicin, vincristine, prednisone) is effective in ALK+ ALCL [8] the prognosis for systemic ALK- ALCL remains poor, with a five-year survival rate of only 30–50% [9]. The recent addition of targeted therapies like the anti-CD30 antibody-drug conjugate Brentuximab Vedotin (BV) or the ALK inhibitor Crizotinib has improved response rates but comes with significant side effects like polyneuropathy [10] or high thrombosis rates, respectively [11]. ALK-specific inhibitors alone are used mainly as second line treatment for relapsing ALK+ patients, however resistance development is a major problem [12]. Also, relapsing/resistant ALK- ALCL patients have no effective approved second line treatments, creating a clinical need for new treatment options [9,13].

Bee glue, also called propolis, is a resin-like substance used by honeybees to seal, disinfect and strengthen the structure of the hive [14]. One of its main active components is Caffeic Acid Phenethyl Ester (CAPE), a cinnamic acid ester bearing two hydroxyls at positions 3 and 4 of the phenyl ring and a phenethyl moiety [15]. CAPE has been shown to have anti-inflammatory [16], immunomodulatory [17] and antioxidant [18,19] properties and acts as a potent inhibitor of the pro-inflammatory NF-κB pathway [20]. This pathway has been shown to be driven by the ALCL surface marker CD30 [21]. Additionally, CAPE can exert anti-proliferative and pro-apoptotic activity on cancer cells by interfering with pro-oncogenic pathways [22] and inducing oxidative stress [23].

In this study we have used CAPE as a lead molecule to generate more active derivatives that are able to induce apoptosis in ALCL cells. Following chemical modification and anti-proliferative screenings to examine structure-activity relationships, we identified a novel, more active ketone analog, CM14. We further characterized its mechanism of action using RNA-Seq, flow cytometry, Western blot, cell cycle analysis and *in silico* molecular modelling. A click chemistry-amenable derivative of CM14 allowed us to perform fluorescence imaging and pull-down of drug-interacting proteins. Our data show that CM14 holds potential therapeutic value for lymphoma but also other types of cancer.

## 2 Results

### 2.1 Design of CM14, a new derivative of CAPE

**Fig. 1:**
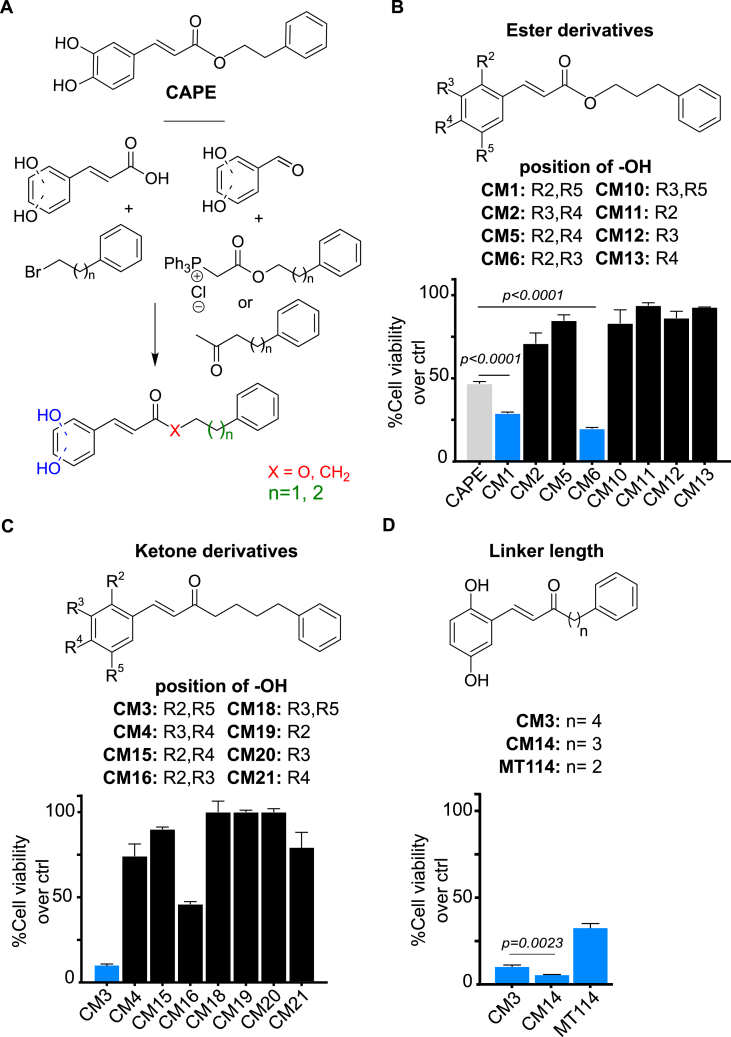
**Structure-activity optimization and identification of CAPE derivative CM14.** (A) Chemical structure of CAPE and synthetic strategy used for derivatives generation. (B,C,D) The ALCL cell line Mac-2a was incubated with 5 μM of respective compound for 72 h. Position of OH groups (R) and number of methylenes (n) are indicated. Non-specified R groups are H atoms. Relative viability/metabolic activity compared to untreated control was measured via resazurin assay in 3 replicates (mean ± SD). Unpaired two-sided Student's *t*-test was performed for statistical evaluation. Compounds in the bar diagram which showed higher activity than CAPE are colored blue.

Inspired by CAPE, new derivatives with therapeutic potential for ALCL were designed, synthesized and tested in the present study (see Supplementary Information for full synthesis and characterization details of all synthesized new compounds). Ester analogs were synthesized either by the one-step esterification of hydroxycinnamic acids optimized in our lab or via the Witting coupling using the appropriate aldehyde and stabilized phosphonium ylide [15,[24], [25], [26], [27]] (Fig. 1A). We then proceeded to test the effect of the newly synthesized compounds on ALCL cells viability. To investigate the effect of the position of the hydroxyls, we replaced the original 3,4-dihydroxyl substitution of CAPE with the 2,5-, 2,4-, 2,3-, and 3,5-dihydroxyl substitution. (Fig. 1B). The change of the positions of the hydroxyls to positions 2 and 5 of the phenyl ring (CM1) as well as to positions 2 and 3 (CM6) resulted in enhanced viability reduction as compared to CAPE. In contrast, the 3,5-dihydroxyl and 2,4-dihydroxyl substitutions, as in compounds CM5 and CM10, resulted in complete loss of activity (Fig. 1B). We also reduced the number of hydroxyls to one by having it in positions 2-, 3- or 4- of the phenyl ring. Derivatives with a single hydroxyl, whether at position 2 (CM11), at position 3 (CM12), or at position 4 (CM13) had no effect on viability, demonstrating the necessity of both hydroxyls for activity. Esters CM1 and CM6 were the most active molecules in reducing ALCL cell viability (Fig. 1B). Hitherto the compounds used were all esters, which can be easily hydrolyzed by endogenous hydrolases. To achieve higher *in vivo* stability, ketone analogs of the ester subseries were synthesized through an aldol condensation with the appropriate benzaldehyde and ketone [25,27] (Fig. 1C). Ketones CM4, CM15, CM18, CM19, CM20 and CM21 of the second subseries showed low or no activity. In contrast, CM3, was substantially more active than its ester analog CM1. CM16 performed worse than its ester analog CM6 (Fig. 1C). These results confirm that the 2,5-hydroxyl substitution is the optimal arrangement for a ketone derivative. As a final optimization step, we investigated the optimal linker length between the carbonyl and unsubstituted phenyl ring (Fig. 1D). CM14 (3 methylenes, n = 3) was more effective than MT114 (2 methylenes, n = 2) or CM3 (4 methylenes, n = 4) suggesting an optimal linker length of 3 methylenes (Fig. 1D).

### 2.2 CM14 causes apoptotic cell death and overcomes drug resistance

**Fig. 2:**
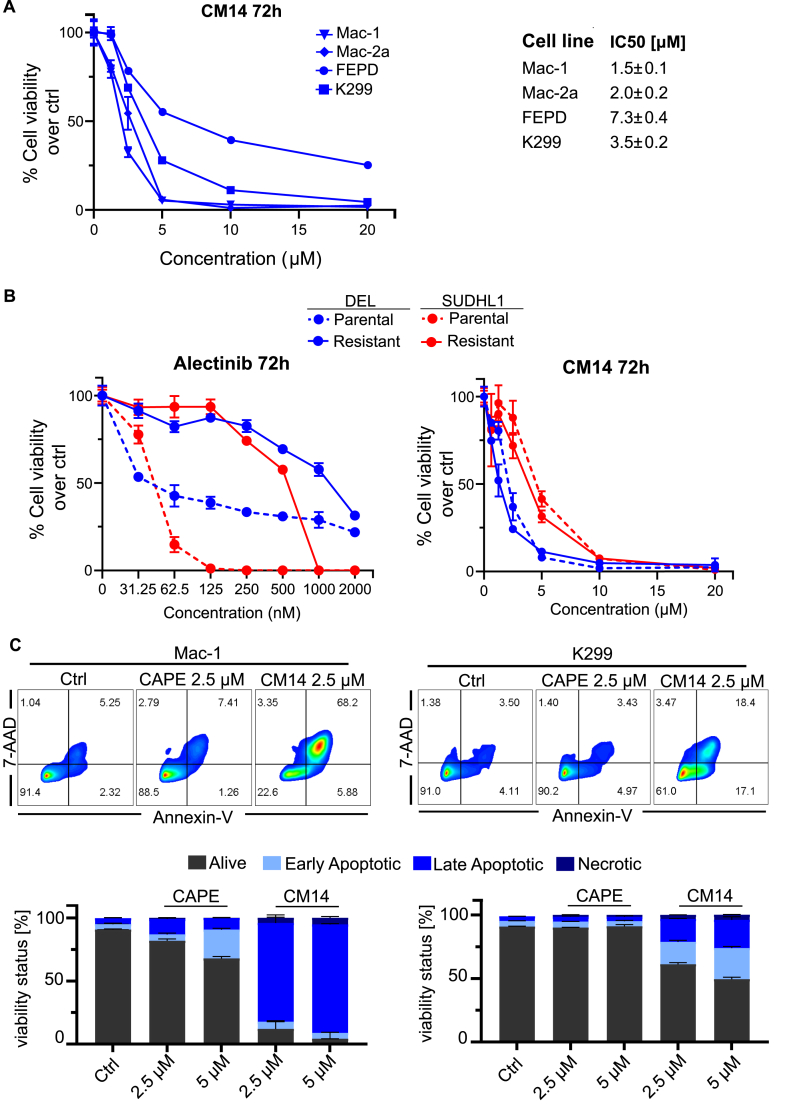
**CM14 induces apoptosis of ALCL cells and overcomes drug resistance.** (A) Dose-response curves of ALCL cell lines treated with CM14 for 72 h. Relative viability compared to control was measured via resazurin assay in 3 replicates. 72 h IC50 values for CM14 are shown in μM ± SD. (B) The parental ALK+ ALCL cell lines DEL and SUDHL1 and the corresponding alectinib-resistant cell lines were treated with indicated concentrations of alectinib and CM14 for 72 h. Viability was measured via resazurin assay. (C) Mac-1 and K299 ALCL cell lines were stained with Alexa Fluor-488 Annexin-V and 7-AAD after treatment with CM14 and CAPE for 24 h at indicated concentrations. Density plots show one representative replicate and bar graphs show means ± SD of biological triplicates.

CM14, being now our top candidate, was then tested in a panel of ALK- (Mac-1, Mac-2a, FEPD) and ALK+ (K299) ALCL cell lines. CM14 was able to reduce ALCL viability in all cell lines tested with an IC50 between 1.5 and 7.3 μM (Fig. 2A). Development of drug resistance is one of the greatest challenges of cancer therapy. This also applies to ALK inhibitors used in the treatment of lung cancer and ALCL [28]. We generated two ALK+ ALCL cell lines resistant to the 2nd generation ALK inhibitor alectinib by long-term incubation with increasing concentrations of the drug. As shown in Fig. 2B, both parental cell lines are sensitive to alectinib with an IC50 below 100 nM, while the resistant cell lines had an IC50 of 429 nM and 1081 nM, respectively. However, when treated with CM14, the alectinib-resistant SUDHL1 and DEL cells were equally sensitive as the parental cell lines suggesting that CM14 can effectively overcome ALK inhibitor resistance (Fig. 2B). Next, Annexin-V and 7-AAD staining was performed after CM14 treatment and analyzed by flow cytometry. A marked induction of apoptosis as shown by positivity for Annexin-V and 7-AAD following 24 h exposure to 2.5 and 5 μM CM14 was seen in all 4 ALCL cell lines tested, whereas only minor effects were observed for CAPE (Fig. 2C–SFig. 1A). Similarly, PARP cleavage was also observed in CM14-treated cells (Mac-1, K299, FEPD; SFig. 1B). In contrast, CAPE-treated cell lines showed no (K299, FEPD) or minor (Mac-1) PARP cleavage. Peripheral blood mononuclear cells from healthy donors were almost not affected when treated with 2.5 and 5 μM CM14 for 24 h (SFig. 1C).

### 2.3 CM14 represses DNA replication genes and induces G2/M arrest

**Fig. 3:**
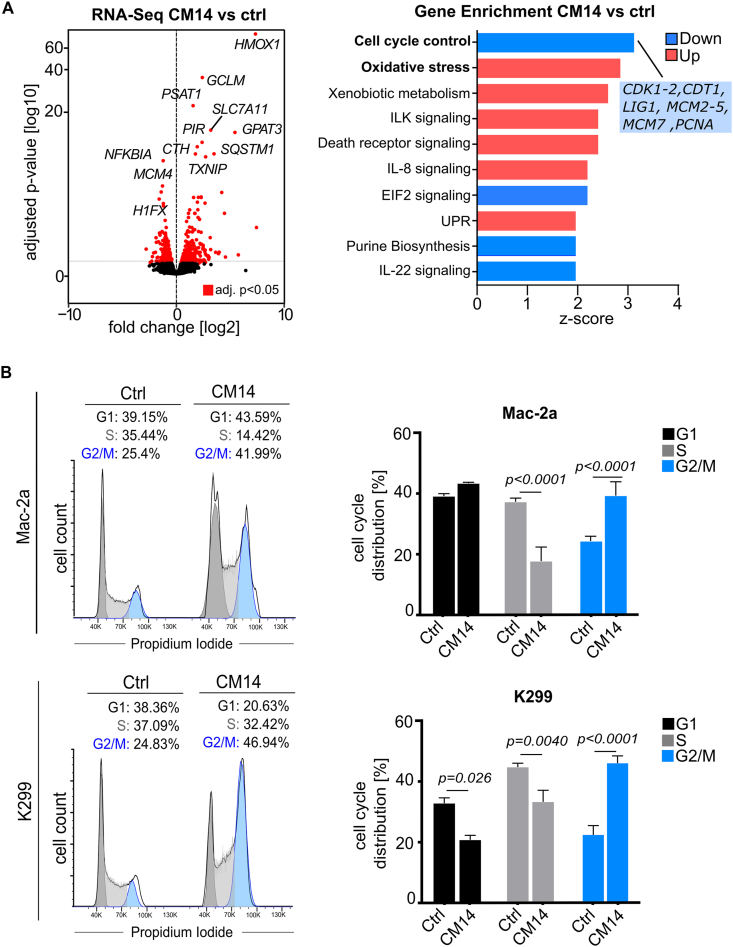
**RNA-Seq and propidium iodide staining reveal oxidative stress and G2/M arrest induced by CM14.** (A) RNA-Seq of Mac-2a cells treated for 12 h with CM14 or DMSO: volcano plot and Ingenuity Pathway Analysis of differentially expressed genes with adj. p-value <0.05. The top 10 significant pathways (p-value <0.05) with a z-score ≥1 (red) and ≤-1 (blue) are shown: “Cell Cycle Control of Chromosomal Replication”; “NRF2-mediated Oxidative Stress Response”; “Xenobiotic Metabolism General Signaling Pathway”; “ILK Signaling”; “Death Receptor Signaling”; “IL-8 Signaling”; “EIF2 Signaling”; “Unfolded protein response”; “Purine Nucleotides De Novo Biosynthesis II”; “IL-22 Signaling”. (B) ALCL cell lines were treated with CM14 (Mac-2a 1.25 μM, K299 2.5 μM) for 12 h. DNA content was measured via intracellular propidium iodide staining. Histograms from representative replicates and bar graphs with mean ± SD of percentage of cells in G1, S and G2/M phase are shown. Unpaired *t*-test was used for statistical analysis.

To decipher the mechanism of cell death induction in more depth, we performed RNA-Seq 3 h, 6 h and 12 h after treatment of the ALCL cells with CM14 (see Supplementary Information for full method details). In accordance with previous observations for CAPE [23], we found strong deregulation of oxidative stress response genes at all three time points (Fig. 3A, Supplementary Table 1). CM14 is an α-β unsaturated ketone, a class of molecules which are known to be readily attacked by cellular nucleophilic moieties like thiol groups found in cysteine residues of proteins or glutathione [29]. Therefore, it is not surprising that CM14 induces activation of oxidative stress response. Indeed, cellular supplementation of thiol groups with N-Acetylcysteine could rescue CM14-induced cell viability reduction, however only partially (SFig. 1D), suggesting that other mechanisms apart from redox unbalance contribute to CM14 activity. Indeed, gene set enrichment at the 12 h time point revealed cell cycle control genes to be enriched and downregulated (*e.g. CDK1, CDK2, CDT1, LIG1, MCM2, MDM3, MCM4, MCM5, MCM7, PCNA*; Fig. 3A). These results prompted us to analyze cell cycle progression in CM14-treated ALCL cells. Following 12 h of CM14 exposure, ALCL cells treated with CM14 showed a marked accumulation of cells at the G2/M phase and reduction of cells in the S-phase as shown by intracellular PI staining in all ALCL cell lines analyzed (Fig. 3B–SFig. 1E). This is in accordance with downregulation of cell cycle-associated genes described above and suggests mitotic stress leading to apoptosis. What we observed is reminiscent of a phenomenon termed “mitotic catastrophe” which promotes apoptosis in response to misguided chromosome separation or DNA damage [30].

### 2.4 Generation of alkyne derivative for drug localization studies

**Fig. 4:**
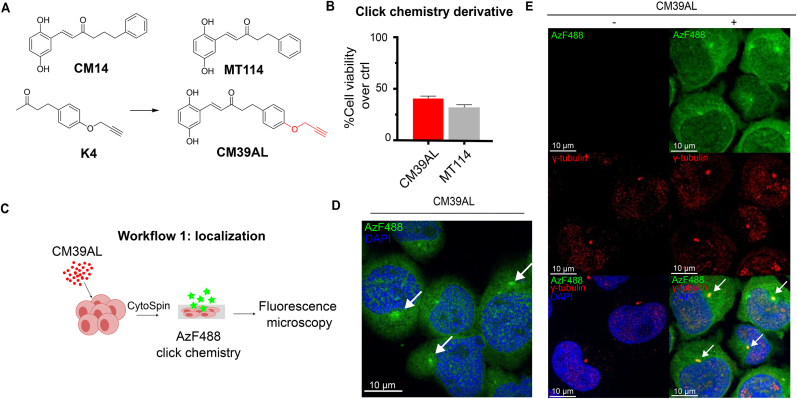
**Testing of a click chemistry derivative of CM14.** (A) Structures of starting ketone K4 and alkyne derivative CM39AL are shown together with the analogs CM14 and MT114. (B) Effect of CM39AL and closest analog MT114 on the viability of Mac-2a cells after 72 h incubation at 5 μM. Relative viability to DMSO control was measured via resazurin assay. Data are means ± SD of biological triplicates. (C–D) Mac-2a cells were treated with CM39AL or DMSO and incubated with AzF488 in a CuAAC click chemistry reaction mix. DAPI was used to stain nuclei. Photos were acquired using spinning disk confocal microscopy. White arrows indicate cytoplasmic spots where green fluorescence signal accumulates. (E) After click chemistry, cells were stained with anti-γ-tubulin antibody and immunofluorescence imaging was performed using spinning disk confocal microscopy. White arrows indicate overlapping green and red signals.

After we have shown that CM14 leads to G2/M arrest and apoptotic cell death, we wanted to investigate what causes these effects. Copper(I)-catalyzed azide-alkyne cycloaddition (CuAAC) also called “click chemistry” is a versatile tool to link marker molecules to drug candidates [31]***.*** In brief, an azide reacts with an alkyne side group forming a triazole ring that covalently binds the drug candidate to the desired probe. The absence of a ketone with three methylenes and a hydroxyl moiety, necessary to attach the azide or alkyne in CM14 compelled us to use the close CM14 analog MT114 for a simple and efficient synthesis strategy. The terminal alkyne moiety was attached to the unsubstituted phenyl ring by a covalent ether bond generating a new derivative, CM39AL (Fig. 4A). The analog CM39AL has two methylenes in the linker like MT114 but preserves the position and number of hydroxyl groups of CM14 on the substituted phenyl ring, which we found are the main determinants of the activity of CM14. This makes it highly likely that CM39AL preserves the mechanism of action and molecular targets of CM14. Next, we tested CM39AL for its potential to reduce viability of ALCL cells. The addition of a terminal alkyne (CM39AL, red) retained the activity of the parental compound MT114 (grey), suggesting it as useful probe for further experiments (Fig. 4B). We proceeded to investigate the subcellular localization of CM39AL. ALCL cells were treated for 2 h with CM39AL (40 μM), fixated on a glass slide and intracellular CM39AL was covalently bound to Azide Flour-488 (AzF488) using “click chemistry” (Fig. 4C). In the control only the solvent DMSO was used and cells underwent the same click chemistry reaction. Using spinning disk confocal microscopy [32] we observed a clear fluorescent signal in the CM39AL-treated cells but none in the DMSO-treated samples (Fig. 4D–SFig. 3A). The fluorescence signal was found mostly in the cytosol but also in the nucleus. However, we observed in each cell one region of high fluorophore intensity in direct vicinity of the nucleus. This reminded us of centrosomes in the interphase. The centrosome is a crucial component of cell division machinery since it orchestrates mitotic spindle assembly and chromosome segregation [33]. Therefore, we looked for actively dividing cells, and indeed we found that the fluorophore accumulated in two spots in the center of the condensed chromosomes (SFig. 2A). In contrast to α- and β-tubulin, which form the microtubular structures of the mitotic spindle, γ-tubulin is involved in microtubule nucleation and is therefore a good and widely used centrosome marker [34]. Incubation with an antibody for γ-tubulin (red) showed colocalization (yellow, see arrows) with the CM39AL-AzF488 conjugate (green, Fig. 4E).

### 2.5 Identification of protein interaction partners and molecular modelling of interaction site

**Fig. 5:**
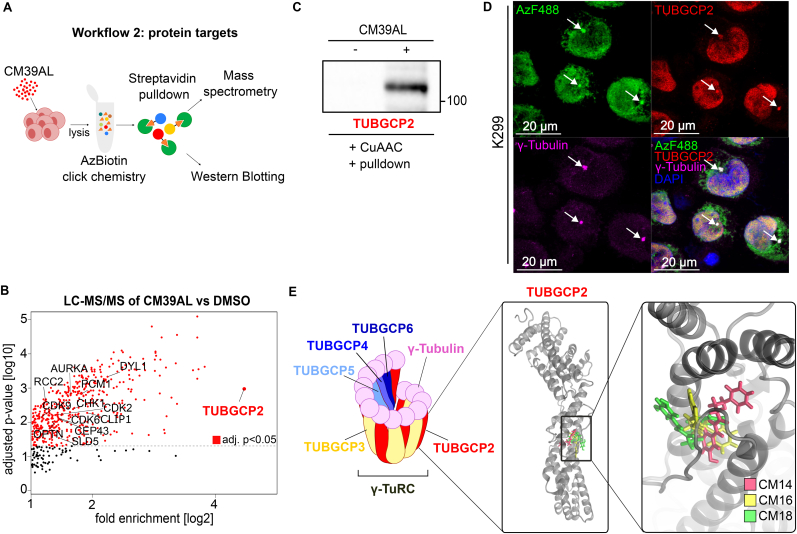
**Proteomics identifies TUBGCP2 as a target of CM39AL.** (A) Schematic of experimental strategy: Mac-2a cells were incubated with CM39AL or DMSO. CuAAC with AzBiotin was performed in the cell extracts with (+) or without (−) CM39AL. Bound proteins were enriched using streptavidin-agarose resin and analyzed via Western blotting or LC-MS/MS. (B) LC-MS/MS analysis results with log2 fold enrichment versus adjusted p-value of proteins identified in all four biological replicates of both conditions. Proteins involved in regulation of cell cycle, mitosis and mitotic spindle are highlighted, including the strongly enriched TUBGCP2 protein. (C) After performing again CuAAC with AzBiotin and streptavidin enrichment, pulled down proteins were analyzed by immunoblotting with anti-TUBGCP2 antibody. (D) TUBGCP2 as CM39AL target via immunofluorescence imaging: after on slide *in situ* CuAAC labeling of CM39AL with AzF488, cells were stained with anti-γ-tubulin and anti-TUBGCP2 antibodies. Photos were acquired via spinning disk confocal microscopy. White arrows indicate overlapping signals from CM39AL (green), TUBGCP2 (red) and γ-tubulin (magenta). (E) Schematic representation of the γ-TuRC and overlaid orientations of CM14 (red), CM16 (yellow), and CM18 (green) within TUBGCP2, obtained from molecular docking.

Streptavidin-biotin -down is a strategy often used to identify drug-interacting proteins [35]. Incubation of ALCL cells with or without CM39AL was followed by cell lysis and CuAAC to couple Azide-Biotin (AzBiotin) to CM39AL (Fig. 5A). The resulting reaction mix was separated on SDS-PAGE, blotted and probed with streptavidin. Several bands of biotinylated proteins were observed in CM39AL-treated cells but not in untreated cells (SFig. 2B), confirming the presence of interacting proteins. Therefore, the remaining reaction mix was incubated with streptavidin-conjugated agarose resin. After extensive washing, enriched proteins were digested on-bead and peptides were analyzed via Liquid Chromatography-tandem Mass Spectrometry (LC-MS/MS). We compared proteins enriched in CM39AL-treated cells in all 4 biological replicates as compared to DMSO treated cells (Fig. 5B–Supplementary Table 1). Enriched proteins were found to be involved in mitotic spindle and centrosome assembly (CEP43, OPTN, CLIP1, RCC2, PCM1, TUBGCP2) but also in regulation of cell cycle progression (CDK2, CDK6, CDK9, CHK1, Fig. 5B–SFig. 2C). By far, the strongest interaction was seen with a protein called TUBGCP2, which was enriched more than 20-fold. TUBGCP2 is a major component of the γ-Tubulin Ring Complex (γ-TuRC), the scaffold structure required for α- and β-tubulin nucleation at the centrosome [33]. To validate this finding, we repeated the streptavidin pull-down, and enriched proteins were immunoblotted and then probed with an antibody targeting TUBGCP2, revealing a band in the CM39AL-treated lysate but not in the control (Fig. 5D). This reinforced our assumption that the centrosome is the site of CM14 action. Moreover it identified the preferred molecular interaction partner of CM14. To see whether CM39AL and the TUBGCP2 indeed colocalize in intact cells at the centrosome, we performed confocal microscopy of CM39AL-ALCL cells after co-staining with AzF488, anti-TUBGCP2 and anti-γ-tubulin antibodies. As expected, the CM39AL and the TUBGCP2 signals were found to accumulate at the centrosome, as demonstrated by white cytoplasmic spots (see white arrows, Fig. 5E–SFig. 3A). Partial localization of TUBGCP2 in the nucleus was described before[36,37], and we also found CM39AL in the nucleus with a similar distribution, corroborating the idea of a direct CM39AL/TUBGCP2 interaction. To our knowledge no other substances have been described to date that target the TUBGCP2 protein, thus revealing a novel γ-TuRC interacting molecule. Finally, to gain a molecular level insight into how CM14 interacts with TUBGCP2, we performed a combined molecular dynamics and docking study using a publicly available TUBGCP2 structure (see Supplementary Information for full method details). In this analysis we also included CM16 as a molecule with intermediate activity and CM18 as non-active ketone analog. The structures obtained from this procedure revealed an identical binding site on TUBGCP2 for all 3 tested molecules (Fig. 5E): while CM18 and CM16 showed interaction with the aqueous environment at the level of the substituted phenyl ring, CM14, the most active substance, was fully surrounded by aminoacidic residues, with no interaction with the solvent. Notably, the identified binding site is adjacent to the domain that is responsible for the association TUBGCP2 with TUBGCP3 (SFig. 3B), which is the first step in γ-TuRC assembly.

### 2.6 CM14 as interactor of the γ-Tubulin Ring Complex and possibly broadly active anticancer molecule

**Fig. 6:**
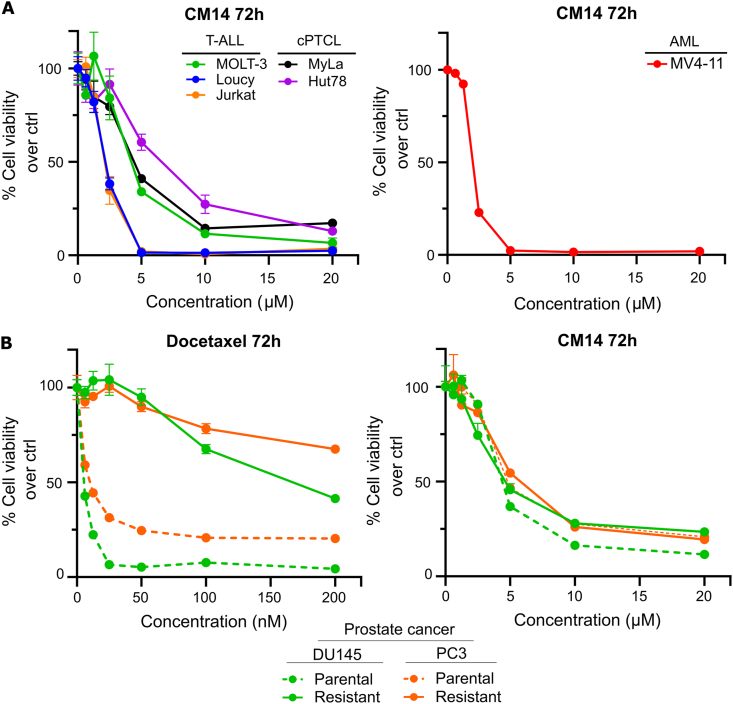
**CM14 is effective in other lymphomas/leukemias and docetaxel-resistant prostate cancer.** (A) Dose-response curves of T-ALL, cPTCL and AML cell lines treated with CM14 for 72 h. Relative viability compared to control was measured via resazurin assay in 3 replicates and it is shown as mean ± SD. (B) Dose-response curves of parental and docetaxel-resistant prostate cancer cell lines DU145 and PC3 treated with docetaxel and CM14 for 72 h. Relative viability compared to control was measured via resazurin assay in 3 replicates and it is shown as mean ± SD.

In ALK+ ALCL aberrant centrosomes with enhanced size or increased number have been described [38]. Amplified centrosomes have been identified in other hematological malignancies [39,40] as well as in many solid tumors such as breast, prostate, colon, ovarian and pancreatic cancer [[41], [42], [43], [44]]. Centrosome amplification has also been implicated in contributing to poor clinical prognosis [39,43,45]. Therefore, targeting the centrosome may be of interest for lymphoma and leukemia treatment but also in solid cancers. This prompted us to test CM14 in other lymphoma and leukemia types including cutaneous Peripheral T-cell Lymphoma (cPTCL), T-cell Acute Lymphoblastic Leukemia (T-ALL) and Acute Myeloid Leukemia (AML). IC50 values were comparable to those seen in ALCL cells, with the highest activity in the T-ALL cell lines Loucy and Jurkat and AML cell line MV4-11 (Fig. 6A). Furthermore, we wanted to test a representative example of solid cancer. We have chosen two p53 non-functional late-stage prostate cancer cell lines with increased centrosome number and size (DU145 and PC3) [46]. In addition, we used matched docetaxel-resistant cell lines. Interestingly, docetaxel-resistant and parental cell lines had similar IC50 values when treated with CM14 (Fig. 6B) thus highlighting the possibility to overcome taxanes resistance with CM14 in these highly pretreated patients with otherwise very limited treatment options.

## 3 Discussion

In this study, we designed and synthesized 18 derivatives of CAPE with the objective of identifying novel compounds with enhanced therapeutic efficacy for the treatment of ALCL. Among these, the lead compound CM14 demonstrated a significant increase in potency. Several lines of evidence point to the centrosome as the site of CM14 action including downregulation of cell cycle associated genes, cell cycle arrest in the G2/M phase, localization at a region close to the nucleus and co-staining with the centrosome marker γ-tubulin. Finally, streptavidin pull-down revealed the centrosomal protein TUBGCP2 as the main target of CM14. *In silico* molecular dynamics and docking studies identified a likely binding site on TUBGCP2. γ-TuRC assembly is initiated by the recruitment of TUBGCP2 to TUBGCP3, each binding one γ-tubulin, to form the so-called γ-Tubulin Small Complex (γ-TuSC) [47]. In a second step, TUBGCP4-6 assemble with γ-TuSCs to form a mature 14-spoked γ-TuRC. Interestingly, the CM14 binding site is close to the TUBGCP2/TUBGCP3 interface. This finding implies that CM14 might interfere with the first step in the formation of γ-TuSC, and thus the assembly of the full γ-TuRC [47,48]. In addition to cell cycle and replication-associated genes, after CM14 challenge we also found upregulation of genes responding to oxidative stress, also referred to as vitagenes [49], mediated by the master regulator NRF2. In line with this, N-Acetylcysteine was able to block the effect of CM14 at least partially, indicating CM14's strong effect on the cells redox balance. Oxidative and nitrosative stress is particularly detrimental for neurons and involved in neurodegenerative diseases [[49], [50], [51]]. Of note, neurotoxicity as a consequence of chemotherapy-induced oxidative stress has been described [[52], [53], [54]]. In contrast, low levels of NRF2-activating redox stress could prevent damage to neural cells whereas higher levels are toxic, a phenomenon often observed in diverse contexts and referred to as hormesis [55,56]. Therefore, it will be important in the future to perform in-depth studies of the dose/response of CM14 on cancer and neural cells. However, an important component of CM14's mechanism of action is very likely the impact on the cell cycle caused by interference with microtubule nucleation. Therapeutics like taxanes and vinca alkaloids that target tubulin dynamics and mitotic spindle formation are among the most successful and widely used cancer drugs, collectively defined as Microtubules Targeting Agents (MTAs). Taxanes have turned out to be more successful in solid cancers including breast, lung, bladder and prostate cancer whereas vinca alkaloids are primarily utilized in hematological malignancies [57]. Oncovin, the brand name for vincristine, is a component of the CHOP regimen used in the ALCL99 trial, which reported a 10-year survival rate of 90% in pediatric patients [8]. However, in the Echelon II trial Oncovin was successfully replaced with Brentuximab-Vedotin, an anti-CD30 antibody linked to the drug monomethyl auristatin A, which also targets microtubule dynamics [10,58]. Interestingly, vinblastine, another drug of the same family of vincristine, has been shown to be effective as monotherapy in relapsed ALK+ patients [59], underlining the therapeutic relevance of targeting microtubule dynamics with a single agent. The protein with the strongest interaction to our new drug candidate was TUBGCP2 which was identified before in a genetic synthetic lethality screen as a sensitivity gene for paclitaxel in lung cancer. It is interesting to note that other components of the γ-TuRC complex were also among the high confidence hits in this screen. [60]. It has been shown that many drugs affecting mitosis through tubulin interaction or altering the number centrosomes lead to activation of the p53 pathway to induce cell death [61]. However, the fact that our substance is active also in the p53-deficient prostate cancer cell lines DU145 and PC3 suggests the involvement of a p53-independent apoptotic pathway. In addition, the docetaxel-resistant versions developed from these cell lines showed identical IC50 values suggesting also independence from resistance mechanism developed after docetaxel treatment. Moreover, it has been shown that TUBGCP2 and TUBGCP3 are overexpressed in glioma [37]. Therefore, it will be of interest to test our substance in the future also in glioma model systems and see whether enhanced expression translates into enhanced susceptibility.

## 4 Materials and methods

### 4.1 Cell lines

If not specified otherwise, cell lines were purchased from the Deutsche Sammlung für Mikroorganismen und Zellkulturen (DSMZ, Braunschweig, Germany). The cutaneous ALCL cell lines Mac-1 and Mac-2a were gratefully obtained from Marshall Kadin (Boston, USA) and the ALK- systemic ALCL cell line FEPD from Annarosa del Mistro, Padua, Italy. ALCL cell lines were cultivated in RPMI 1640 supplemented with 10% FBS and 100 IU/ml penicillin, 50 mg/ml streptomycin sulfate. Alectinib-resistant cell lines were generated by cultivating cells with increasing Alectinib (Sellekchem) concentrations. 3 × 10^6^ cells per cell line were seeded into 6-well plates with a drug concentration of the respective cell line's IC50 value. Medium was exchanged with fresh drug-supplemented medium every 3–4 days. When cells reached confluence, as observed by medium color change, within 3–4 days, we increased the drug concentration by 10 nM. After 4 months of cultivation, we set the respective drug concentration as final and validated it by comparing the proliferation of not resistant and resistant cell lines. Resistant cell lines were cultured and maintained at a final concentration of 160 nM and 200 nM Alectinib, respectively.

DU145 and PC3 parental and docetaxel-resistant cell lines were kindly provided by Zoran Culig and generated as described previously [62].

PBMCs were isolated from peripheral blood of two healthy donors using Lymphoprep (StemCell) according to manufacturer instructions.

### 4.2 Western blotting

Cells were lysed in RIPA buffer containing phosphatase and protease inhibitors. After Bradford Assay (Sigma Aldrich) quantification, equivalent amounts of protein were diluted in sample buffer and separated by 10% SDS-PAGE. Proteins were transferred to nitrocellulose membranes (Millipore), subjected to immunoblot analysis and incubated in 5% BSA in TBS-Tween with antibodies as listed: anti-PARP (Cell Signaling Technology Cat# 9532, RRID:AB_659884), anti-GAPDH (Cell Signaling Technology Cat# 97166, RRID:AB_2756824), Streptavidin-HRP (Cell Signaling Technology Cat# 3999, RRID:AB_10830897), anti-TUBGCP2 (#PA5-58151, RRID:AB_2641922). Imaging was performed on Chemidoc (Biorad) using ECL Prime Western Blotting Detection Reagent (Cytiva).

### 4.3 Viability assay

All tests were performed in three replicates. 10^4^ cells were seeded in 100 μL of complete RPMI medium and treatments were added in additional 100 μl at indicated concentrations. For N-Acetylcysteine cotreatment, NAC (Sigma Aldrich) was added in cell culture medium at 1 mM for 30 min before cotreatment with CM14 for 72 h. Resazurin solution (0.15 mg/ml in PBS, ChemiCruz, Dallas, TX) was added to cell culture medium (1:5 vol/vol). After 3 h at 37C° fluorescence emission was measured via the Synergy H1 microplate reader (BioTek) using the following wavelengths: Ex = 530–570 nm, Em = 590–620 nm.

### 4.4 Cu-based alkyne-azide “click” cycloaddition and streptavidin pull-down

Cells were pelleted, washed twice with PBS and lysed via sonication using Bioruptor (Diagenode, Seraing, Belgium) with cOmplete EDTA-free Protease inhibitors in PBS (Roche, Basel, Switzerland). Protein lysate was clarified by centrifugation at 13000 rpm for 10 min. CuAAC reaction mix (guanidine HCl 5 mM; Sodium Ascorbate 5 mM; CuSO_4_ 230 μM; Tris(3-hydroxypropyltriazolylmethyl)amine 1.15 mM; PEG-Biotin-Azide 150 μM (all Sigma Aldrich) was added to the protein lysate (1 mg protein/ml) and incubated for 1.5 h on a rotator at RT. Next, acetone precipitation was performed and proteins were resuspended in 800 μL of 0.1% SDS in PBS using sonication. Then, 200 μL of Streptavidin Agarose Resin (ThermoFisher) were washed in PBS, resuspended in 0.1% SDS in PBS, added to the protein solutions and put on a head over tail shaker for 2 h at RT. Beads were washed with PBS containing SDS: 2 × 0.1%, 4 × 1% and 2× PBS alone. Samples were stored at −80 °C until further processing. For immunoblotting, beads were boiled in 1x Laemmli Buffer at 95 °C for 5 min.

### 4.5 On-bead digestion for MS

Protein complexes were digested directly on beads by addition of 0.75 μg (1 μg/μl) of trypsin (sequencing grade, Promega) in 50 mM (NH_4_)HCO_3_ buffer. Beads were gently tapped to ensure even suspension of trypsin solution and incubated at 37 °C with mild agitation for 2 h. The partially digested complex was transferred to the clean tubes to separate it from the beads and incubated at 37 °C for 16 h without agitation. Resulting peptides were extracted into LC-MS vials by 2.5% formic acid (FA) in 50% acetonitrile (ACN) and 100% ACN with addition of polyethylene glycol (20,000; final concentration 0.001%) [63]. Peptides were then cleaned by liquid-liquid extraction (3 iterations) using water saturated ethyl acetate [64] and concentrated in a SpeedVac concentrator (Thermo Fisher Scientific).

### 4.6 LC-MS/MS analysis

LC-MS/MS analyses of all peptides were done using UltiMate 3000 RSLCnano system (Thermo Fisher Scientific) connected to timsTOF Pro spectrometer (Bruker). Prior to LC separation, tryptic digests were online concentrated and desalted using trapping column (μPrecolumn PepMap100C18, dimensions 300 μm ID, 5 mm long, 5 μm particles, Thermo Fisher Scientific). After washing of the trapping column with 0.1 % formic acid (FA), the peptides were eluted (flow rate - 200 nl/min) from the trapping column onto an analytical column (Aurora C18, 75 μm ID, 250 mm long, 1.6 μm particles, Ion Opticks) by 60 min linear gradient program (3–42% of mobile phase B; mobile phase A: 0.1% FA in water; mobile phase B: 0.1% FA in 80% ACN). Equilibration of the trapping column and the analytical column was done prior to sample injection to sample loop. The analytical column was placed inside the Butterfly Heater (Phoenix s&t) and its emitter side was installed inside the CaptiveSpray ion source (Bruker) according to the manufacturer instructions with the column temperature set to 50 °C. Data acquisition, processing and statistical analysis of LC-MS/MS experiment is further described in detail in Supplementary Information.

## CRediT authorship contribution statement

**Catello Giordano:** Writing – review & editing, Writing – original draft, Methodology, Investigation, Data curation, Conceptualization. **Jonatan Kendler:** Writing – review & editing, Methodology, Investigation, Conceptualization. **Maximilian Sexl:** Methodology, Investigation. **Sebastian Kollman:** Methodology, Investigation, Conceptualization. **Maxim Varenicja:** Software, Methodology, Investigation. **Boglárka Szabó:** Software, Methodology, Investigation. **Gerald Timelthaler:** Methodology. **Dominik Kirchhofer:** Methodology. **Oldamur Hollóczki:** Software, Methodology, Investigation. **Suzanne D. Turner:** Writing – review & editing. **Richard Moriggl:** Writing – review & editing. **Lukas Kenner:** Writing – review & editing. **Mohamed Touaibia:** Writing – review & editing, Writing – original draft, Resources, Methodology, Investigation, Funding acquisition, Conceptualization. **Olaf Merkel:** Writing – review & editing, Writing – original draft, Supervision, Resources, Project administration, Investigation, Funding acquisition, Conceptualization.

## Data availability statement

Additional Methods are available in Supplementary Information**.** Full synthesis and characterization details of all synthesized new compounds are included in the Supplementary Information. All datasets generated and analyzed during this study (RNA-Seq, LC-MS/MS, IPA) are included in this published article and in Supplementary Table 1. The LC-MS/MS proteomics raw data have been deposited to the ProteomeXchange Consortium via the PRIDE [65] partner repository with the dataset identifier PXD061079. The LC-MS/MS Processing workflow has been deposited on WorkflowHub registry with the access link https://doi.org/10.48546/workflowhub.workflow.1309.1.

## Declaration of competing interest

A patent has been filed regarding the use of CM14 with interim application number AOF2889/2024. No other competing interests to be declared.
