# Supplementary material for: Anti-Cancer Potential of a new Derivative of Caffeic Acid Phenethyl Ester targeting the Centrosome
Source: Redox Biol. 2025 Mar 5;81:103582. doi: 10.1016/j.redox.2025.103582 (PMC11951030; doi:10.1016/j.redox.2025.103582)
Supplement: Multimedia component 1 [file mmc1.pdf]

Supplementary Information to accompany

Anti-Cancer potential of a new Derivative of Caffeic Acid Phenethyl Ester targeting the centrosome

Catello Giordano[1], Jonatan Kendler[2], Maximilian Sexl[2], Sebastian Kollman[2], Maxim Varenicja[3], Boglárka Szabó[3], Gerald Timelthaler[4], Dominik Kirchhofer[4], Oldamur Hollóczki[3], Suzanne D. Turner,[5,6,7], Richard Moriggl[8], Lukas Kenner[1,5,9,10,11,12], Mohamed Touaibia[13]*, Olaf Merkel[1,5]*

[1]Department of Pathology, Medical University of Vienna, Vienna, Austria

[2]Department of Biological Sciences and Pathobiology, Pharmacology and Toxicology, University of Veterinary Medicine Vienna, Vienna, Austria

[3]Department of Physical Chemistry, University of Debrecen, Debrecen, Hungary

[4]Center for Cancer Research, Medical University of Vienna, Vienna, Austria

[5]European Research Initiative on ALK-Related Malignancies (ERIA), Cambridge, UK

[6]Division of Cellular and Molecular Pathology, Department of Pathology, University of Cambridge, Addenbrooke's Hospital, Cambridge, UK

[7]Faculty of Medicine, Masaryk University, Brno, Czech Republic.

[8]Department of Biosciences and Medical Biology, Paris Lodron University of Salzburg, Salzburg, Austria

[9]Christian Doppler Laboratory (CDL) for Applied Metabolomics, Medical University of Vienna, Vienna, Austria

[10]Unit of Laboratory Animal Pathology, University of Veterinary Medicine, Vienna, Austria

[11]Center for Biomarker Research in Medicine (CBMed) Core Lab 2, Medical University of Vienna, Vienna, Austria

[12]Department of Molecular Biology, Umeå University, Umeå, Sweden

[13]Chemistry and Biochemistry Department, Université de Moncton, Moncton, New Brunswick, Canada

* These authors contributed equally. Corresponding authors. Email:

mohamed.touaibia@umoncton.ca; olaf.merkel@meduniwien.ac.at

Supplementary figure legends

Supplementary Figure 1: (A) Mac-2a and FEPD cells were stained with Alexa Fluor-488 Annexin-V and 7-AAD after treatment with CM14 and CAPE for 24h at indicated concentrations. Density plots show one representative replicate and bar graphs show means ± SD of biological triplicates. (B) Immunoblot of total (t) and cleaved (c) PARP after 24h treatment with CM14 and CAPE (Mac-1 2.5 µM, FEPD and K299 5 µM). (C) PBMCs were stained with Alexa Fluor-488 Annexin-V and 7-AAD after treatment with CM14 and CAPE for 24h at indicated concentrations. Density plots show one representative replicate and bar graphs show means ± SD of biological triplicates. (D) NAC- pretreated (30 min, 1mM) ALCL cells were incubated with CM14 for 72h and viability was measured *via* resazurin assay. (E) ALCL cell line Mac-1 was treated with CM14 1.25 µM for 12h. DNA content was measured via intracellular propidium iodide staining. Bar graphs with mean of three replicates ±SD of percentage of cells in G1, S and G2/M phase are shown. Unpaired t-test was used for statistical analysis.

Supplementary Figure 2: (A) ALK- ALCL cells Mac2A were treated with 40 µM CM39AL for 2h. After cell fixation, CM39AL was labeled with AzF488 using CuAAC reaction mix and nuclei were stained with DAPI. Photos were acquired using spinning disk microscopy. In actively dividing cells CM39AL accumulates at the two centrosomes (white arrows). (B) After CuAAC, SDS-PAGE and blotting, proteins were probed using Streptavidin-HRP to visualize biotinylated proteins. (C) Enriched pathways in CM39AL-treated cells compared to DMSO control after LC-MS/MS were identified using the Qiagen IPA package (version 2023).

Supplementary Figure 3: (A) After on slide *in situ* CuAAC labeling with AzF488, cells were stained with anti-γ-tubulin and anti-TUBGCP2 antibodies. Photos were acquired via spinning disk microscopy. White arrows indicate overlapping signals from CM39AL (green), TUBGCP2 (red) and γ-tubulin (magenta). (B) Location of the binding site of CM14 (red) within the TUBGCP2-TUBGCP3 complex (TUBGCP2: light grey; TUBGCP3: dark grey). The structure was generated by overlaying the CM14-TUBGCP2 complex on the TUBGCP2-TUBGCP3 structure, published under the PDB code 6V6B.

Uncropped Western blots images

Fig. 5D uncropped blot

Immunoblot: TUBGCP2

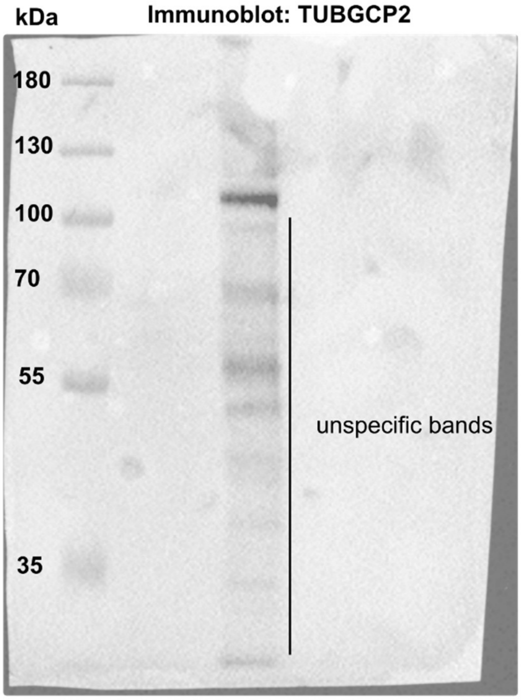

SFig. 1B uncropped blots

Immunoblot: PARP

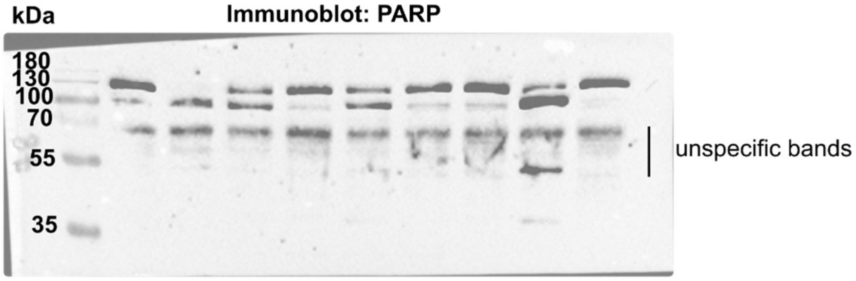

Immunoblot: GAPDH

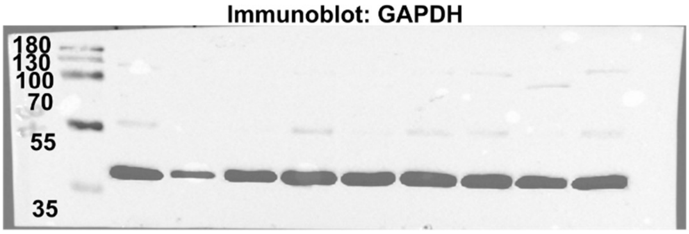

Supplementary Materials and Methods

Data acquisition for MS

MSn data were acquired in data independent acquisition (DIA) mode with base method m/z range of 100-1700 and 1/k0 range of 0.6-1.6 V×s×cm^{-2}. Precursor range was defined to m/z 400-1000 with equal windows sizes of 26 Th (including 1 Th overlap) using two steps each PASEF scan and cycle time of 100ms locked to 100% duty cycle.

DiaPASEF data were processed in DIA-NN[1] (version 1.8) in library free mode against modified cRAP database (based on http://www.thegpm.org/crap; 112 sequences in total) and UniProtKB protein database (RRID:SCR_004426) for Homo sapiens (downloaded 23.5.2023, number of protein sequences: 20,593). No variable, carbamidomethylation as fixed modification and trypsin/P enzyme with 1 allowed missed cleavages were set during the library preparation. False discovery rate (FDR) control was set to 1% FDR. MS1 and MS2 accuracies as well as scan window parameters were set based on the initial test searches (median value from all samples ascertained parameter values). MBR was switched on. Reported protein intensities were further processed using the software container environment (https://github.com/OmicsWorkflows). Processing workflow has been deposited on WorkflowHub registry (https://doi.org/10.48550/arXiv.2410.06941) with the access link https://doi.org/10.48546/workflowhub.workflow.1309.1. Briefly, it covered: a) removal of low-quality precursors and contaminant protein groups, b) protein group intensities log2 transformation, c) LoessF normalization, d) filtering out of protein groups not quantified in at least 2 replicates of at least one sample type and e) normalized intensities were used for differential expression using LIMMA statistical test. Ingenuity Pathway Analysis (RRID:SCR_008653) was run on Qiagen IPA 2023, based on proteins which were found to be significantly upregulated (adj. p-value<0.05) that were found in 4/4 biological replicates of CM39AL and control cells. The significance of the association between the data set and the canonical pathway was measured in two ways: 1) A ratio of the number of molecules from the data set that map to the pathway divided by the total number of molecules that map to the canonical pathway is displayed; and 2) A right-tailed Fisher's Exact Test was used to calculate a p-value determining the probability that the association between the genes in the dataset and the canonical pathway is explained by chance alone. 3) a z-score was calculated to indicate the likelihood of activation or inhibition of that pathway. Benjamini-Hochberg method was used for multiple testing correction.

In situ click-chemistry with Azide Fluor-488 and immunofluorescence staining

After treatment with CM39AL at 40 µM or DMSO for 2h, cells were spun at 800 rpm for 5 min using Cytospin3 on SuperFrost Plus Microscope Slides (ThermoFisher). Cells were fixed with 3.7% formaldehyde for 15 min at RT, then washed with PBS, dried and stored at -20°C until further processing. For *in situ* fluorescence tagging of CM39AL, CuAAC reaction mix containing Azide Fluor-488 (50µM, Sigma Aldrich) was pipetted on the Cytospin slides and incubated in the dark for 1.5 hours, then slides were washed three times with PBS. For further immunofluorescence staining, permeabilization was performed with 0.01% Triton-X (Biorad). Blocking was performed using 10% Goat Serum in PBS, then cells were washed three times with 0.1% Tween in PBS and incubated with primary (anti-TUBGCP2,# PA5-58151, RRID:AB_2641922; anti-

γ-Tubulin, Sigma-Aldrich T5326-25UL) at 1:200 in 1% BSA in PBS overnight at 4 °C in a humid chamber. Slides were washed three times with PBS-Tween and incubated with fluorophore-conjugated secondary antibody (goat anti-mouse Alexa-Fluor 594, Abcam Cat# ab150116, RRID:AB_2650601, goat anti-rabbit Cy5, Abcam Cat# ab6564, RRID:AB_955061) at 1:200 and DAPI at 1 µg/mL in 1% BSA in PBS. After washing, slides were mounted using Fluoromount-G (Invitrogen). Images were acquired using an Olympus/Evident IXplore spinning disk confocal microscope, equipped with the Yokogawa CSU-W1 SoRa disk and a Hamamatsu ORCA Fusion CMOS camera. A 60×oil immersion objective in combination with a 3.2×magnification lens was used. Z-stacks were acquired and then processed using Olympus/Evident cellSens V4.1.1 64bit software, applying the constrained iterative deconvolution algorithm. Finally, maximum intensity projection images of the deconvoluted z-stacks were obtained.

Flow cytometry

Cells were washed with Annexin-V binding buffer (10 mM HEPES pH 7.4, 140 mM NaCl, 2.5 mM CaCl2), then incubated with Alexa Fluor 488 Ready Flow Annexin-V (ThermoFisher) in binding buffer according to manufacturer instructions. Stained cells were diluted with 4 volumes of buffer containing 7-AAD (Biolegend) diluted 1:100. For cell cycle analysis, cells were fixed in 70% ice cold ethanol and incubated on ice for at least 4 hours. Cells were washed in PBS, then resuspended in 0.01% Triton X in PBS containing 10 µg/ml Propidium Iodide (MedChem) and 0.5 µg/mL RNAse I (Roche) and incubated at 37°C for 1 h. Samples were acquired on FACS Canto II and data were analyzed in FlowJo 7x (RRID:SCR_008520).

RNA Sequencing and analysis

The Mac-2a cell line was seeded in 3 biological replicates per condition. 300´000 cells in 3 ml of medium were treated with 2.5 µM CM14 or DMSO for 24h before harvesting. RNA was isolated using Qiagen RNeasy Mini Kit. RNA integrity was checked on the Fragment Analyzer using RNA Kit 15 nt (Agilent Technologies). 500 ng of total RNA was used as input for library preparation using QuantSeq FWD 3'mRNA Library Prep Kit (Lexogen) in combination with UMI Second Strand Synthesis Module for QuantSeq FWD andLexogen i5 6 nt Unique Dual Indexing Add-on Kit (Lexogen). Quality control for library quantity and size distribution was done using QuantiFluor dsDNA System (Promega) and High Sensitivity NGS Fragment Analysis Kit (Agilent Technologies). Final library pool was sequenced on NextSeq 500 using High Output Kit v2.5 75 Cycles (Illumina) in single-end mode, resulting in an average of 10 million reads per sample. Bcl files were converted to Fastq format using bcl2fastq v. 2.20.0.422 Illumina software for basecalling. Quality check of raw single-end fastq reads was carried out by FastQC. The adapters and quality trimming of raw fastq reads was performed using Trimmomatic v0.39 (RRID:SCR_011848) with settings CROP:250 LEADING:3 TRAILING:3 SLIDINGWINDOW:4:5 MINLEN:35. Trimmed RNA-Seq reads were mapped against the human genome (hs38) and Ensembl GRCh38-p10 annotation using STAR v2.7.3a as splice-aware short read aligner and default parameters except --outFilterMismatchNoverLmax 0 and --twopassMode Basic. Quality control after alignment concerning the number and percentage of uniquely- and multi-mapped reads, rRNA contamination, mapped regions, read coverage distribution, strand

specificity, gene biotypes and PCR duplication was performed using several tools namely RSeQC v4.0.0, Picard toolkit v2.25.6 (RRID:SCR_006525), Qualimap v.2.2.2. The differential gene expression analysis was calculated based on the gene counts produced using featureCounts from Subread package v2.0 and further analyzed by Bioconductor package (RRID:SCR_006442) DESeq2 v1.34.0 (RRID:SCR_000154). DESeq2 function performs an estimation of the data size factors by using the median ratio method. Then dispersion estimates for Negative Binomial distributed data is computed, and finally Wald test p-values is calculated. (Love, Huber, and Anders 2014). Data generated by DESeq2 with independent filtering were selected for the differential gene expression analysis due to its conservative features and to avoid potential false positive results. Benjamini-Hochberg method was used to correct for multiple testing. Genes were considered as differentially expressed based on a cut-off of adjusted p-value<0.05 and log2(fold-change) ≥1 or ≤-1. Volcano plots were produced using ggplot2 v3.3.5 package. For Ingenuity Pathway Analysis, core analysis was run on Qiagen IPA 2023 including differentially expressed genes with adj. p-value<0.05. The significance of the association between the data set and the canonical pathway was measured in two ways: 1) A ratio of the number of molecules from the data set that map to the pathway divided by the total number of molecules that map to the canonical pathway is displayed; and 2) A right-tailed Fisher's Exact Test was used to calculate a p-value determining the probability that the association between the genes in the dataset and the canonical pathway is explained by chance alone. 3) a z-score was calculated to indicate the likelihood of activation or inhibition of that pathway. Benjamini-Hochberg method was used for multiple testing correction.

Molecular docking

For the molecular dynamics (MD) simulations, the Gromacs program was used [2,3]. To model the intra- and intermolecular interactions of the Charmm36 force field [4] was employed for the protein, ligand, and the ions, and the TIP3P model [5] for the water. The MD simulations were all performed with a time step of 2 fs, and with the bonds involving hydrogens constrained. The structure of GCP2 was taken from the GCP2-GCP3 complex (pdb code: 6V6B) [6]. The protein was placed into a periodic box, and was solvated by water molecules. Sodium ions were added to the solution to neutralize the negative charge of the biomolecule. Thereafter, an energy minimization, and an initial MD run in an NVT ensemble were performed (T = 310 K) for 100 ps, followed by an isothermal-isobaric setup (T = 310 K; p = 1 bar) for another 100 ps. A production run in an NVT ensemble was then performed for 10 ns. In all simulations, a modified Berendsen thermostat, and (in case of NpT runs) a Parrinello-Rahman barostat was applied. The obtained structure of the protein was then taken out of the solvent to undergo molecular docking with the appropriate ligands, using CB-Dock2 [7,8]. The structures with the highest scores were then used for refinement with subsequent MD simulations, repeating the solvation, neutralization, energy minimization, NVT, NpT, and production runs, as described above.

Statistical analysis

Unpaired Student´s t test was performed for viability and cell cycle analysis using GraphPad Prism 8. A p-value <0.05 was considered as threshold for statistical significance. For RNA-seq and LS-MS/MS details about statistical analysis can be found in the corresponding sections.

General synthetic experimental procedures

All reactions were carried out under an argon atmosphere in oven-dried glassware. All reagents and chemicals were purchased from commercial suppliers and used without further purification unless otherwise noted. All purification procedures were carried out with reagent-grade solvents in air. Thin layer chromatography (TLC) analysis was conducted using silica gel-coated aluminum sheets (SiliaPlate TLC, Silicycle®) with detection by UV light (254 nm, UVS-11, Mineralight® shortwave UV lamp). Purification was carried out by flash chromatography (Isco, Inc. CombiFlash™ Sg100c). ^{1}H and ^{13}C nuclear magnetic resonance (NMR) spectra were recorded at room temperature using Bruker AV-III-400 spectrometer. Chemical shifts (δ values) were reported in parts per million and were referenced to the deuterated residual solvent peak. NMR data were reported as δ value (where s = singlet, d = doublet, t = triplet, q = quartet, and quin = quintuplet, integration, J value (Hz)). The value of the coupling constant (J = 15-16 Hz) confirms the trans stereochemistry of α,β-unsaturation. High-resolution mass spectrometry (HRMS) measurements were performed on an Agilent 6200 high-resolution time-of-flight mass spectrometer equipped with a Dual ESI ion source. Analytical high-performance liquid chromatography (HPLC) was performed on an Agilent Technologies system (Agilent1100 Series) with an ACE C18 column (150 mm x 4.6 mm, 5µ) using miliQ water (A) and HPLC grade methanol (B) as mobile phase in a gradient mode (gradient steps: 75:25 (A:B; 1 min), 15:85 (A:B; 7 min) and 75:25 (A:B; 7 min); flow rate of 1.0 ml/min, detection at 320 nm). The purity of >98% has been established for all tested compounds.

Esters analogs were synthesized either by our optimized one-step esterification of hydroxycinnamic acids or via the Witting coupling using the appropriate aldehyde and stabilized phosphonium ylide[9–11]. Ketone analogs were synthesized through an aldol condensation with the appropriate benzaldehyde and acetone[9,12]. Ketones (**K1**) and (**K1**) required for the synthesis of **CM1** ketone analogs are described in schemes 1 and 2.

General procedure 1: **Synthesis of ester analogs**

Hydroxycinnamic acid (1 eq) was added to a vigorously stirred solution of Na_2CO_3 (1.5 eq) in hexamethylphosphoramide (HMPA) and the reaction vessel was flushed with argon. After 30 min of stirring at 0°C in an ice bath, the appropriate bromide (1.2 eq) was added dropwise to the reaction mixture over a period of 10 min. A catalytic amount of KI was added to the reaction vessel, which is then thoroughly flushed with argon gas and sealed under balloon pressure. The reaction mixture was stirred in an ice bath for 2h and at room temperature for 24h under argon atmosphere. The resulting solution was quenched with ice/water (200 mL) and stirred for 30 min. The aqueous phase was extracted with ethyl acetate (EtOAc) (3 x 50 mL). The combined organic fractions were then washed with brine (3 x 50 mL), dried over $MgSO_4$, and concentrated under vacuum. The resulting crud product was purified by flash chromatography.

Syntheses and characterizations of literature unknown esters analogs:

Compound CM10

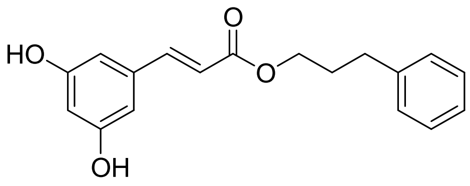

Following general procedure 1 with trans 3,5-dihydroxycinnamic acid[13] (250 mg, 1.39 mmol, 1 eq), Na_2CO_3 (220 mg, 2.07 mmol, 1.5 eq), 3-phenylpropyl bromide (255 µl, 1.66 mmol, 1.2 eq), and 7 mL of HMPA, compound **CM11** was obtained as a white solid after flash chromatography (EtOAc/Hexane (1/9) to EtOAc/Hexane (1/1), yield = 72 %, m.p. 112-114°C. ^{1}H NMR (400 MHz, DMSO) δ 9.45 (s, 2H, 2 x OH), 7.43 (d, J = 15.9 Hz, 1H, ArCH=), 7.30 (t, J = 7.5 Hz, 2H, H_{ar}), 7.26 – 7.22 (m, 2H, H_{ar}), 7.22 – 7.15 (m, 1H, H_{ar}), 6.51 (d, J = 2.4 Hz, 2H, H_{ar}), 6.38 (d, J = 15.9 Hz, 1H, COCH=), 6.32 (q, J = 2.2 Hz, 1H, H_{ar}), 4.13 (t, J = 6.5 Hz, 2H, OCH_2), 2.69 (t, J = 7.6 Hz, 2H, $ArCH_2$), 2.01 – 1.90 (m, 2H, $OCH_2C\underline{H_2}$). ^{13}C NMR (100 MHz, $CDCl_3$), δ 166.67, 159.16, 145.45, 141.66, 136.08, 128.80, 128.78, 126.34, 117.94, 106.75, 105.37, 63.86, 31.95, 30.29. HRMS m/z calc. for $C_{18}H_{18}O_4$ + (H^+): 299.1278; found: 299.1284.

Compound CM11

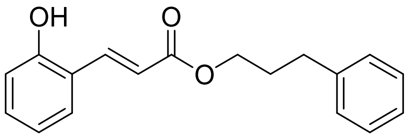

Following general procedure 1 with trans 2-hydroxycinnamic acid (300 mg, 1.83 mmol, 1 eq), Na_2CO_3 (290 mg, 2.73 mmol, 1.5 eq), 3-phenylpropyl bromide (333 µL, 2.19 mmol, 1.2 eq), and 7 mL of HMPA, compound **CM11** was obtained as a white solid after flash chromatography (EtOAc/Hexane (1/9) to EtOAc/Hexane (1/1), yield = 77 %, m.p. 93-95°C. ^{1}H NMR (400 MHz, DMSO) δ 10.25 (s, 1H, OH), 7.89 (d, J = 16.1 Hz, 1H, ArCH=), 7.61 (d, J = 1.7 Hz, 1H, H_{ar}), 7.35 – 7.13 (m, 6H, H_{ar}), 6.93 (d, J = 8.9 Hz, 1H, H_{ar}), 6.84 (t, J = 7.5 Hz, 1H, H_{ar}), 6.63 (d, J = 16.1 Hz, 1H, COCH=), 4.13 (t, J = 6.6 Hz, 2H, OCH_2), 2.69 (t, J = 7.7 Hz, 2H, $ArCH_2$), 1.95 (dt, J = 13.7, 6.7 Hz, 2H, $OCH_2C\underline{H_2}$). ^{13}C NMR (100 MHz, $CDCl_3$), δ 167.23, 157.25, 141.63, 140.58, 132.17, 129.37, 128.81, 128.76, 126.34, 121.18, 119.87, 117.57, 116.63, 63.67, 31.93, 30.35. HRMS m/z calc. for $C_{18}H_{18}O_3$ + (H^+): 283.1329; found: 283.1330.

Compound CM12

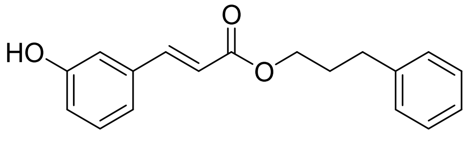

Following general procedure 1 with trans 3-hydroxycinnamic acid (300 mg, 1.83 mmol, 1 eq), Na_2CO_3 (290 mg, 2.73 mmol, 1.5 eq), 3-phenylpropyl bromide (333 µL, 2.19 mmol, 1.2 eq), and 7 mL of HMPA, compound **CM12** was obtained as a white solid after flash chromatography (EtOAc/Hexane (1/9) to EtOAc/Hexane (1/1), yield = 93 %, m.p.: 88-90°C. ^{1}H NMR (400 MHz, DMSO) δ 9.62 (s, 1H, OH), 7.55 (d, J = 16.0 Hz, 1H, ArCH=), 7.30 (t, J = 7.3 Hz, 2H, H_{ar}), 7.26 – 7.12 (m, 5H, H_{ar}), 7.05 (s, 1H, H_{ar}), 6.85 (d, J = 7.9 Hz, 1H, H_{ar}), 6.52 (d, J = 16.0 Hz, 1H, COCH=), 4.14 (t, J = 6.5 Hz, 2H, OCH_2), 2.74

– 2.65 (m, 2H, $ArCH_2$), 2.02 – 1.90 (m, 2H, $OCH_2C\underline{H_2}$). ^{13}C NMR (100MHz, $CDCl_3$), δ 166.69, 158.17, 145.13, 141.65, 135.73, 130.39, 128.81, 128.78, 126.34, 119.71, 118.27, 118.10, 115.19, 63.88, 31.95, 30.29. HRMS m/z calc. for $C_{18}H_{18}O_3$ + (Na^+): 305.1148; found: 305.1149.

Compound CM13

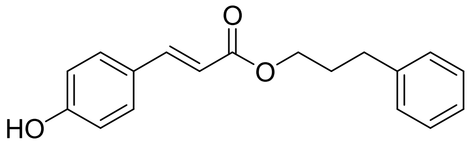

Following general procedure 1 with trans 4-hydroxycinnamic acid (300 mg, 1.83 mmol, 1 eq), Na_2CO_3 (290 mg, 2.73 mmol, 1.5 eq), 3-phenylpropyl bromide (333 µL, 2.19 mmol, 1.1 eq), and 7 mL of HMPA, compound **CM13** was obtained as a white solid after flash chromatography (EtOAc/Hexane (1/9) to EtOAc/Hexane (1/1), yield = 70 %, m.p.: 89-90°C. ^1H NMR (400 MHz, DMSO) δ 10.01 (s, 1H, OH), 7.64 – 7.50 (m, 3H, H_{ar}, ArCH=), 7.30 (t, J = 7.5 Hz, 2H, H_{ar}), 7.26 – 7.15 (m, 3H, H_{ar}), 6.80 (d, J = 8.6 Hz, 2H, H_{ar}), 6.41 (d, J = 16.0 Hz, 1H, COCH=), 4.12 (t, J = 6.5 Hz, 2H, OCH_2), 2.73 – 2.64 (m, 2H, $ArCH_2$), 2.02 – 1.88 (m, 2H, $OCH_2C\underline{H_2}$). ^{13}C NMR (100MHz, $CDCl_3$) δ 167.10, 160.30, 145.14, 141.67, 130.79, 128.81, 128.77, 126.33, 125.56, 116.22, 114.62, 63.59, 31.96, 30.36. HRMS m/z calc. for $C_{18}H_{18}O_3$ + (H^+): 283.1329; found: 283.1326.

General procedure 2: Synthesis of ketone analogs

To a stirred solution of the appropriate hydroxybenzaldehyde (1 eq) and the appropriate ketone (1.1 eq) in tetrahydrofuran (THF) was added successively a catalytic amount of acetic acid (4 drops) and 100 µL of pyrrolidine. The solution was refluxed under argon atmosphere for 12h. After the removal of under vacuum, water (50 mL) was added followed by extractions with EtOAc (3 x 25 mL). The combined organic fractions were then combined and stirred with sodium bisulfite ($NaHSO_3$) solution (100 mL, 2M) for 30 min to remove any traces of starting aldehyde. After separation, the combined organic fractions were washed with brine (3 x 50 mL), dried over $MgSO_4$, filtered, and concentrated under vacuum. The resulting crud product was purified by flash chromatography.

Syntheses and characterizations of literature unknown ketones analogs

Ketone (**k1**) required for the synthesis of **CM3**-**CM21** analogs was synthesized as described in the literature (Scheme 1)[14], with some changes, notably in the hydrogenation step.

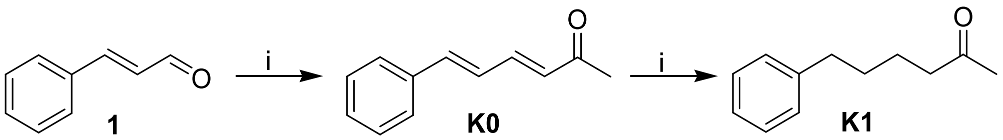

Scheme 1. Reagents and conditions: (i) Acetone, reflux; (ii) Pd/c (5%), H_2, 2.75 MPa, 30 min.

Synthesis of (3E,5E)-6-phenylhexa-3,5-dien-2-one (**K0**)

To a stirred solution of cinnamaldehyde (**1**) (7.9 g, 60 mmol, 1 eq) and acetone (13.2 mL, 178 mmol, 3 eq) in water was added NaOH (1.2 g, 30 mmol, 0.5 eq). The solution was heated at 70°C for 8 h. The white solid obtained after coolness of the solution was dissolved in EtOAC (250 mL) was added followed by extractions with EtOAc (3 x 25 mL). The combined organic fractions were then combined and stirred with sodium bisulfite ($NaHSO_3$) solution (100 mL, 2M) for 30 min to remove any traces of the starting aldehyde. After separation, the combined organic fractions were washed with brine (3 x 50 mL), dried over $MgSO_4$, filtered, and concentrated under vacuum. The obtained yellow solid (yield = 92 %, m.p. 57-59°C) was analyzed by NMR after evaporation of the solvent and used in the next step without any further purification. ^{1}H NMR (400 MHz, CDCl3) δ 7.50 (d, J = 6.7 Hz, 2H, H_{ar}), 7.43 – 7.30 (m, 4H, 3H_{ar}, CH=), 7.02 – 6.85 (m, 2H, CH=), 6.29 (d, J = 15.5 Hz, 1H, CH=), 2.34 (s, 3H, CH_3).^{13}C NMR (101 MHz, $CDCl_3$) δ 198.44, 143.45, 141.29, 135.98, 130.51, 129.24, 128.87, 127.26, 126.67, 27.39.

Synthesis of 6-phenylhexan-2-one (**K1**)

In high-pressure autoclave[15] equipped with a glass liner containing a stirring bar, were introduced (3E,5E)-6-phenylhexa-3,5-dien-2-one (**2**) (2 g, 11.6 mmol, 1 eq), Pd/C (5 wt %, 100 mg, 0.047 mmol, 0.004 eq of Pd) and EtOAc (15 mL). The autoclave was pressurized at 2.75 MPa at room temperature and the mixture is stirred for 1h. At the end of hydrogen consumption, the mixture was filtered under a flow of nitrogen through a Celite pad. The obtained colorless liquid (yield = 98 %) was analyzed by NMR after evaporation of the solvent and used in the next step without any further purification.

^{1}H NMR (400 MHz, $CDCl_3$) δ 7.33 – 7.26 (m, 2H, H_{ar}), 7.22 – 7.18 (m, 3H, H_{ar}), 2.66 (d, *J* = 6.7 Hz, 2H, $ArCH_2$), 2.50 – 2.43 (m, 2H, $COCH_2$), 2.14 (s, 3H, CH_3), 1.68 – 1.61 (m, 4H, $ArCH_2\underline{CH_2CH_2}CH_2CO$). ^{13}C NMR (101 MHz, $CDCl_3$) δ 209.00, 142.19, 128.38, 128.32, 125.77, 43.58, 35.73, 30.95, 29.89, 23.47.

Compound CM3

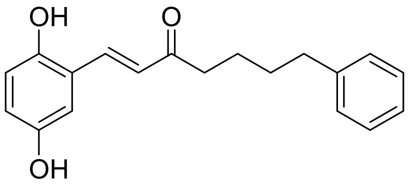

Following general procedure 2 with trans 2,5-dihydroxybenzaldehyde (250 mg, 1.8 mmol, 1 eq), 6-phenylhexan-2-one (**K1**) (350 mg, 2 mmol, 1.1 eq) and 10 mL of THF, compound **CM3** was obtained as a green solid after flash chromatography (EtOAc/Hexane (1/9) to EtOAc/Hexane (1/1), yield = 66 %, m.p.: 115-117°C. ^{1}H NMR (400 MHz, DMSO) δ 9.50 (s, 1H, OH), 8.89 (s, 1H, OH), 7.74 (d, J = 16.3 Hz, 1H, ArCH=), 7.29 – 7.26 (m, 2H, H_{ar}), 7.24 – 7.12 (m, 3H, H_{ar}), 6.94 (d, J = 2.7 Hz, 1H, H_{ar}), 6.78 – 6.67 (m, 3H, 2H_{ar}, COCH=), 2.67 (t, J = 6.7 Hz, 2H, $COCH_2$), 2.60 (t, J = 6.9 Hz, 2H, $ArCH_2$), 1.65 – 1.50 (m, 4H, $OC\underline{H_2}C\underline{H_2}$). ^{13}C NMR (101 MHz, DMSO) δ 200.39, 150.40, 150.39, 142.55, 137.78, 128.74, 128.70, 126.11, 125.77, 121.63, 119.85, 117.48, 113.54, 40.30, 35.44, 31.01, 24.00. HRMS m/z calc. for $C_{19}H_{20}O_3$ + (H^+): 297.1485; found: 297.1480.

Compound CM4

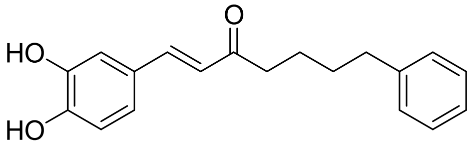

Following general procedure 2 with trans 3,4-dihydroxybenzaldehyde (250 mg, 1.8 mmol, 1 eq), 6-phenylhexan-2-one (**K1**) (350 mg, 2 mmol, 1.1 eq) and 10 mL of THF, compound **CM4** was obtained as a yellow solid after flash chromatography (EtOAc/Hexane (1/9) to EtOAc/Hexane (1/1), yield = 57 %, m.p. 105-107°C. ^{1}H NMR (400 MHz, DMSO) δ 9.59 (s, 1H, OH), 9.15 (s, 1H, OH), 7.44 (d, J = 16.1 Hz, 1H, ArCH=), 7.29 – 7.26 (m, 2H, H_{ar}), 7.20 – 7.15 (m, 3H, H_{ar}), 7.07 (s, 1H, H_{ar}), 7.01 (d, J = ^{8}.1 Hz, 1H, H_{ar}), 6.78 (d, J = 8.2 Hz, 1H, H_{ar}), 6.54 (d, J = 16.1 Hz, 1H, COCH=), 2.66 (t, J = 6.9 Hz, 2H, $COCH_2$) 2.60 (t, J = 6.9 Hz, 2H, $ArCH_2$), 1.64 – 1.51 (m, 4H, $COC\underline{H_2}C\underline{H_2}$). ^{13}C NMR (101 MHz, DMSO) δ 200.13, 148.86, 146.07, 143.20, 142.56, 128.74, 128.70, 126.33, 126.10, 123.60, 122.03, 116.26, 115.27, 39.88, 35.42, 31.03, 24.10. HRMS m/z calc. for $C_{19}H_{20}O_3$ + (H^+): 297.1485; found: 297.1481.

Compound CM15

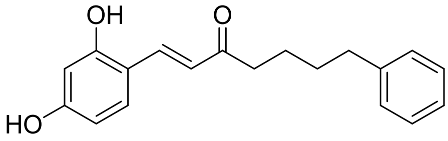

Following general procedure 2 with trans 2,4-dihydroxybenzaldehyde (356 mg, 2.57 mmol, 1eq), 6-phenylhexan-2-one (**K1**) (500 mg, 2.83 mmol, 1.1 eq) and 10 mL of THF, compound **CM15** was obtained as a green wax after flash chromatography (EtOAc/Hexane (1/9) to EtOAc/Hexane (1/1), yield = 54 %. ^{1}H NMR (400 MHz, DMSO) δ 10.11 (s, 1H, OH), 9.88 (s, 1H, OH), 7.71 (d, J = 16.3 Hz, 1H, ArCH=), 7.43 (d, J = 8.6 Hz, 1H, H_{ar}), 7.29 – 7.25 (m, 3H, H_{ar}), 7.20 – 7.15 (m, 4H, H_{ar}), 6.69 (d, J = 16.2 Hz, 1H, ArCH=), 6.40 – 6.34 (m, 1H), 6.28 (dd, J = 8.5, 2.4 Hz, 1H, H_{ar}), 2.63 – 2.58 (m, 4H, $COC\underline{H_2}$, $ArC\underline{H_2}$), 1.62 – 1.53 (m, 4H, $COCH2C\underline{H_2}C\underline{H_2}$). ^{13}C NMR (101 MHz, DMSO) δ 200.17, 161.50, 159.18, 142.57, 138.30, 130.52, 128.74, 128.69, 126.09, 122.65, 113.21, 108.35, 102.97, 40.12, 35.45, 31.08, 24.23. HRMS m/z calc. for $C_{19}H_{20}O_3$ + (H^+): 297.1485; found: 297.1483.

Compound CM16

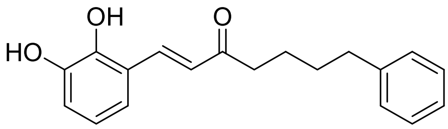

Following general procedure 2 with trans 2,3-dihydroxybenzaldehyde (356 mg, 2.57 mmol, 1 eq), 6-phenylhexan-2-one (**K1**) (500 mg, 2.83 mmol, 1.1 eq) and 10 mL of THF, compound **CM16** was obtained as a yellow-green solid after flash chromatography (EtOAc/Hexane (1/9) to EtOAc/Hexane (1/1), yield = 53 %, m.p.: 103-105°C. ^{1}H NMR (400 MHz, DMSO) δ 9.65 (s, 1H, OH), 9.11 (s, 1H, OH), 7.81 (d, J = 16.3 Hz, 1H, ArCH=), 7.29 – 7.26 (m, 3H, H_{ar}), 7.22 – 7.14 (m, 4H, H_{ar}), 7.07 (d, J = 7.3 Hz, 1H, H_{ar}), 6.84 – 6.80 (m, 2H, H_{ar}, COCH=), 6.67 (t, J = 7.8 Hz, 1H, H_{ar}), 2.71 – 2.65 (m, 2H, $COC\underline{H_2}$), 2.60 (t, J = 7.0 Hz, 2H, $ArC\underline{H_2}$), 1.61 – 1.58 (m, 4H, $COCH_2C\underline{H_2}C\underline{H_2}$). ^{13}C NMR (101 MHz, DMSO) δ 200.47, 146.11,

146.06, 142.55, 137.98, 128.75, 128.70, 126.12, 126.10, 122.06, 119.63, 118.91, 117.31, 35.44, 31.02, 24.00. HRMS m/z calc. for $C_{19}H_{20}O_3$ + (H^+): 297.1485; found: 297.1489.

Compound CM18

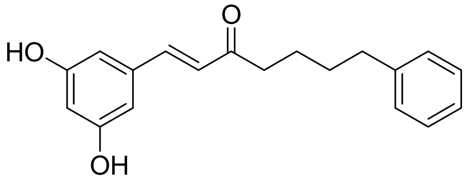

Following general procedure 2 with trans 3,5-dihydroxybenzaldehyde (356 mg, 2.57 mmol, 1 eq), 6-phenylhexan-2-one (**K1**) (500 mg, 2.83 mmol, 1.1 eq) and 10 mL of THF, compound **CM18** was obtained as a white solid after flash chromatography (EtOAc/Hexane (1/9) to EtOAc/Hexane (1/1), yield = 70 %, m.p.: 117-119°C. ^{1}H NMR (400 MHz, DMSO) δ 9.45 (s, 2H, OH), 7.40 (d, J = 16.1 Hz, 1H, ArCH=), 7.27 – 7.26 (m, 2H, H_{ar}), 7.21 -7.15 (m, 3H, H_{ar}), 6.63 (d, J = 16.2 Hz, 1H, COCH=), 6.51 (m, 2H, H_{ar}), 6.30 – 6.31 (m, 1H, H_{ar}), 2.70 (t, J = 6.6 Hz, 2H, $COCH_2$), 2.60 (t, J = 7.0 Hz, 2H, $ArCH_2$), 1.66 – 1.50 (m, 4H, $COCH_2\underline{CH_2}\underline{CH_2}$). ^{13}C NMR (101 MHz, DMSO) δ 200.43, 159.17, 142.92, 142.54, 136.58, 128.73, 128.69, 126.56, 126.10, 106.82, 105.36, 35.40, 30.95, 23.91. HRMS m/z calc. for $C_{19}H_{20}O_3$ + (H^+): 297.1485; found: 297.1496.

Compound CM19

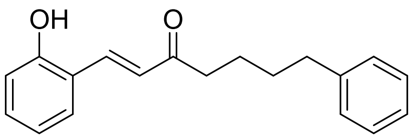

Following general procedure 2 with trans 2-hydroxybenzaldehyde (314 mg, 2.57 mmol, 1 eq), 6-phenylhexan-2-one (**K1**) (544 mg, 2.83 mmol, 1.1 eq) and 10 mL of THF, compound **CM19** was obtained as a white solid after flash chromatography (EtOAc/Hexane (1/9) to EtOAc/Hexane (1/1), yield = 48 %, m.p.: 98-100°C. ^{1}H NMR (400 MHz, DMSO) δ 10.21 (s, 1H, OH), 7.80 (d, J = 16.4 Hz, 1H, ArCH=), 7.61 (dd, J = 7.8, 1.7 Hz, 1H, H_{ar}), 7.31 – 7.23 (m, 3H, H_{ar}), 7.23 – 7.14 (m, 3H, H_{ar}), 6.96 – 6.81 (m, 3H, 2H_{ar}, COCH=), 2.73 – 2.64 (m, 2H, $COCH_2$), 2.60 (t, J = 6.8 Hz, 2H, $ArCH_2$), 1.65 – 1.52 (m, 4H, $COCH_2\underline{CH_2}\underline{CH_2}$). ^{13}C NMR (101 MHz, DMSO) δ 200.48, 157.41, 142.54, 137.72, 132.14, 129.01, 128.74, 128.70, 126.20, 126.10, 121.49, 119.89, 116.66, 40.33, 35.45, 31.02, 23.99. HRMS m/z calc. for $C_{19}H_{20}O_2$ + (H^+): 281.1536; found: 281.1541.

Compound CM20

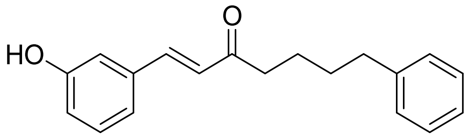

Following general procedure 2 with trans 3-hydroxybenzaldehyde (314 mg, 2.57 mmol, 1 eq), 6-phenylhexan-2-one (**K1**) (544 mg, 2.83 mmol, 1.1 eq) and 10 mL of THF, compound **CM20** was obtained as a white solid after flash chromatography

(EtOAc/Hexane (1/9) to EtOAc/Hexane (1/1), yield = 70 %, m.p. 92-94°C. ^{1}H NMR (400 MHz, DMSO) δ 9.62 (s, 1H, OH), 7.51 (d, J = 16.2 Hz, 1H, ArCH=), 7.31 – 7.22 (m, 2H, H_{ar}), 7.22 – 7.10 (m, 5H, H_{ar}), 7.05 (t, J = 2.0 Hz, 1H, H_{ar}), 6.88 – 6.81 (m, 1H, H_{ar}), 6.75 (d, J = 16.2 Hz, 1H, COCH=), 2.72 (t, J = 6.7 Hz, 2H, $COCH_2$), 2.60 (t, J = 6.9 Hz, 2H, $ArCH_2$), 1.58 (qt, J = 6.6, 3.4 Hz, 4H, $COCH_2C\underline{H_2}C\underline{H_2}$). ^{13}C NMR (101 MHz, DMSO) δ 200.46, 158.18, 142.59, 142.53, 136.21, 130.40, 128.74, 128.70, 126.79, 126.11, 119.83, 118.05, 115.13, 40.09, 35.41, 30.98, 23.88. HRMS m/z calc. for $C_{19}H_{20}O_2$ + (H^+): 281.1536; found: 281.1549.

Compound CM21

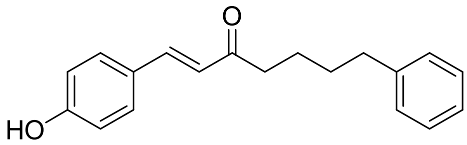

Following general procedure 2 with trans 4-hydroxybenzaldehyde (314 mg, 2.57 mmol, 1 eq), 6-phenylhexan-2-one (**K1**) (544 mg, 2.83 mmol, 1.1 eq) and 10 mL of THF, compound **CM21** was obtained as a yellow solid after flash chromatography (EtOAc/Hexane (1/9) to EtOAc/Hexane (1/1), yield = 70 %, m.p.: 82-84°C. ^{1}H NMR (400 MHz, DMSO) δ 10.02 (s, 1H, OH), 7.58 – 7.48 (m, 3H, 2H_{ar}, ArCH=), 7.27 (t, J = 7.4 Hz, 2H, H_{ar}), 7.22 – 7.14 (m, 3H, H_{ar}), 6.81 (d, J = 8.6 Hz, 2H, H_{ar}), 6.66 (d, J = 16.2 Hz, 1H, COCH=), 2.67 (t, J = 6.8 Hz, 2H, $COCH_2$), 2.60 (t, J = 7.0 Hz, 2H, $ArCH_2$), 1.64 – 1.53 (m, 4H, $COCH_2C\underline{H_2}C\underline{H_2}$). ^{13}C NMR (101 MHz, DMSO) δ 200.20, 160.30, 142.78, 142.55, 130.85, 128.74, 128.70, 126.10, 125.87, 123.73, 116.29, 39.93, 35.42, 31.04, 24.09. HRMS m/z calc. for $C_{19}H_{20}O_2$ + (X^+): 281.1536; found: 281.1544.

Synthesis of 5-phenylpentan-2-one (K2)

Ketone (**K2**) required for the synthesis of **CM14** analog was synthesized via high-pressure hydrogenation (scheme 2) instead of hydrogenation at balloon pressure as described in the literature[16].

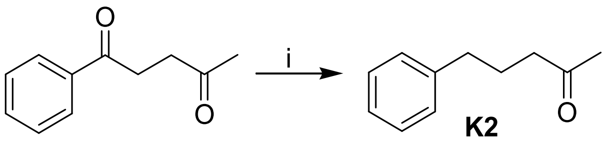

Scheme 2. Reagents and conditions: (i) Pd/C (10%), MeOH, H_2, 2.75 MPa, 24h.

Following the procedure described above for the synthesis of (**K1**) with 1-phenylpentane-1,4-dione (1 g, 5.67 mmol, 1 eq), Pd/C (10%) (300 mg, 0.28 mmol, 0.05 eq of Pd), and 10 mL of methanol (MeOH), 5-phenylpentan-2-one (**K2**) was obtained after filtration over a Celite pad and evaporation of the methanol. The yellow liquid (yield = 93 %) was analyzed by NMR and used in the next step without any further purification.

^{1}H NMR (400 MHz, $CDCl_3$) δ 7.33 – 7.29 (m, 2H, H_{ar}), 7.24 – 7.19 (m, 3H, H_{ar}), 2.65 (t, *J* = 7.5 Hz, 2H, $ArCH_2$), 2.46 (t, *J* = 7.4 Hz, 2H, $COCH_2$), 2.14 (s, 3H, CH_3), 1.94 (p, *J* = 7.5 Hz, 2H, $ArCH_2\underline{CH_2}CH_2CO$). ^{13}C NMR (101 MHz, $CDCl_3$) δ 208.79, 141.58, 128.47, 128.40, 125.97, 42.85, 35.03, 29.96, 25.21.

Compound CM14

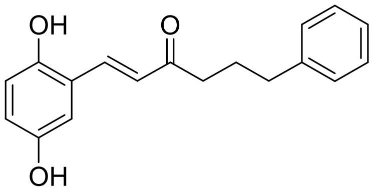

Following general procedure 2 with 2,5-dihydroxybenzaldehyde (314 mg, 2.57 mmol, 1eq), 5-phenylpentan-2-one (**K2**) (405 mg, 2.49 mmol, 1.1 eq) and 10 mL of THF, compound **CM14** was obtained as a green solid after flash chromatography (EtOAc/Hexane (1/9) to EtOAc/Hexane (1/1), yield = 48 %, m.p.: 82-85°C. ^{1}H NMR (400 MHz, DMSO) δ 9.51 (s, 1H, OH), 8.90 (s, 1H, OH), 7.73 (d, J = 16.3 Hz, 1H, ArCH=), 7.34 – 7.14 (m, 5H, H_{ar}), 6.94 (s, 1H, H_{ar}), 6.79 – 6.67 (m, 3H, $2H_{ar}$, COCH=), 2.66 (t, J = 6.9 Hz, 2H, $COCH_2$), 2.59 (d, J = 7.4 Hz, 2H, $ArCH_2$), 1.86 (p, J = 7.5 Hz, 2H, $COCH_2\underline{CH_2}CH_2$). ^{13}C NMR (101 MHz, DMSO) δ 200.17, 150.40, 142.28, 137.81, 128.77, 126.25, 125.70, 121.61, 119.88, 117.48, 113.53, 35.00, 26.16. HRMS m/z calc. for $C_{18}H_{18}O_3$ + (H^+): 283.1329; found: 283.1337.

Synthesis of 4-(4-(prop-2-ynyloxy)phenyl)butan-2-one K4

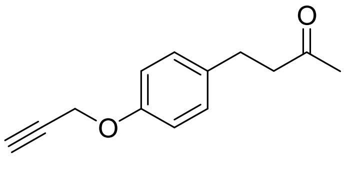

Ketone **K4** with the alkenyl moiety, required for the synthesis of **CM39AL** analog was synthetized as outlined in scheme 3.

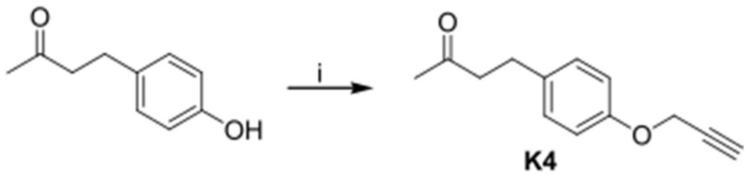

Scheme 3. Reagents and conditions : i) : propargyl bromide, K_2CO_3, DMF, 80°C, 12 h.

To a stirred solution of 4-(4-hydroxyphenyl)butan-2-one (1 g, 6.1 mmol, 1 eq) and 1-azido-3-chloropropane[17] (1.1 g, 9.1 mmol, 1.1 eq) in dimethylformamide (DMF) (10 mL) was added K_2CO_3 (1.26 g, 9.1 mmol, 1.5 eq). The solution was heated at 80°C under argon atmosphere for 12 h. Water (50 mL) was added followed by extractions with EtOAc (3 x 25 mL). The combined organic fractions were then combined were washed with brine (3 x 50 mL), dried over $MgSO_4$, filtered, and concentrated under vacuum. The resulting crud product was purified by flash chromatography. 4-(4-(prop-2-ynyloxy)phenyl)butan-2-one (**K4**) was obtained as a yellow oil after flash chromatography (Hexane (1/9) to EtOAc/Hexane

(1/9), yield = 81 %. ^{1}H NMR (400 MHz, CDCl3) δ 7.13 (d, J = 8.6 Hz, 2H, H_{ar}), 6.92 (d, J = 8.7 Hz, 2H, H_{ar}), 4.68 (d, J = 2.4 Hz, 2H, OCH_2), 2.92 – 2.81 (m, 2H, $ArCH_2$), 2.81 – 2.68 (m, 2H, $COCH_2$), 2.53 (t, J = 2.4 Hz, 1H, CH), 2.15 (s, 3H, CH_3). ^{13}C NMR (101 MHz, $CDCl_3$) δ 208.04, 155.96, 134.05, 129.27, 114.96, 78.70, 75.42, 55.87, 45.35, 30.11, 28.88. HRMS m/z calc. for $C_{13}H_{14}O_2$ + (H^+): 203.1067; found: 203.1051.

Compound CM39AL

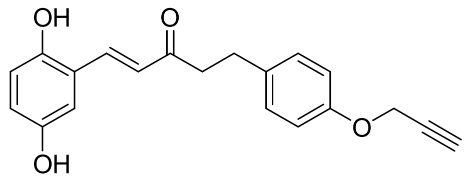

Following general procedure 2 with trans 2,5-dihydroxybenzaldehyde (250 mg, 1.81 mmol, 1 eq), 4-(4-(prop-2-ynyloxy)phenyl)butan-2-one (**K4**) (403 mg, 2 mmol, 1.1 eq) and 10 mL of THF, compound **CM39AL** was obtained as a green solid after flash chromatography (EtOAc/Hexane (1/9) to EtOAc/Hexane (1/1), yield = 58 %. mp: 99-101 °C. ^{1}H NMR (400 MHz, DMSO) δ 9.50 (s, 1H, OH), 8.90 (s, 1H, OH), 7.75 (d, J = 16.3 Hz, 1H, ArCH=), 7.18 (d, J = 8.6 Hz, 2H, H_{ar}), 6.94 (d, J = 2.7 Hz, 1H, Har), 6.90 (d, J = 8.7 Hz, 2H, H_{ar}), 6.77 (d, J = 16.3 Hz, 1H, COCH=), 6.74 – 6.68 (m, 2H, H_{ar}), 4.75 (d, J = 2.4 Hz, 2H, OCH_2), 3.53 (t, J = 2.4 Hz, 1H, CH), 2.95 (t, J = 7.7 Hz, 2H, $CH\underline{CH_2}$), 2.82 (t, J = 7.4 Hz, 2H, $ArCH_2$). ^{13}C NMR (101 MHz, DMSO) δ 199.55, 155.90, 150.43, 150.40, 137.93, 134.49, 129.71, 125.63, 121.62, 119.91, 117.50, 115.15, 113.53, 79.91, 78.51, 55.81, 42.39, 29.18. HRMS m/z calc. for $C_{20}H_{18}O_4$ + (H^+): 323.1278; found: 323.1266.

NMR, HRMS, and HPLC of literature unknown esters analogs

1H NMR

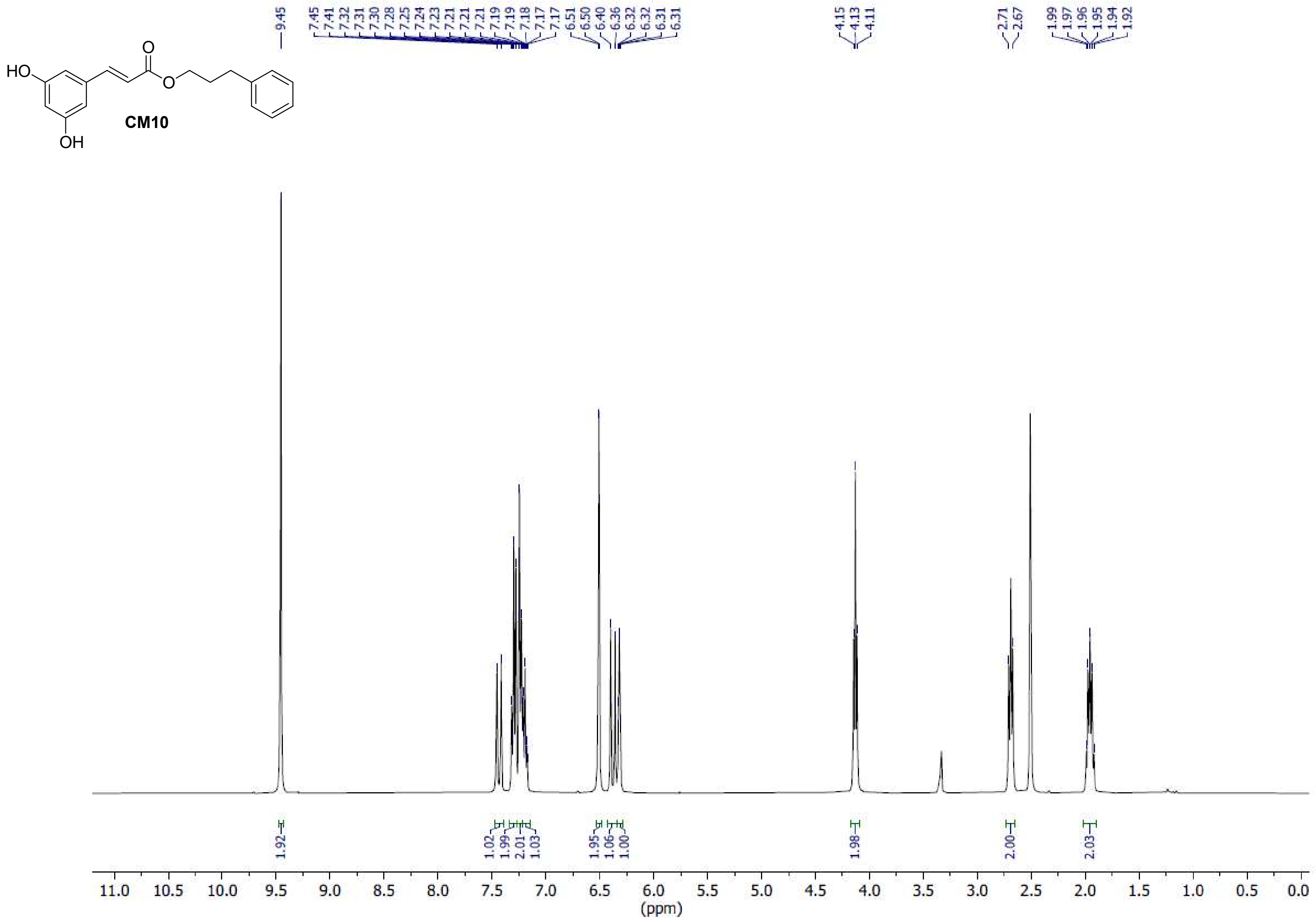

13C NMR

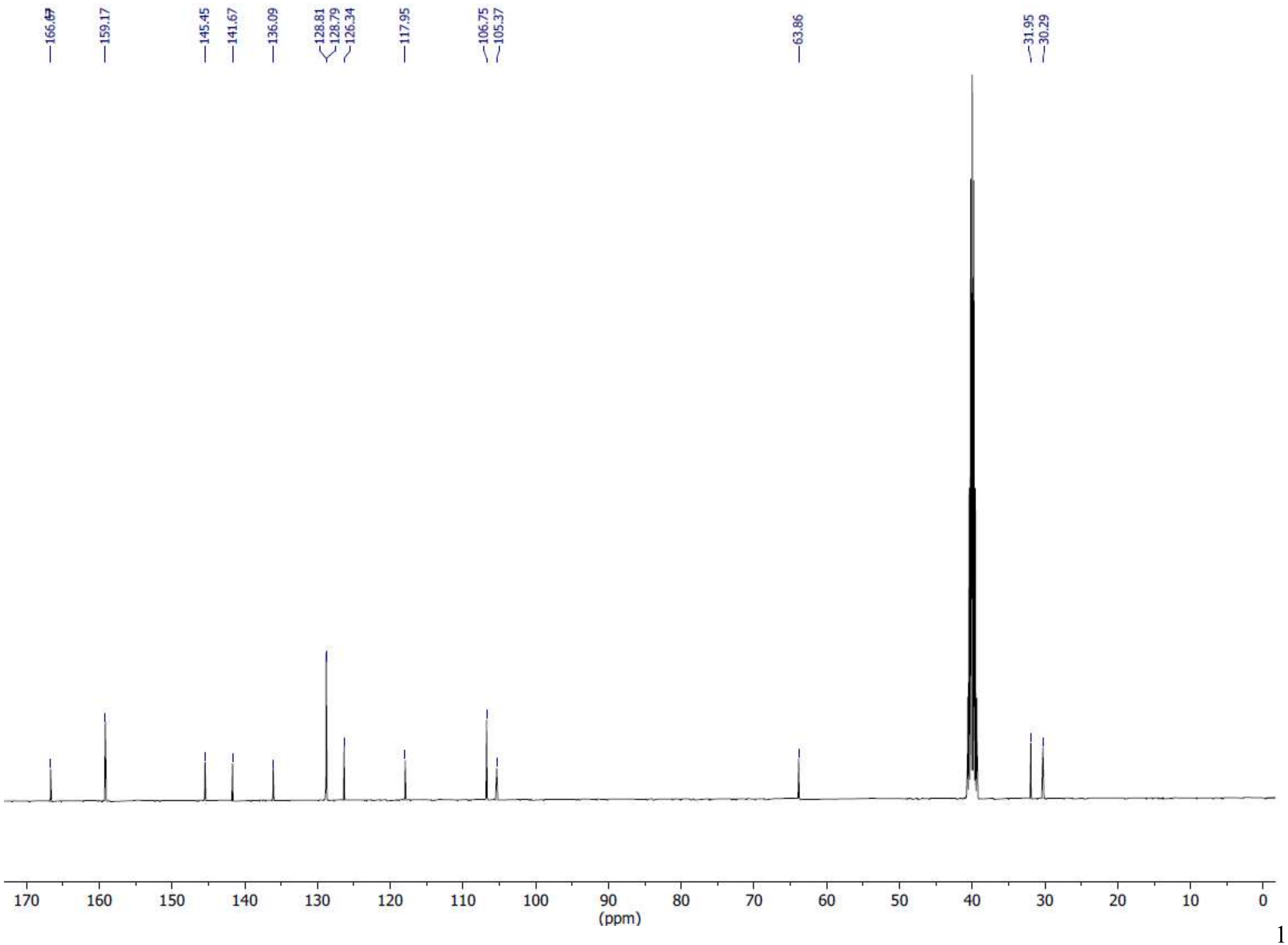

166.67
159.17
145.45
141.67
136.09
128.81
128.79
126.34
117.95
106.75
105.37
63.86
31.95
30.29
170
160
150
140
130
120
110
100
90
(ppm)
80
70
60
50
40
30
20
10
0

HRMS

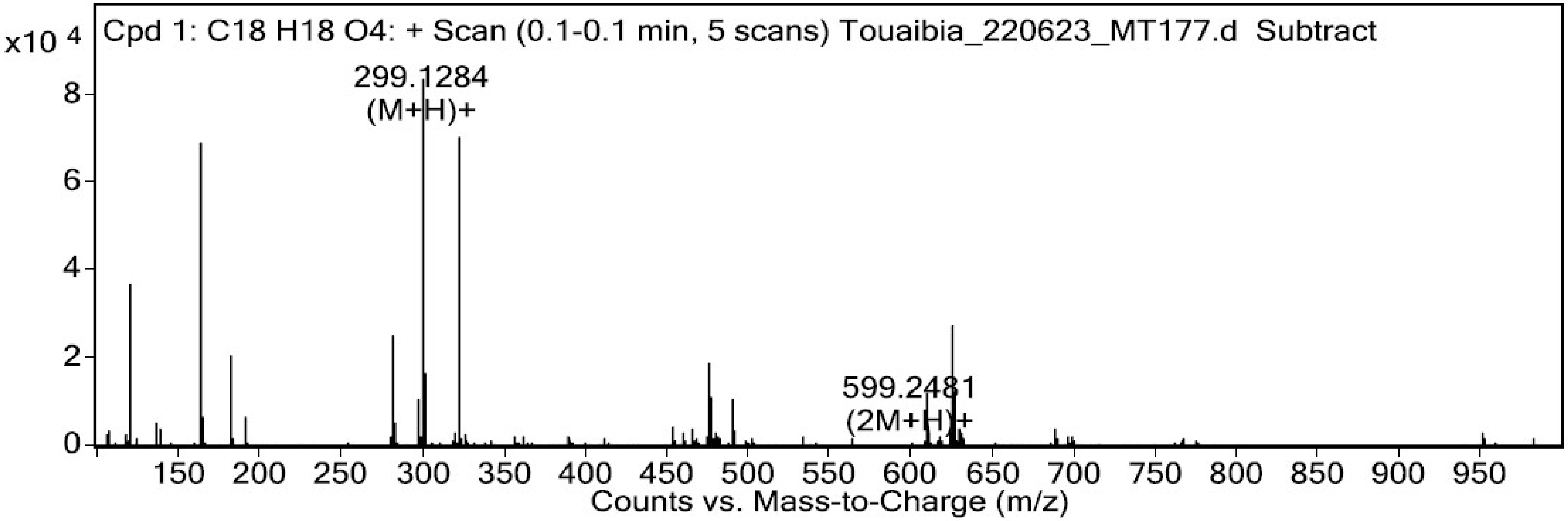

x10 4
Cpd 1: C18 H18 O4: + Scan (0.1-0.1 min, 5 scans) Touaibia_220623_MT177.d Subtract
299.1284
(M+H)+
599.2481
(2M+H)+
Counts vs. Mass-to-Charge (m/z)

HPLC

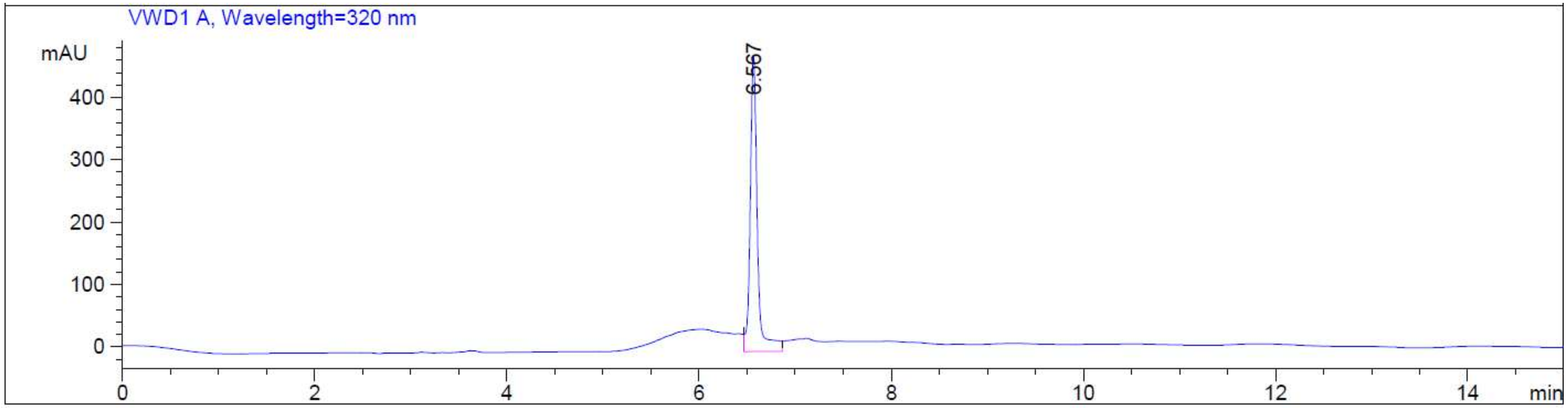

VWD1 A, Wavelength=320 nm
mAU
6.567
min

1H NMR

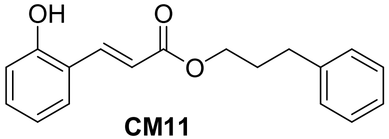

OH
O
O

CM11

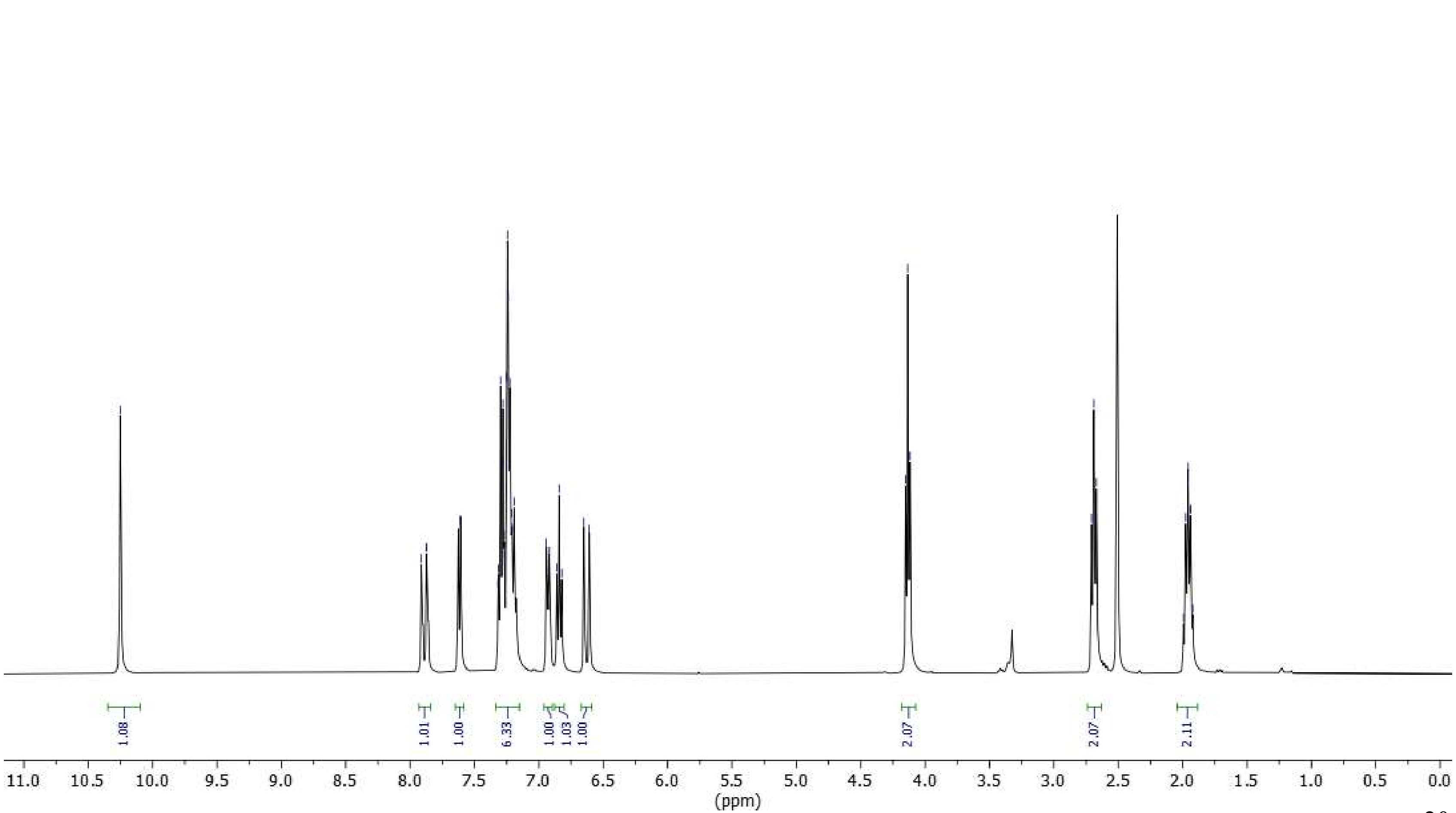

10.25
7.91
7.87
7.61
7.61
7.32
7.31
7.30
7.28
7.28
7.27
7.27
7.25
7.24
7.24
7.23
7.21
7.21
7.19
6.94
6.92
6.86
6.84
6.82
6.65
6.61
4.15
4.13
4.12
2.71
2.69
2.67
1.99
1.98
1.96
1.96
1.94
1.92
1.08
1.01
1.00
6.33
1.00
1.03
1.00
2.07
2.07
2.11
11.0
10.5
10.0
9.5
9.0
8.5
8.0
7.5
7.0
6.5
6.0
5.5
5.0
4.5
4.0
3.5
3.0
2.5
2.0
1.5
1.0
0.5
0.0
(ppm)

13C NMR

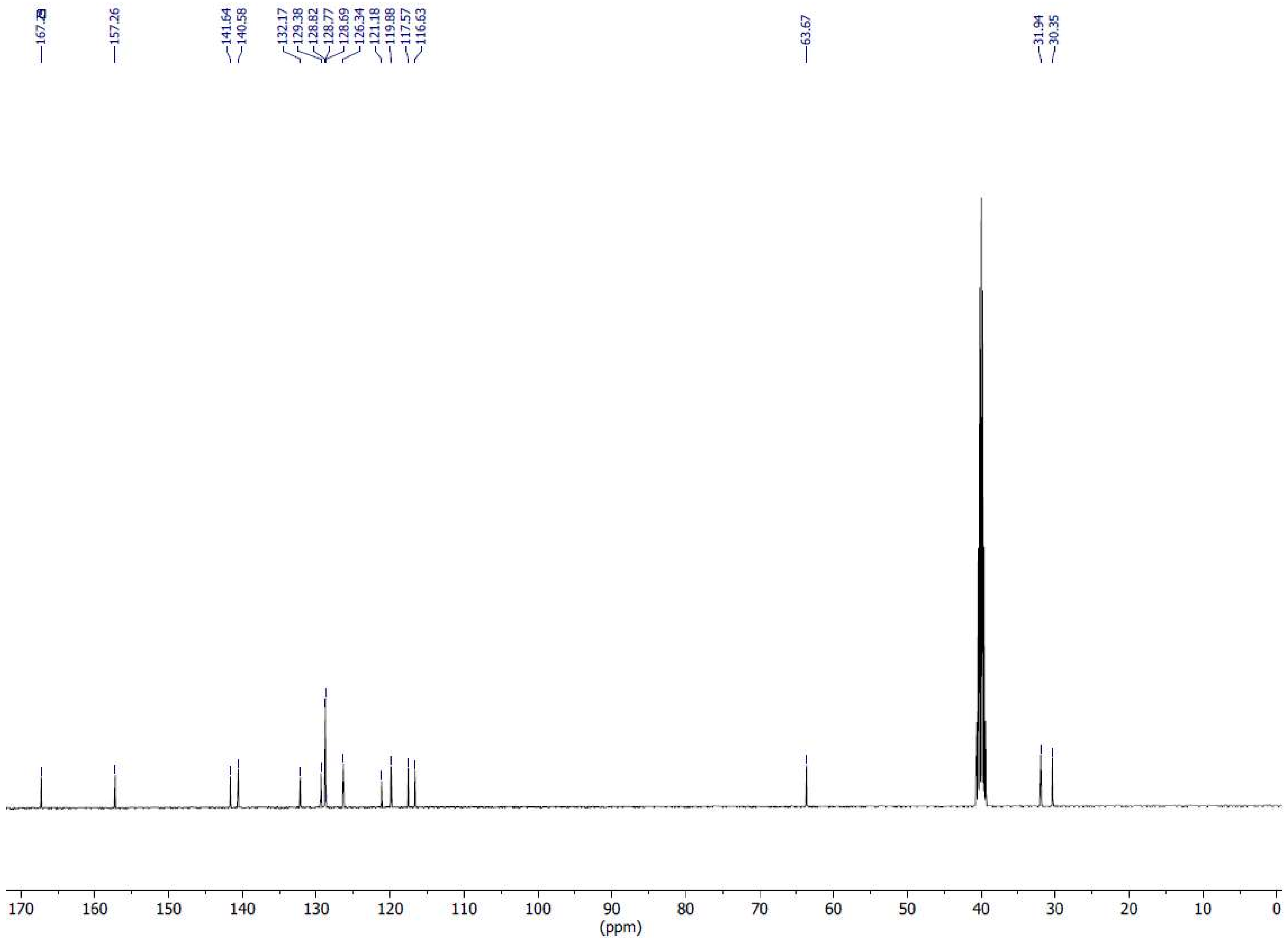

167.29
157.26
141.64
140.58
132.17
129.38
128.82
128.77
128.69
126.34
121.18
119.88
117.57
116.63
63.67
31.94
30.35
170
160
150
140
130
120
110
100
90
80
70
60
50
40
30
20
10
0
(ppm)

HRMS

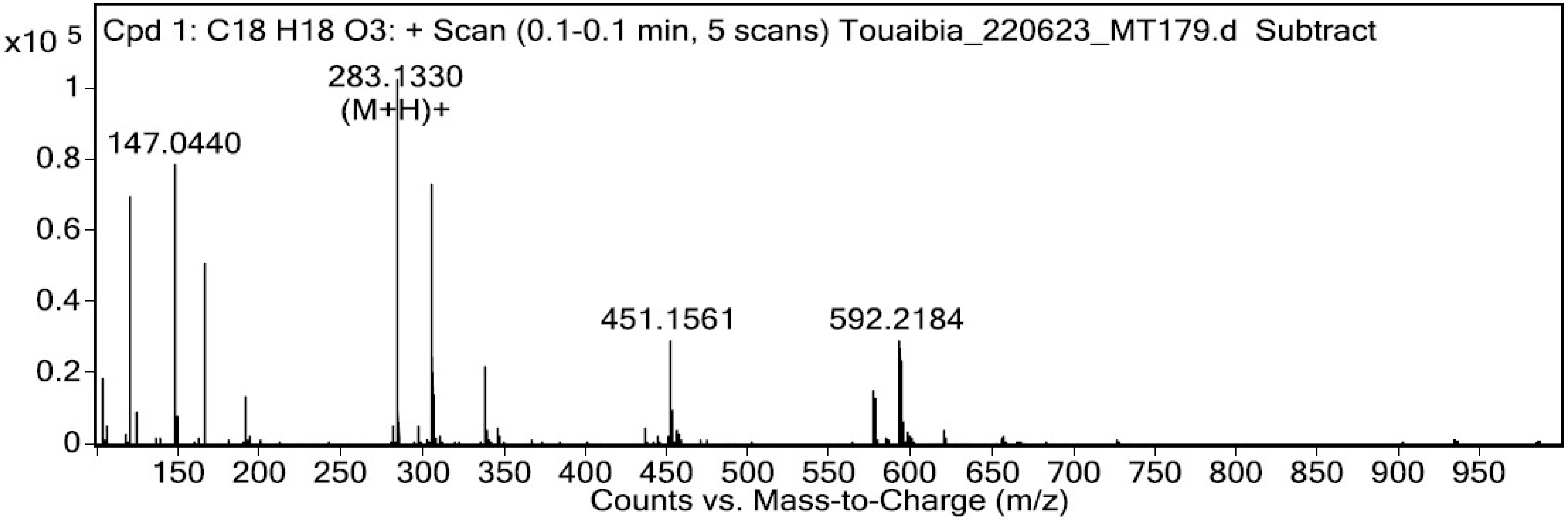

x10 5
Cpd 1: C18 H18 O3: + Scan (0.1-0.1 min, 5 scans) Touaibia_220623_MT179.d Subtract
283.1330
(M+H)+
147.0440
451.1561
592.2184
Counts vs. Mass-to-Charge (m/z)

HPLC

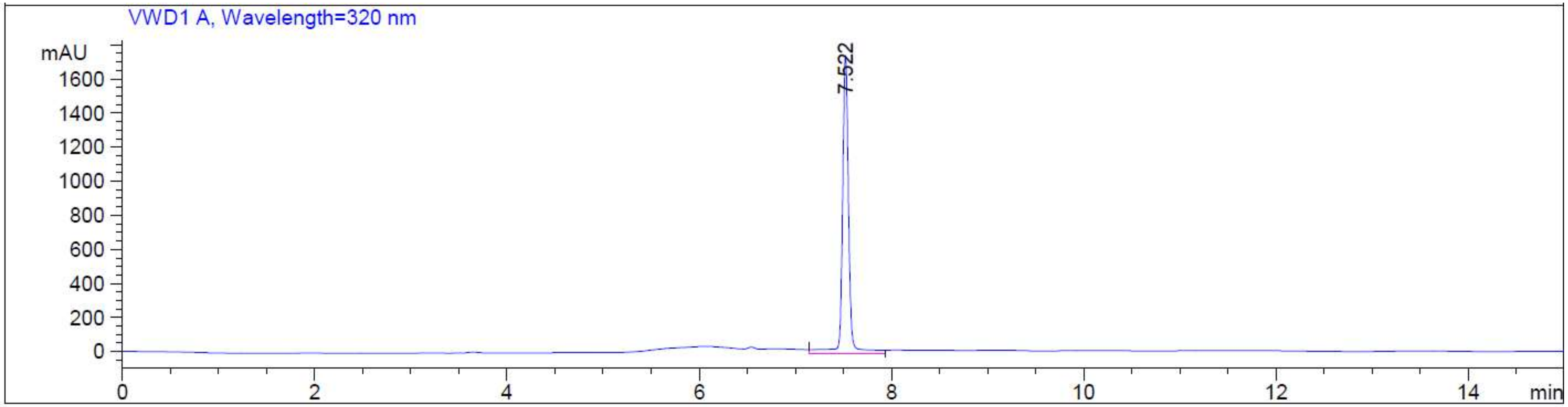

VWD1 A, Wavelength=320 nm
mAU
7.522
min

1H NMR

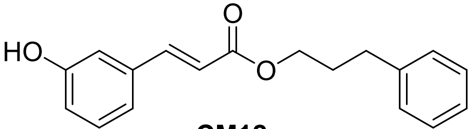

CM12

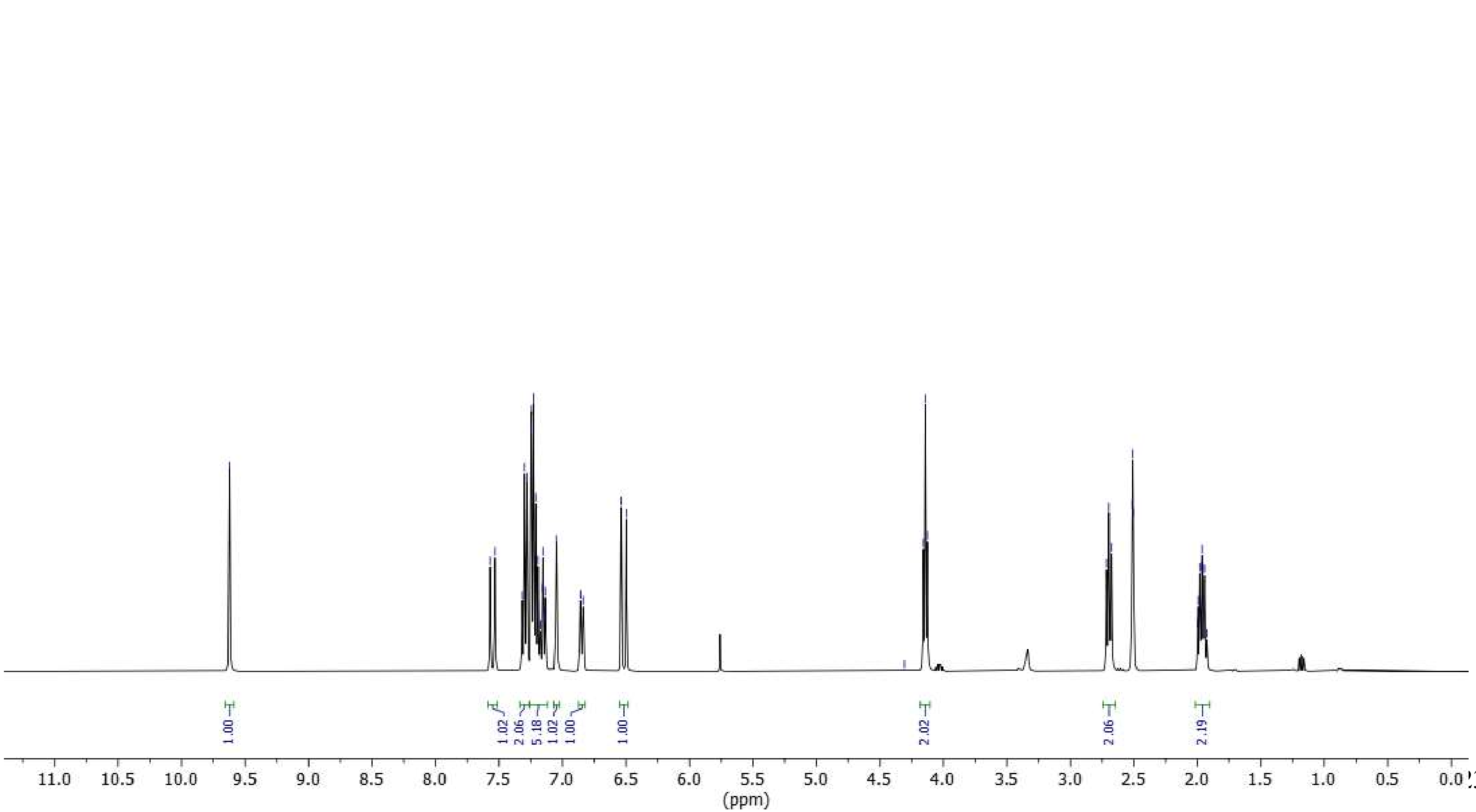

9.62
7.57 7.53 7.32 7.30 7.28 7.25 7.24 7.23 7.21 7.20 7.19 7.17 7.16 7.16 7.15 7.14 7.14 7.13 7.05 6.86 6.84 6.54 6.50
4.31 4.16 4.14 4.13
2.71 2.70 2.68 2.51 2.51 2.50 2.00 1.99 1.98 1.98 1.97 1.96 1.96 1.95 1.94 1.93
1.00 1.02 2.06 5.18 1.02 1.00 1.00 2.02 2.06 2.19
11.0 10.5 10.0 9.5 9.0 8.5 8.0 7.5 7.0 6.5 6.0 5.5 5.0 4.5 4.0 3.5 3.0 2.5 2.0 1.5 1.0 0.5 0.0
(ppm)

13C NMR

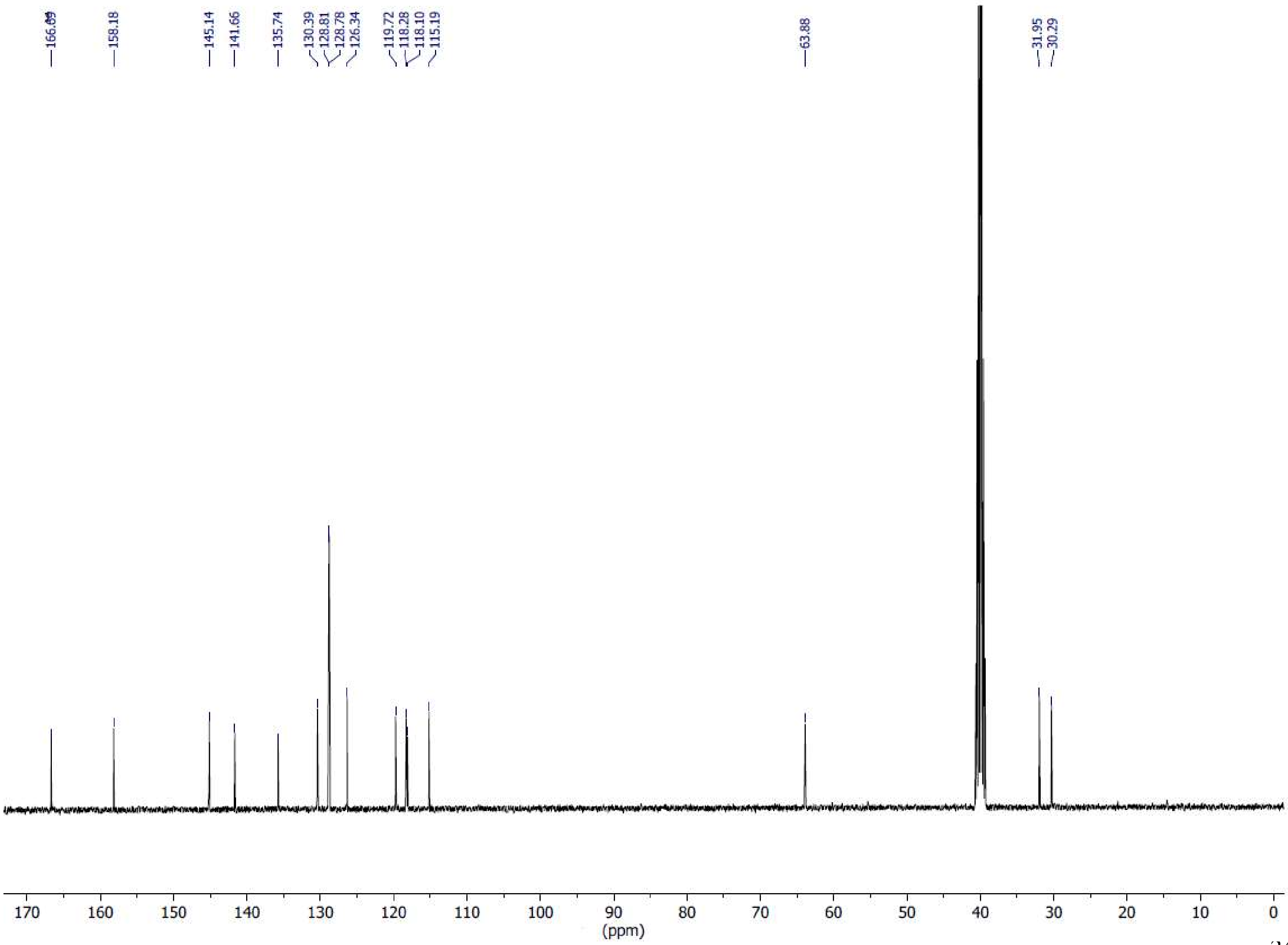

158.18
145.14
141.66
135.74
130.39
128.81
128.78
126.34
119.72
118.28
118.10
115.19
63.88
31.95
30.29
170
160
150
140
130
120
110
100
90
80
70
60
50
40
30
20
10
0
(ppm)

HRMS

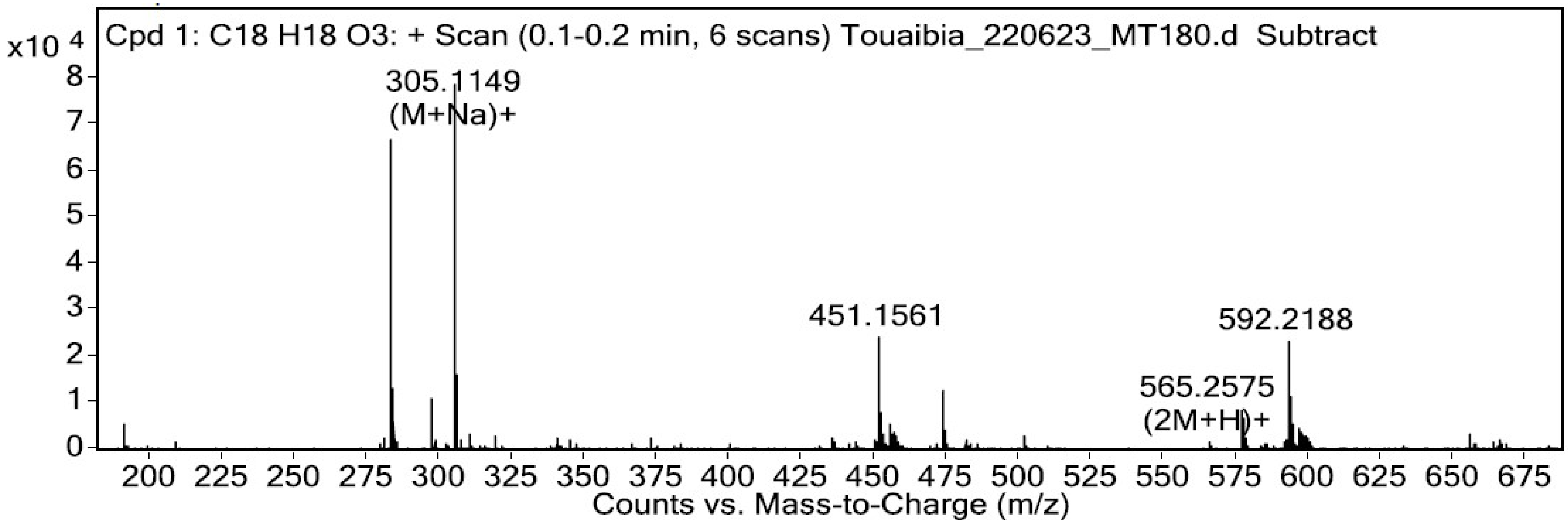

Cpd 1: C18 H18 O3: + Scan (0.1-0.2 min, 6 scans) Touaibia_220623_MT180.d Subtract
x10 4
305.1149
(M+Na)+
451.1561
592.2188
565.2575
(2M+H)+
Counts vs. Mass-to-Charge (m/z)

HPLC

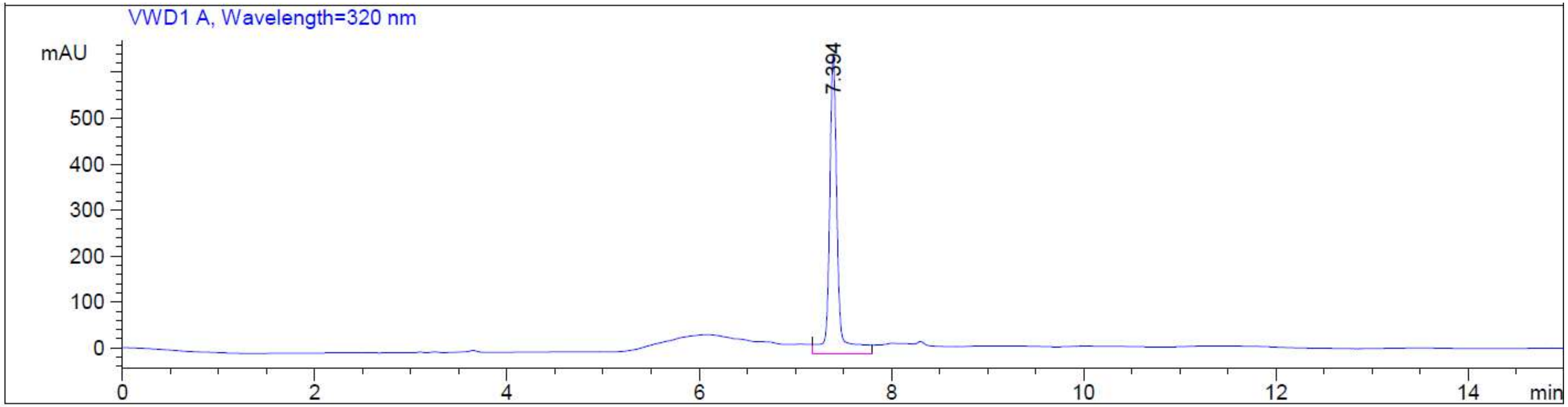

VWD1 A, Wavelength=320 nm
mAU
7.394
min

1H NMR

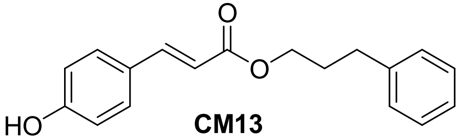

O
O
HO

CM13

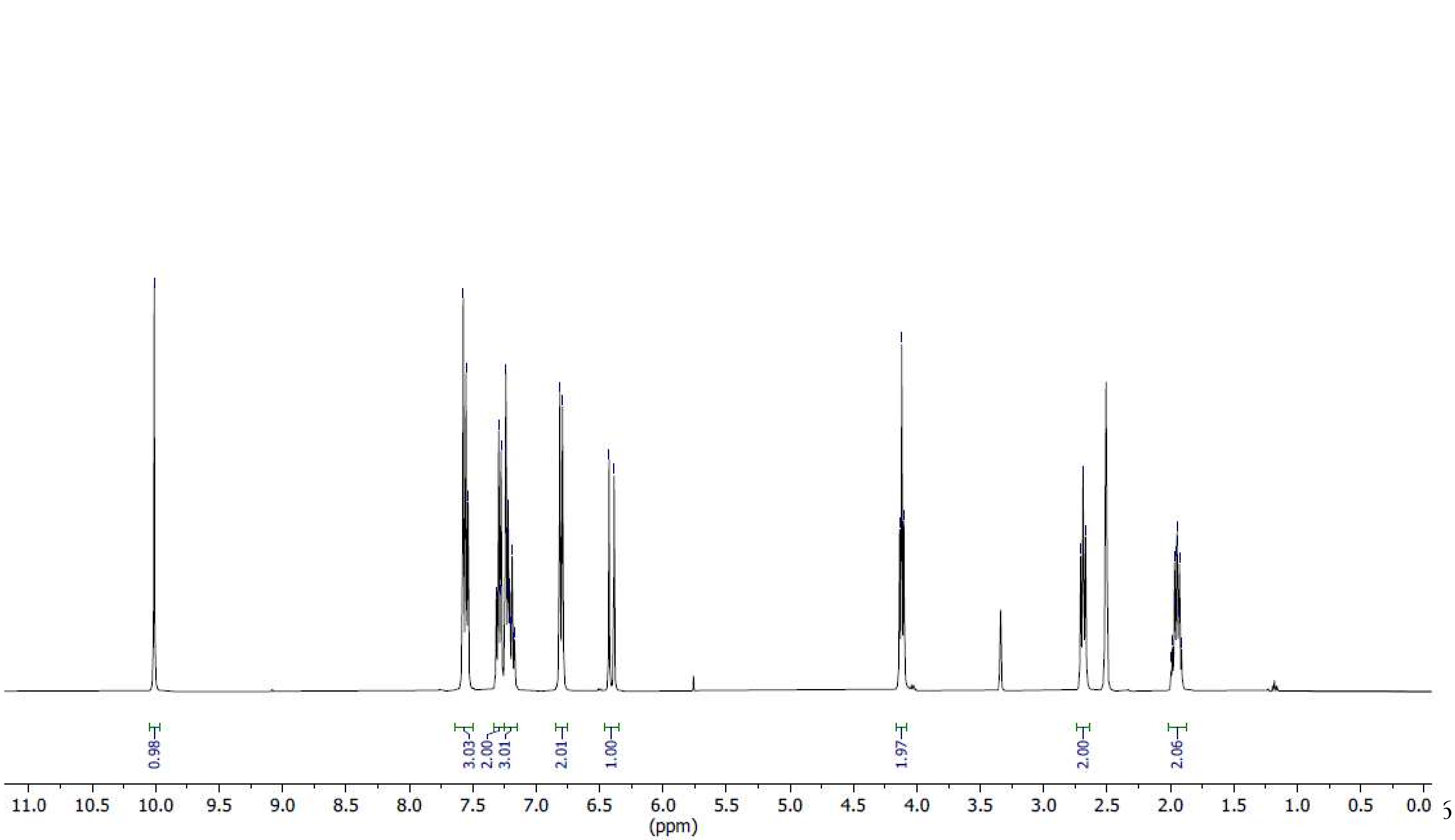

10.01
7.57 7.55 7.54 7.32 7.30 7.28 7.28 7.24 7.22 7.22 7.21 7.21 7.20 7.20 7.19 7.19 7.17 6.81 6.79 6.43 6.39
4.14 4.12 4.10
2.71 2.69 2.67 1.99 1.98 1.97 1.96 1.95 1.95 1.94 1.93 1.91
0.98 3.03 2.00 3.01 2.01 1.00 1.97 2.00 2.06
11.0 10.5 10.0 9.5 9.0 8.5 8.0 7.5 7.0 6.5 6.0 5.5 5.0 4.5 4.0 3.5 3.0 2.5 2.0 1.5 1.0 0.5 0.0
(ppm)

^{13}C NMR

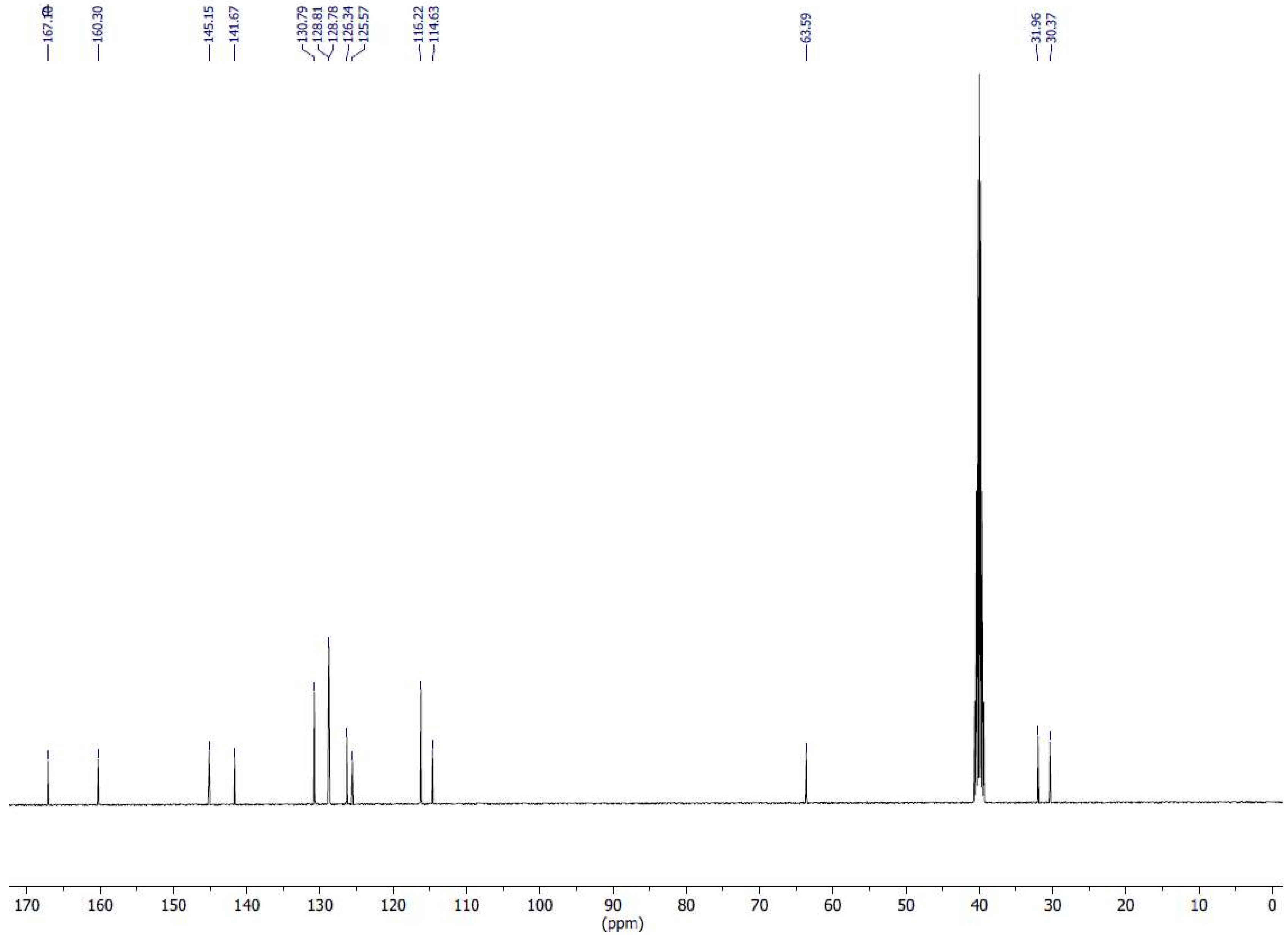
160.30
145.15
141.67
130.79
128.81
128.78
126.34
125.57
116.22
114.63
63.59
31.96
30.37
170 160 150 140 130 120 110 100 90 80 70 60 50 40 30 20 10 0
(ppm)

HRMS

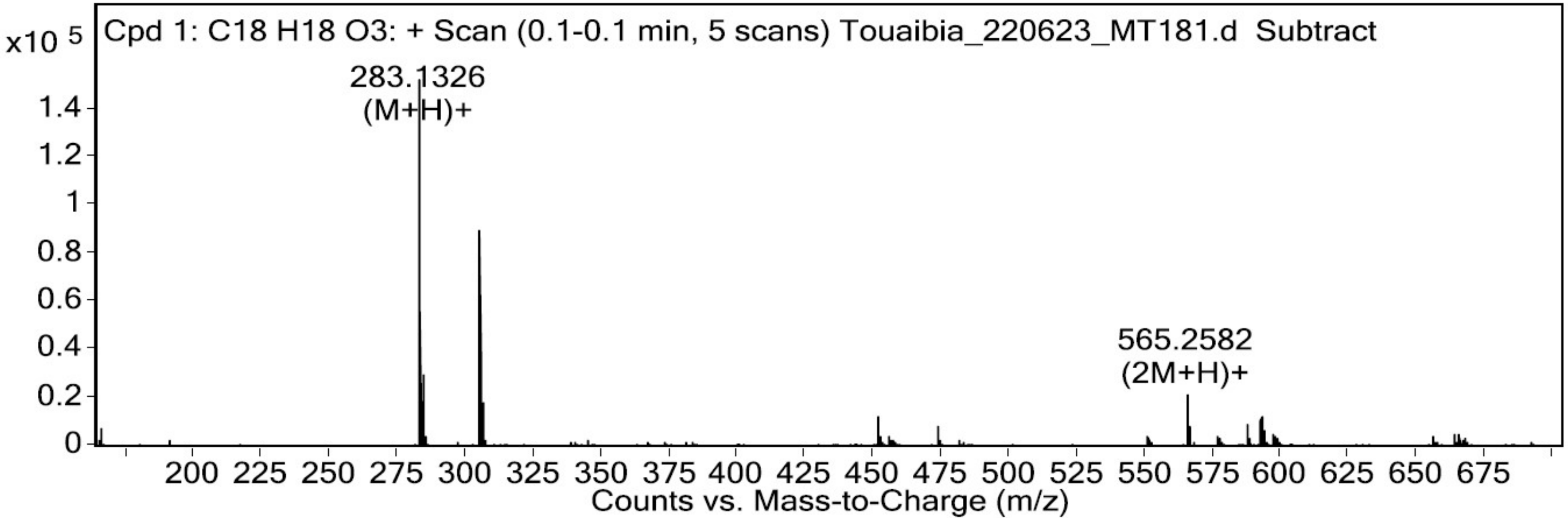

x10 5
Cpd 1: C18 H18 O3: + Scan (0.1-0.1 min, 5 scans) Touaibia_220623_MT181.d Subtract
283.1326
(M+H)+
565.2582
(2M+H)+
1.4
1.2
1
0.8
0.6
0.4
0.2
0
200 225 250 275 300 325 350 375 400 425 450 475 500 525 550 575 600 625 650 675
Counts vs. Mass-to-Charge (m/z)

HPLC

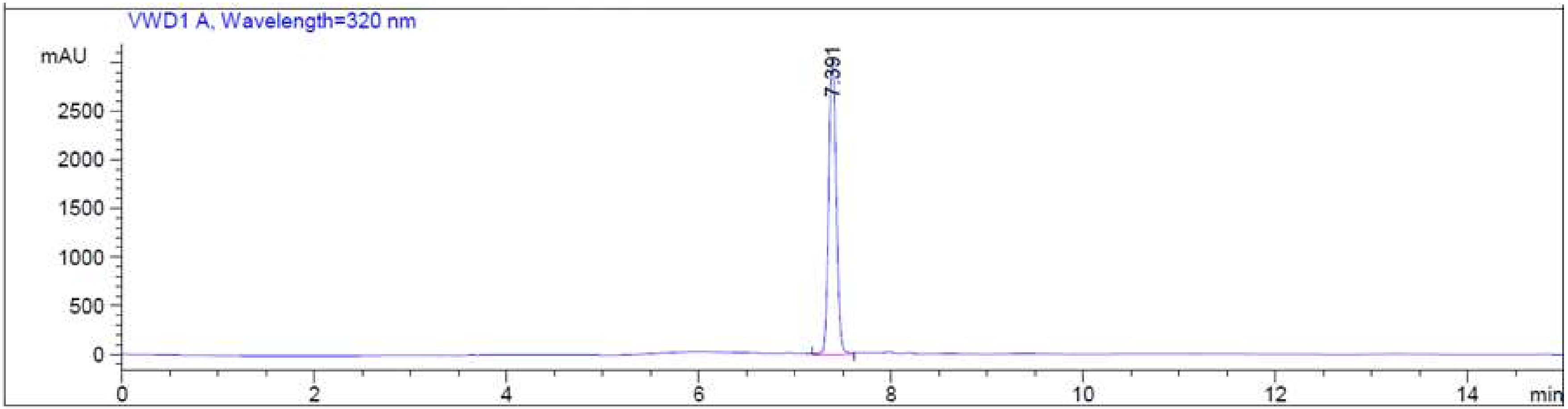

VWD1 A, Wavelength=320 nm
mAU
7.391
2500
2000
1500
1000
500
0
0 2 4 6 8 10 12 14 min

NMR, HRMS, and HPLC of literature unknown ketones analogs

1H NMR

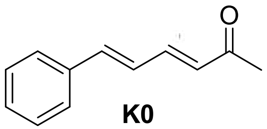

K0

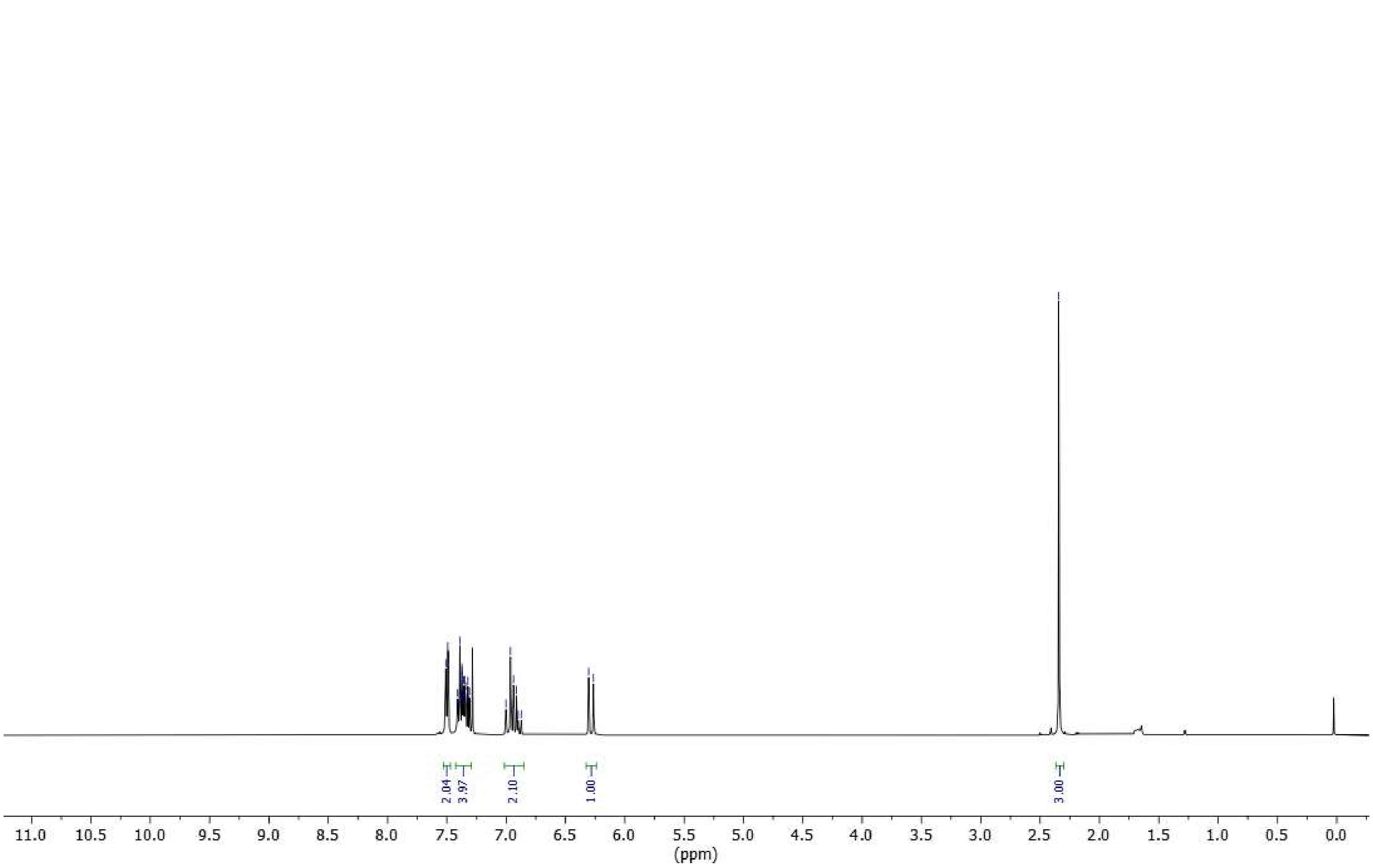

13C NMR

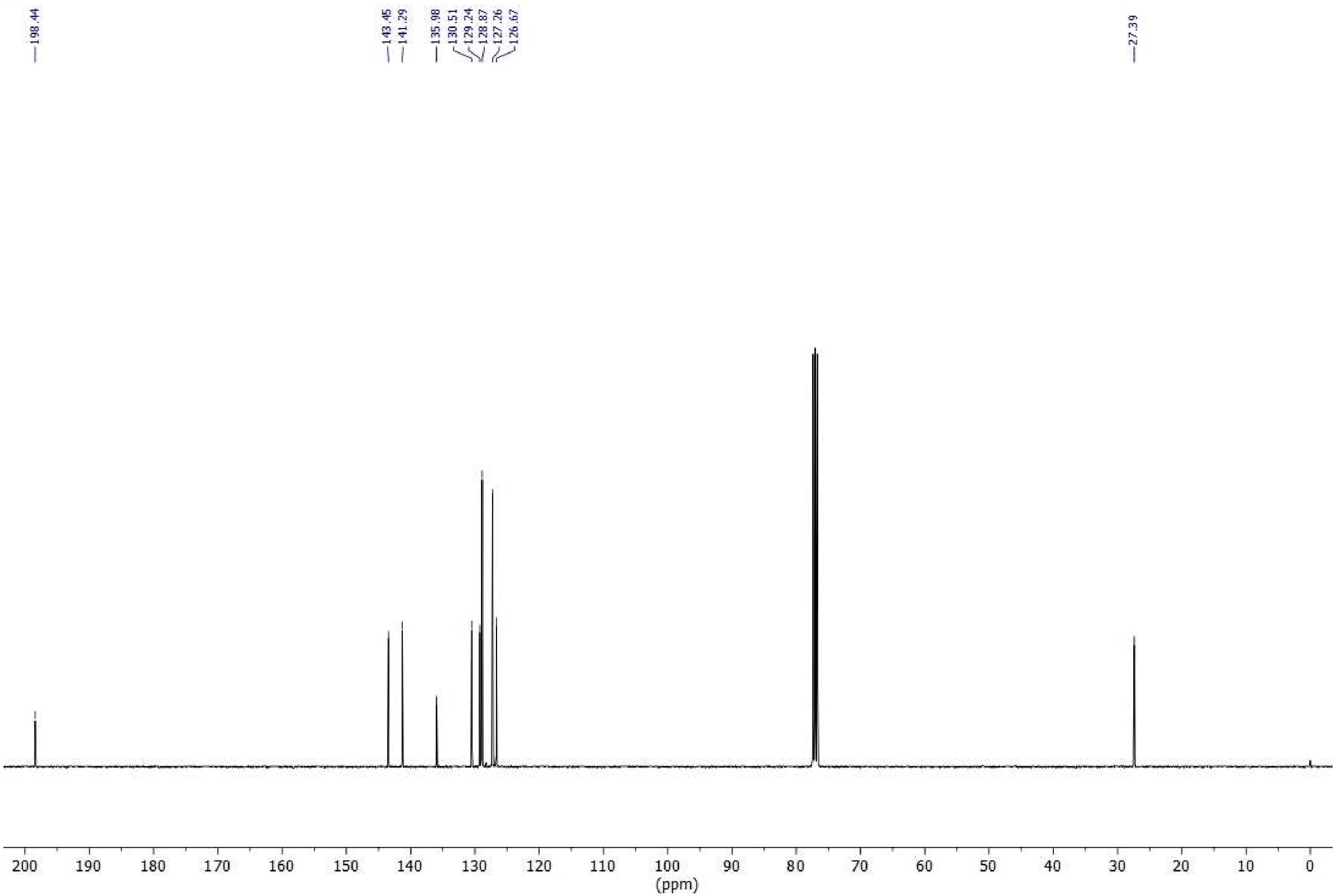
198.44
143.45
141.29
135.98
130.51
129.24
128.87
127.26
126.67
27.39
200 190 180 170 160 150 140 130 120 110 100 90 80 70 60 50 40 30 20 10 0
(ppm)

1H NMR

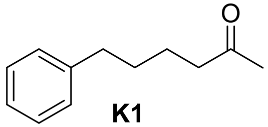

K1

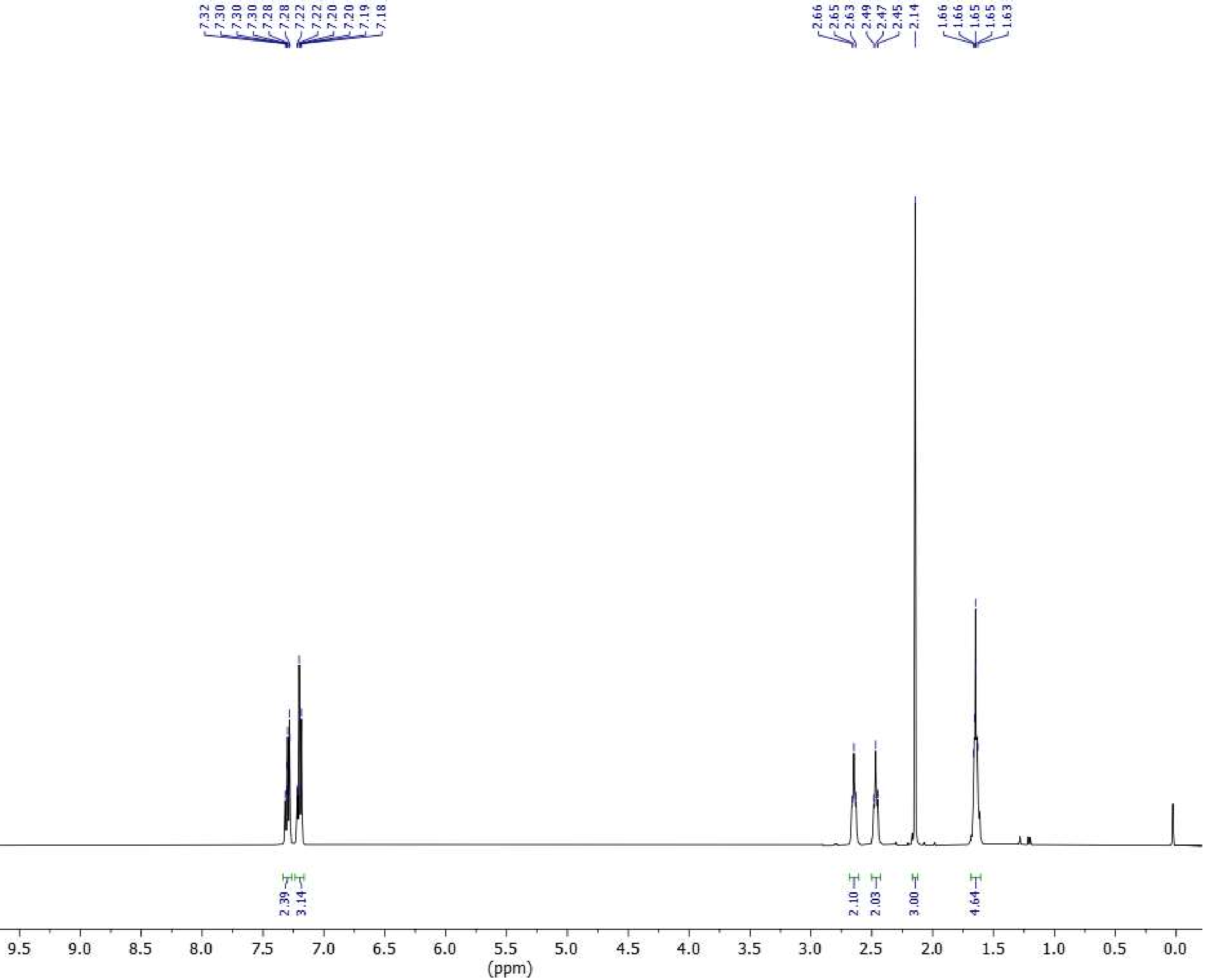

7.32 7.30 7.30 7.30 7.28 7.28 7.22 7.22 7.20 7.20 7.19 7.18
2.66 2.65 2.63 2.49 2.47 2.45 2.14 1.66 1.66 1.65 1.65 1.63
2.39 3.14 2.10 2.03 3.00 4.64
11.0 10.5 10.0 9.5 9.0 8.5 8.0 7.5 7.0 6.5 6.0 5.5 5.0 4.5 4.0 3.5 3.0 2.5 2.0 1.5 1.0 0.5 0.0
(ppm)

13C NMR

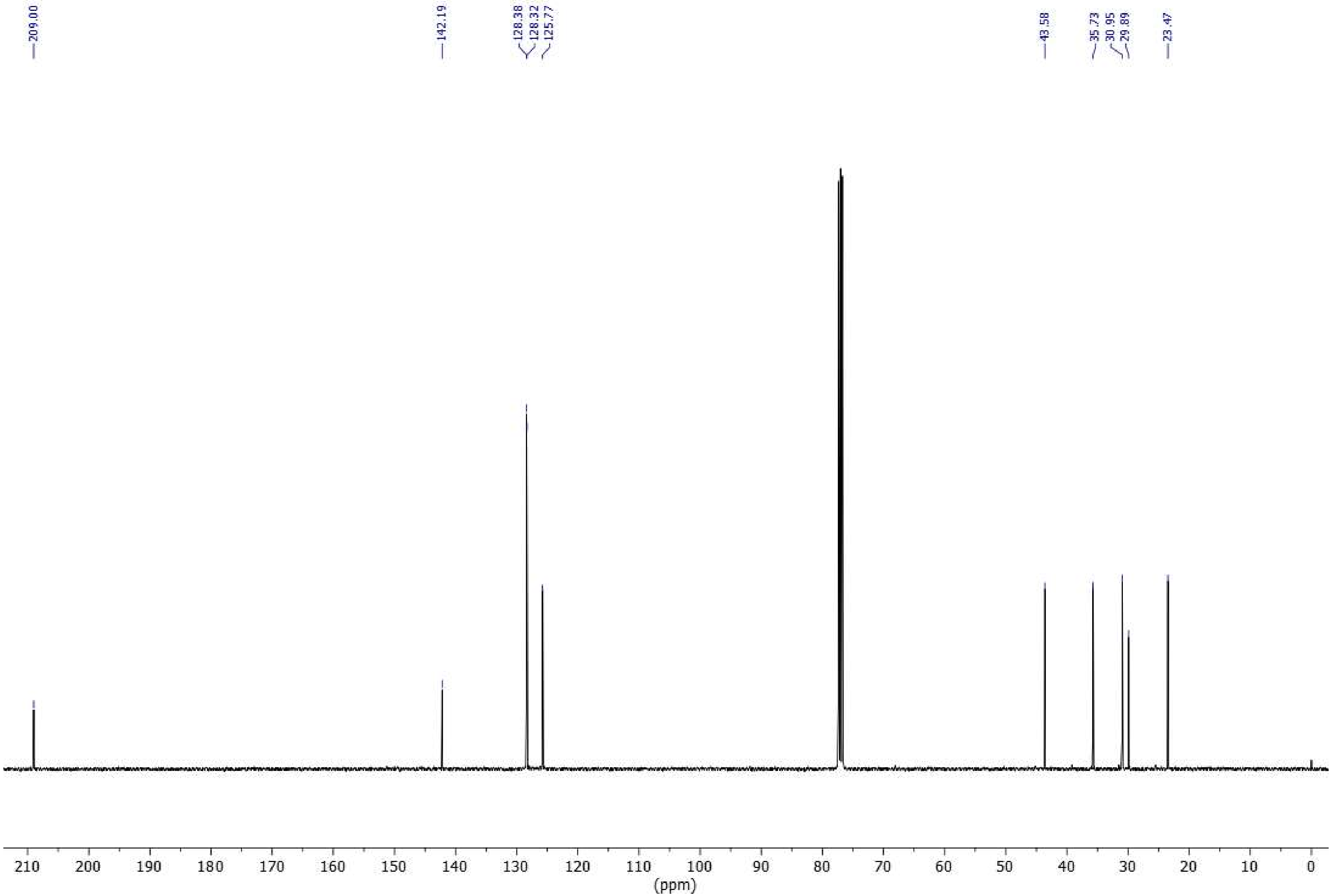

209.00
142.19
128.38
128.32
125.77
43.58
35.73
30.95
29.89
23.47
210
200
190
180
170
160
150
140
130
120
110
100
90
80
70
60
50
40
30
20
10
0
(ppm)

1H NMR

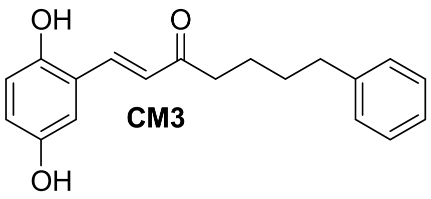
OH
O
CM3
OH

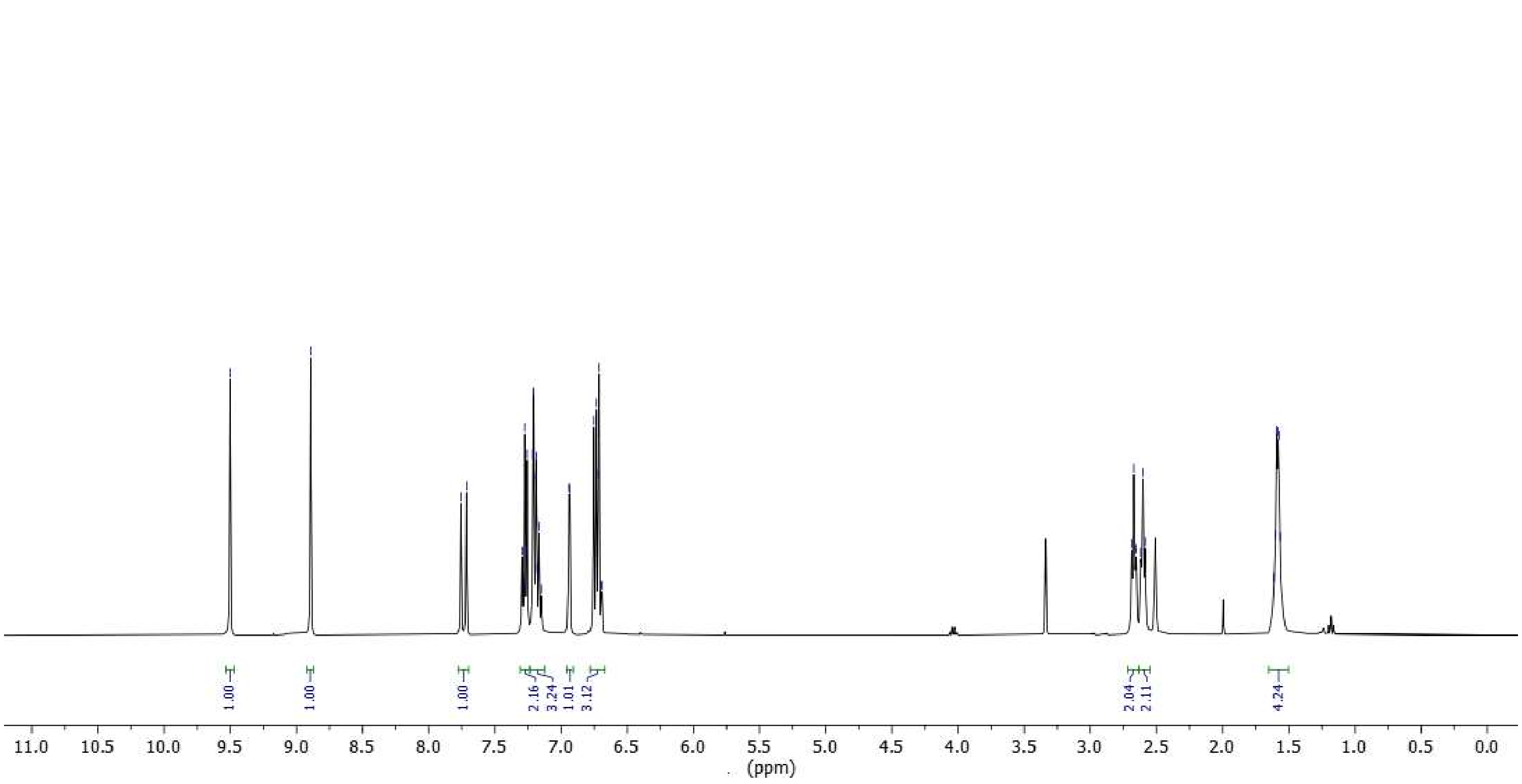
9.50
8.89
7.76
7.72
7.29
7.29
7.28
7.26
7.26
7.21
7.21
7.19
7.19
7.18
7.18
7.17
7.17
7.15
6.94
6.93
6.75
6.73
6.72
6.71
6.70
6.69
2.69
2.67
2.65
2.62
2.60
2.58
1.61
1.60
1.59
1.59
1.58
1.57
1.57
1.00
1.00
1.00
2.16
3.24
1.01
3.12
2.04
2.11
4.24
11.0 10.5 10.0 9.5 9.0 8.5 8.0 7.5 7.0 6.5 6.0 5.5 5.0 4.5 4.0 3.5 3.0 2.5 2.0 1.5 1.0 0.5 0.0
(ppm)

13C NMR

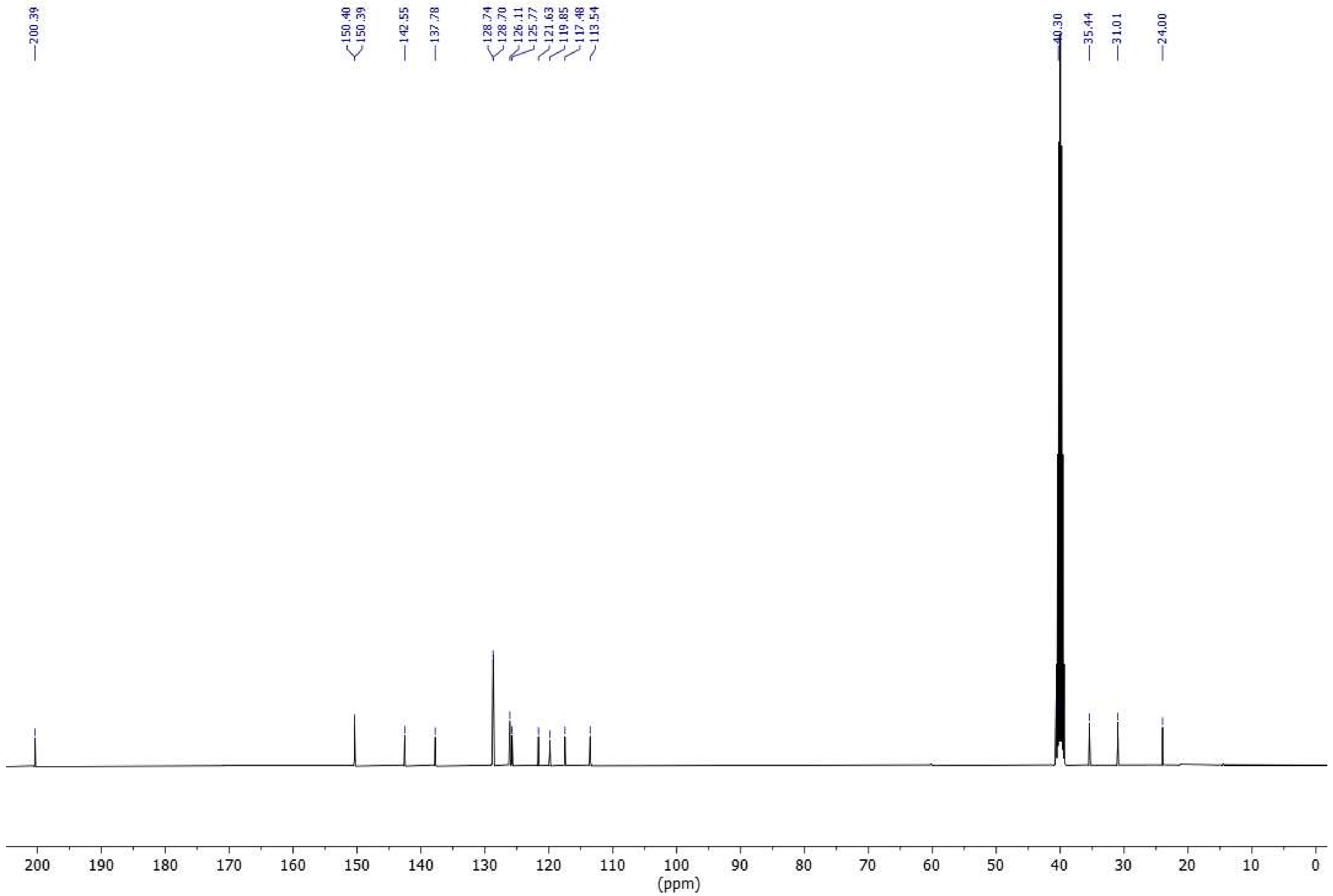

200.39
150.40
150.39
142.55
137.78
128.74
128.70
126.11
125.77
121.63
119.85
117.48
113.54
40.30
35.44
31.01
24.00
200 190 180 170 160 150 140 130 120 110 100 90 80 70 60 50 40 30 20 10 0
(ppm)

HRMS

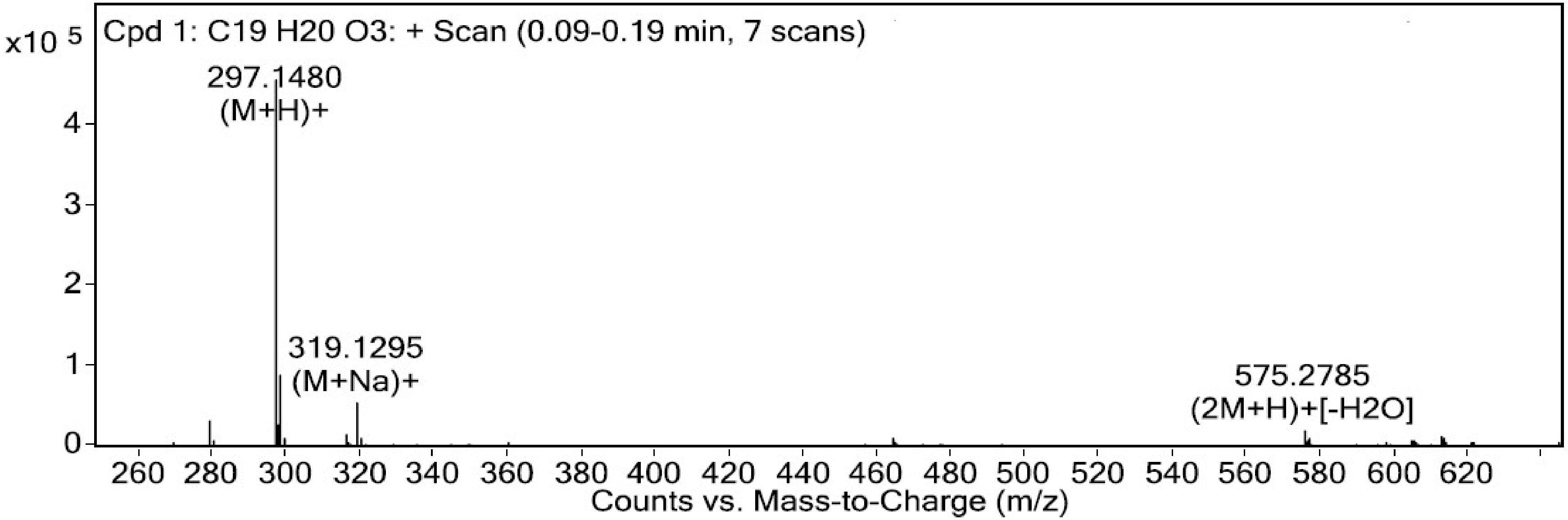

Cpd 1: C19 H20 O3: + Scan (0.09-0.19 min, 7 scans)
x10 5
297.1480
(M+H)+
319.1295
(M+Na)+
575.2785
(2M+H)+[-H2O]
Counts vs. Mass-to-Charge (m/z)

HPLC

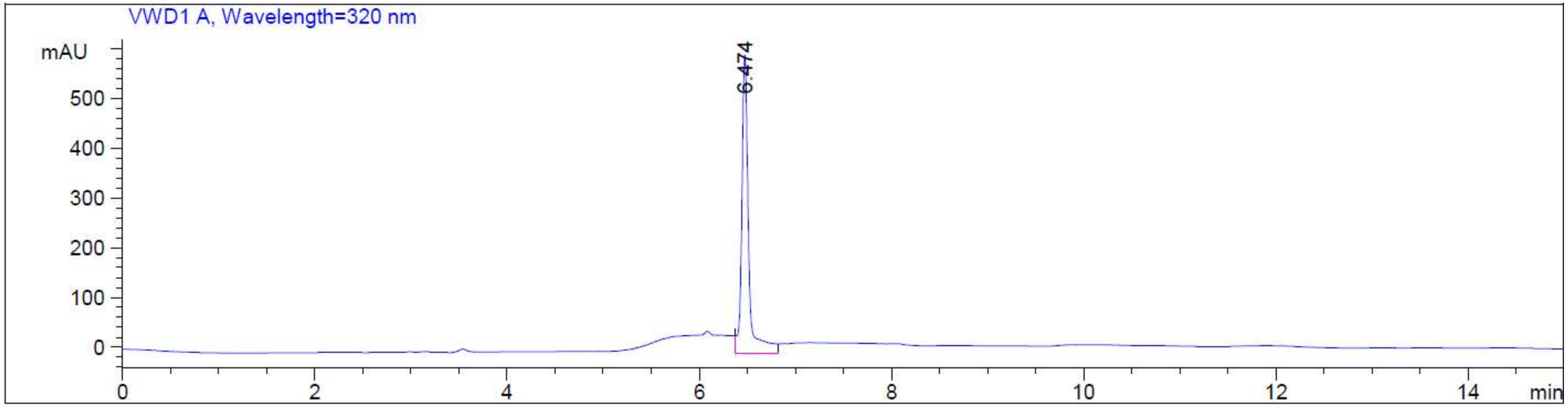

VWD1 A, Wavelength=320 nm
mAU
6.474
min

1H NMR

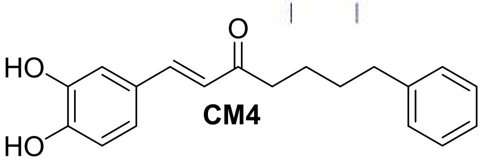

HO
HO
O

CM4

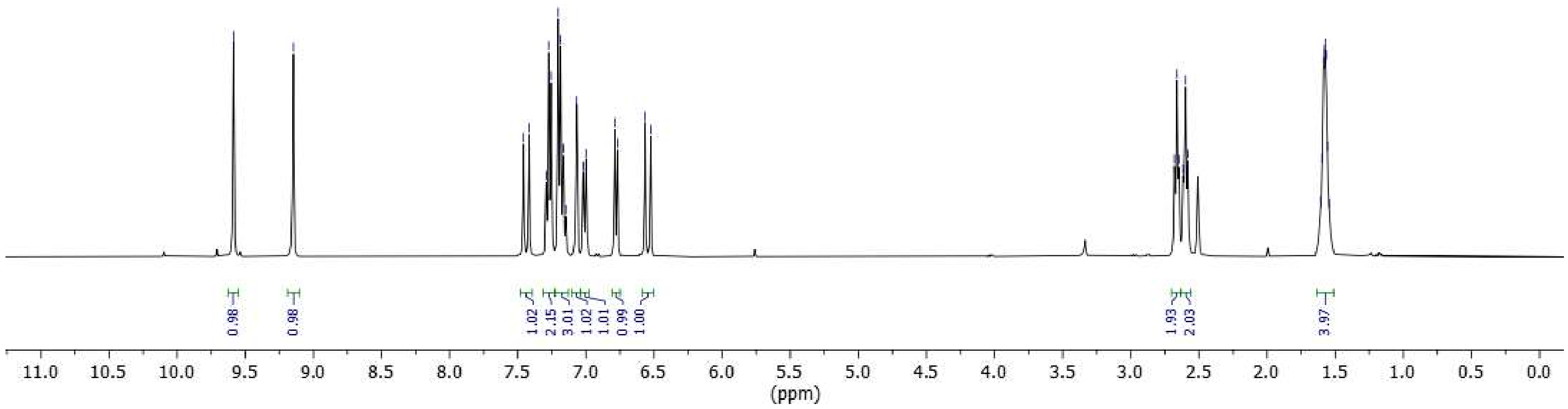

9.59
9.15
7.46 7.42 7.29 7.27 7.26 7.20 7.19 7.17 7.15 7.07 7.02 7.00 6.79 6.77 6.57 6.52
2.68 2.66 2.65 2.62 2.60 2.58
1.61 1.60 1.58 1.58 1.57 1.56 1.54
0.98 0.98 1.02 2.15 3.01 1.02 1.01 0.99 1.00 1.93 2.03 3.97
11.0 10.5 10.0 9.5 9.0 8.5 8.0 7.5 7.0 6.5 6.0 5.5 5.0 4.5 4.0 3.5 3.0 2.5 2.0 1.5 1.0 0.5 0.0
(ppm)

13C NMR

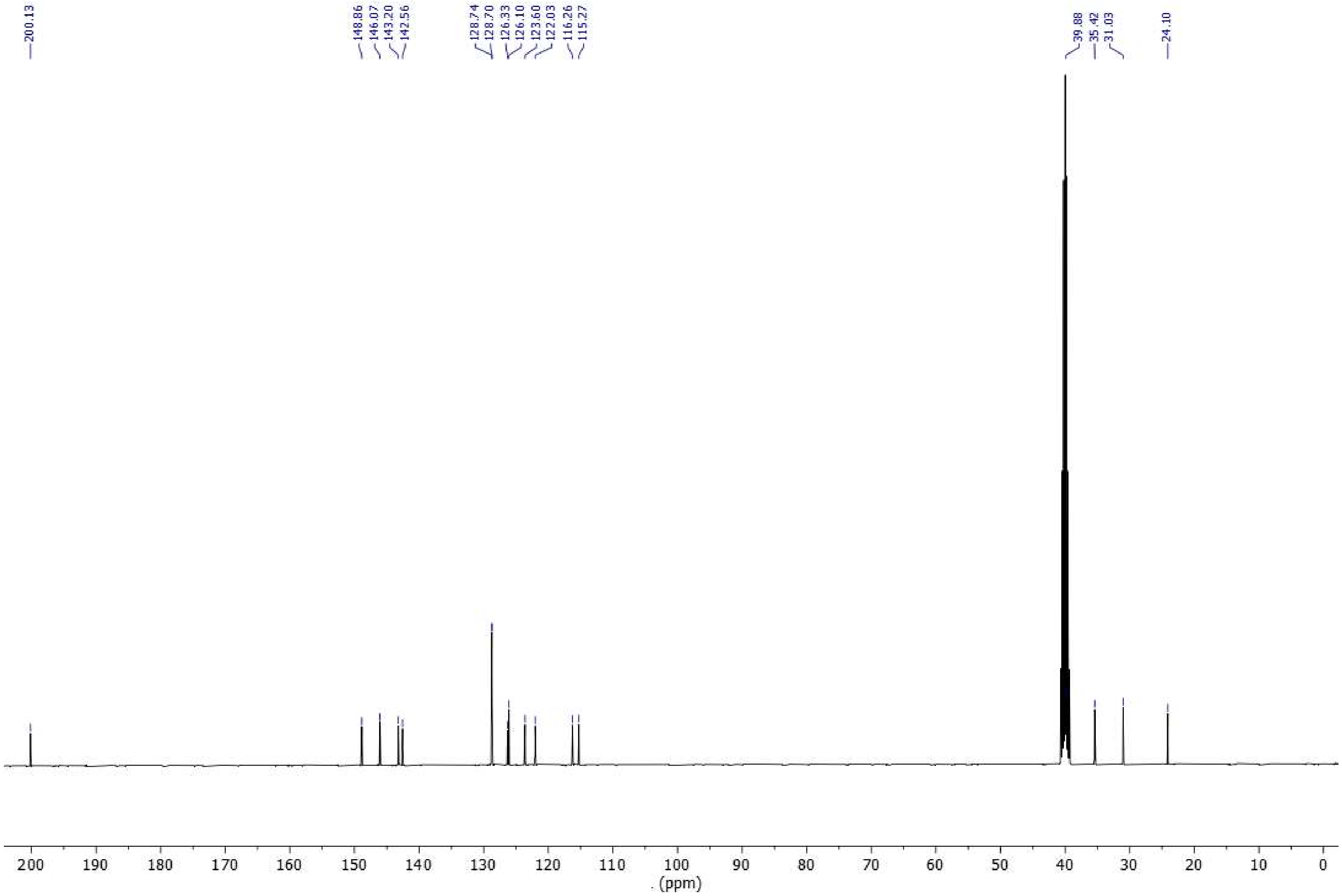

200.13
148.86
146.07
143.20
142.56
128.74
128.70
126.33
126.10
123.60
122.03
116.26
115.27
39.88
35.42
31.03
24.10
200
190
180
170
160
150
140
130
120
110
100
90
80
70
60
50
40
30
20
10
0
. (ppm)

HRMS

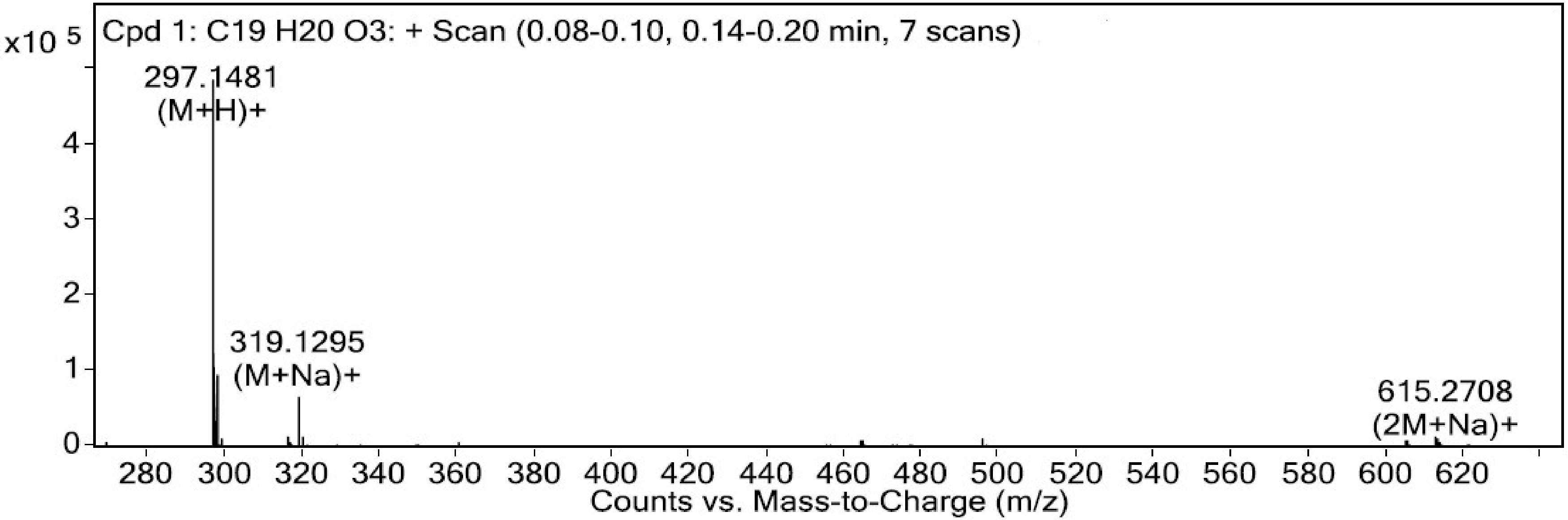

x10 5
Cpd 1: C19 H20 O3: + Scan (0.08-0.10, 0.14-0.20 min, 7 scans)
297.1481
(M+H)+
319.1295
(M+Na)+
615.2708
(2M+Na)+
0
1
2
3
4
280 300 320 340 360 380 400 420 440 460 480 500 520 540 560 580 600 620
Counts vs. Mass-to-Charge (m/z)

HPLC

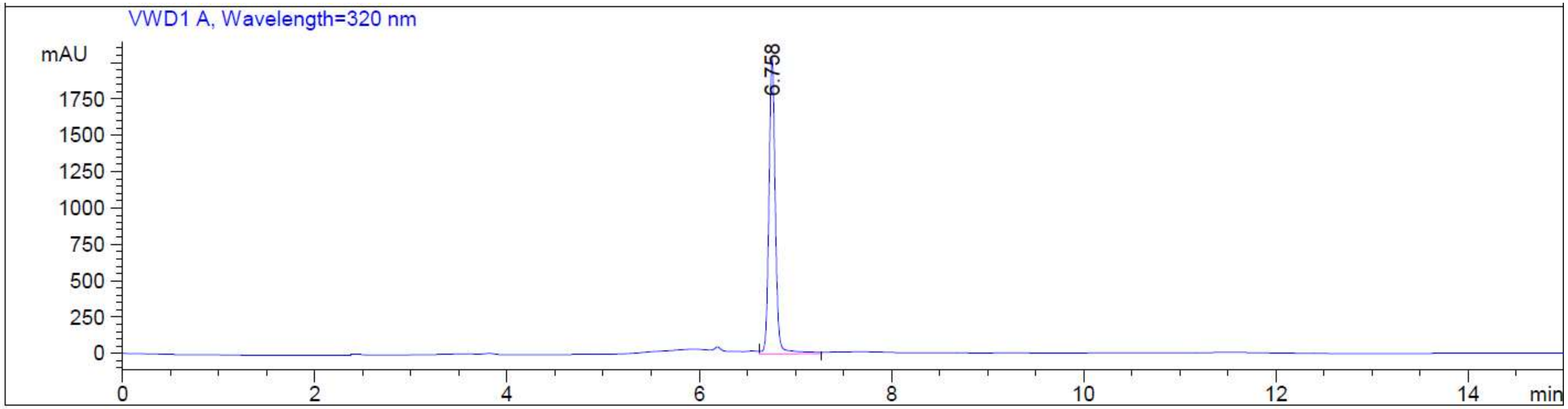

VWD1 A, Wavelength=320 nm
mAU
1750
1500
1250
1000
750
500
250
0
6.758
0
2
4
6
8
10
12
14
min

1H NMR

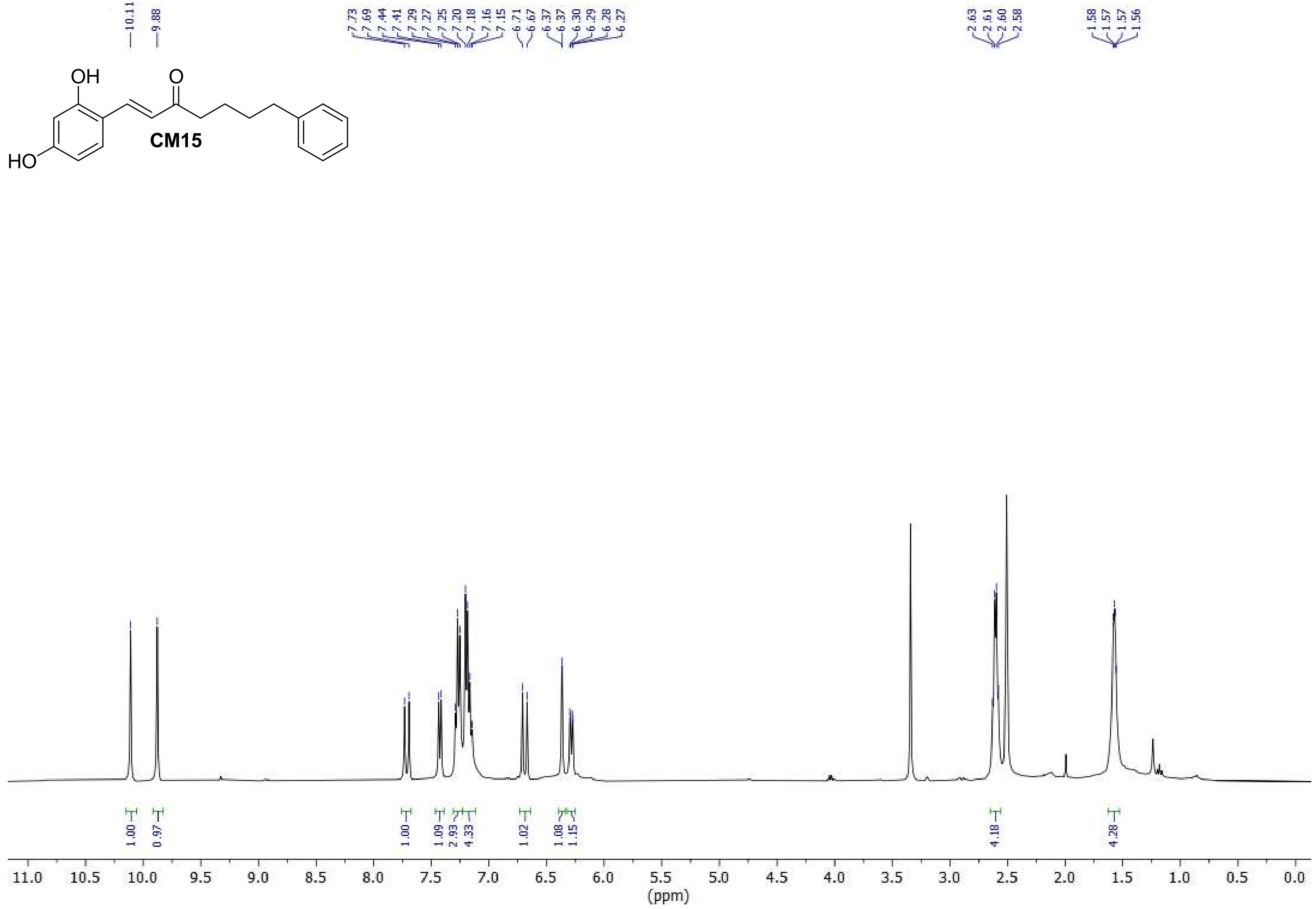

10.11
9.88
7.73 7.69 7.44 7.41 7.29 7.27 7.25 7.20 7.18 7.16 7.15 6.71 6.67 6.37 6.37 6.30 6.29 6.28 6.27
2.63 2.61 2.60 2.58
1.58 1.57 1.57 1.56
OH
O
HO
CM15
1.00
0.97
1.00
1.09
2.93
4.33
1.02
1.08
1.15
4.18
4.28
11.0 10.5 10.0 9.5 9.0 8.5 8.0 7.5 7.0 6.5 6.0 5.5 5.0 4.5 4.0 3.5 3.0 2.5 2.0 1.5 1.0 0.5 0.0
(ppm)

13C NMR

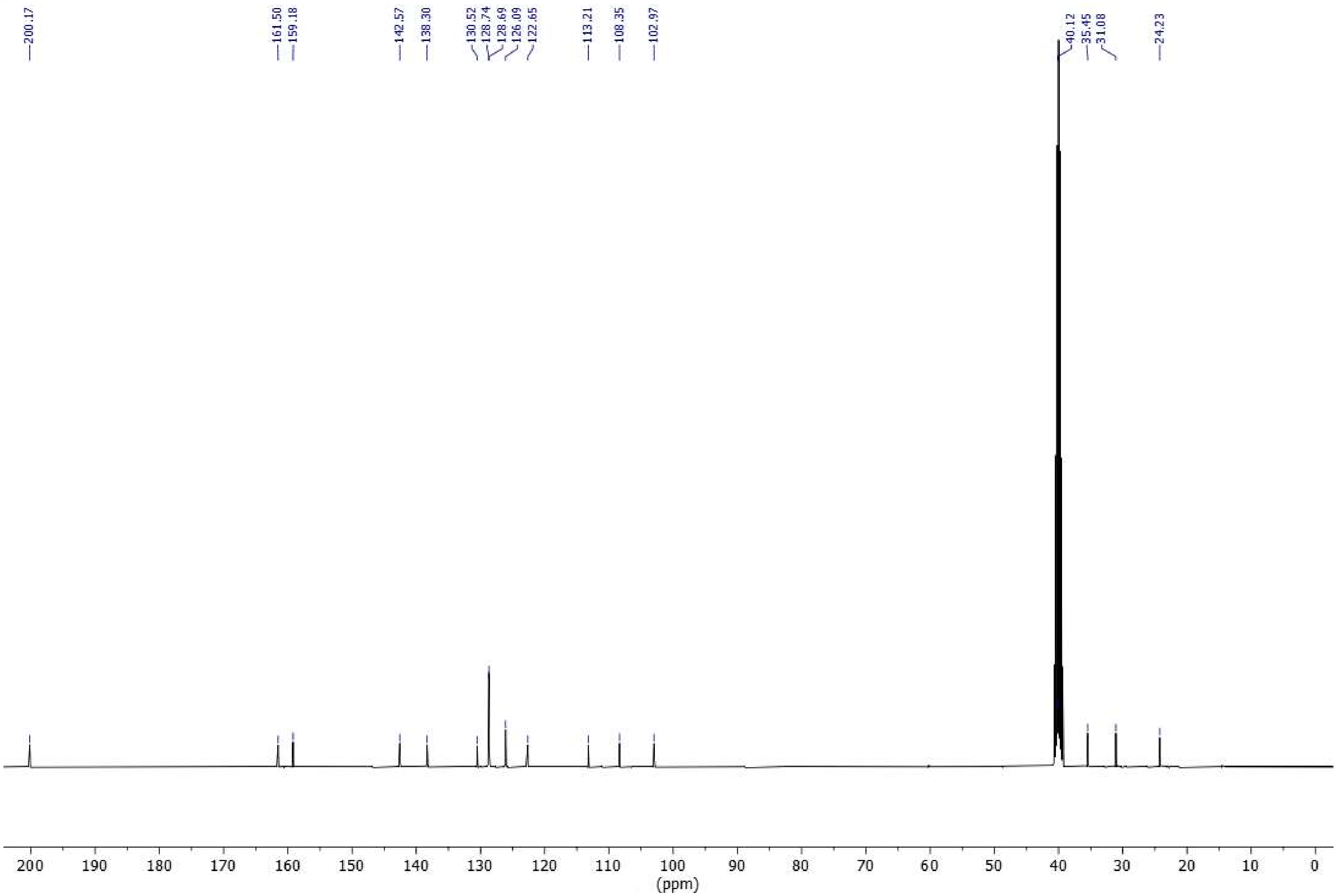

200.17
161.50
159.18
142.57
138.30
130.52
128.74
128.69
126.09
122.65
113.21
108.35
102.97
40.12
35.45
31.08
24.23
200
190
180
170
160
150
140
130
120
110
100
90
80
70
60
50
40
30
20
10
0
(ppm)

HRMS

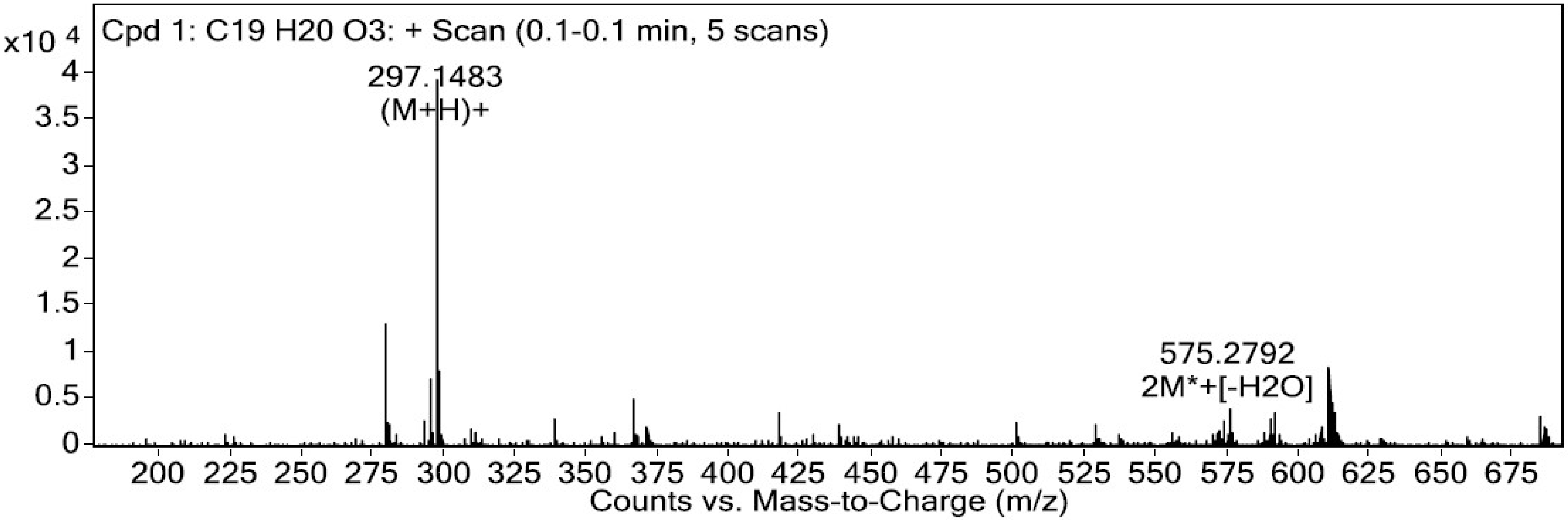

Cpd 1: C19 H20 O3: + Scan (0.1-0.1 min, 5 scans)
x10 4
297.1483
(M+H)+
575.2792
2M*+[-H2O]
Counts vs. Mass-to-Charge (m/z)

HPLC

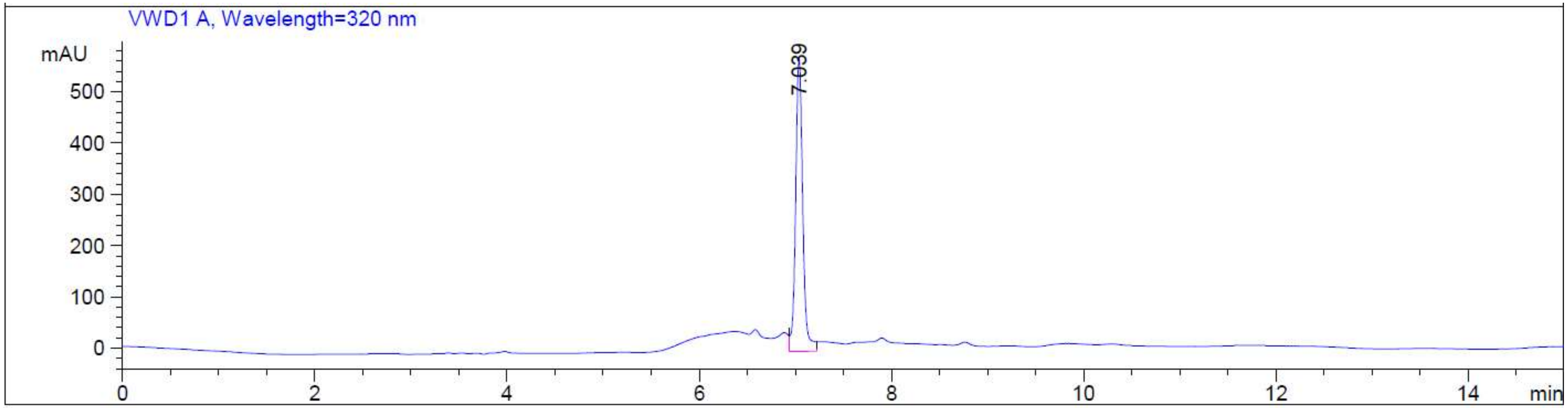

VWD1 A, Wavelength=320 nm
mAU
7.039
min

1H NMR

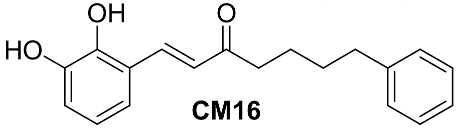
OH
O
HO
CM16

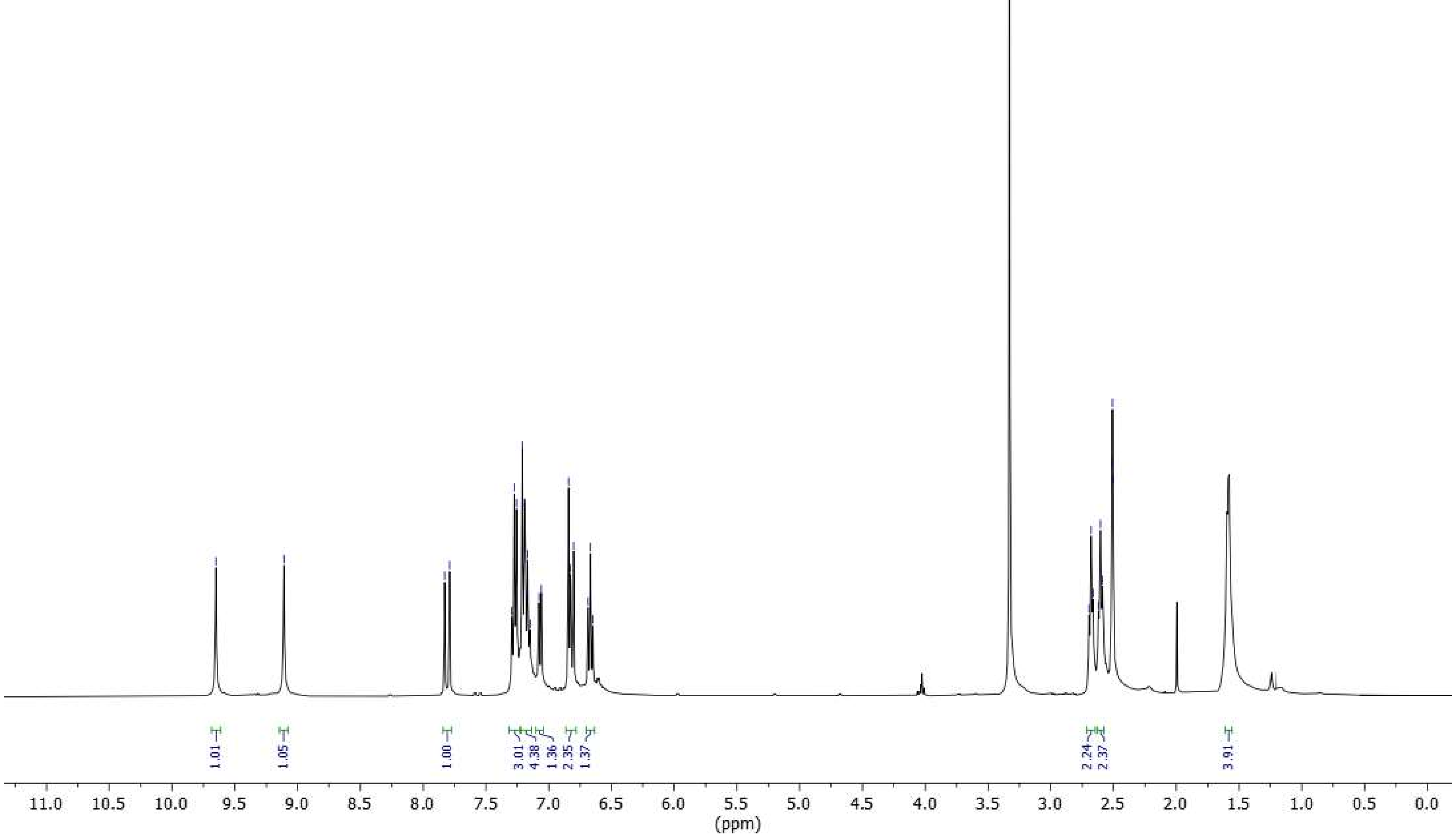
9.65
9.11
7.83
7.79
7.29
7.27
7.26
7.21
7.19
7.19
7.17
7.17
7.15
7.08
7.06
6.84
6.83
6.80
6.69
6.67
6.65
2.70
2.68
2.66
2.62
2.60
2.59
2.51
2.51
2.50
1.01
1.05
1.00
3.01
4.38
1.36
2.35
1.37
2.24
2.37
3.91
11.0 10.5 10.0 9.5 9.0 8.5 8.0 7.5 7.0 6.5 6.0 5.5 5.0 4.5 4.0 3.5 3.0 2.5 2.0 1.5 1.0 0.5 0.0
(ppm)

13C NMR

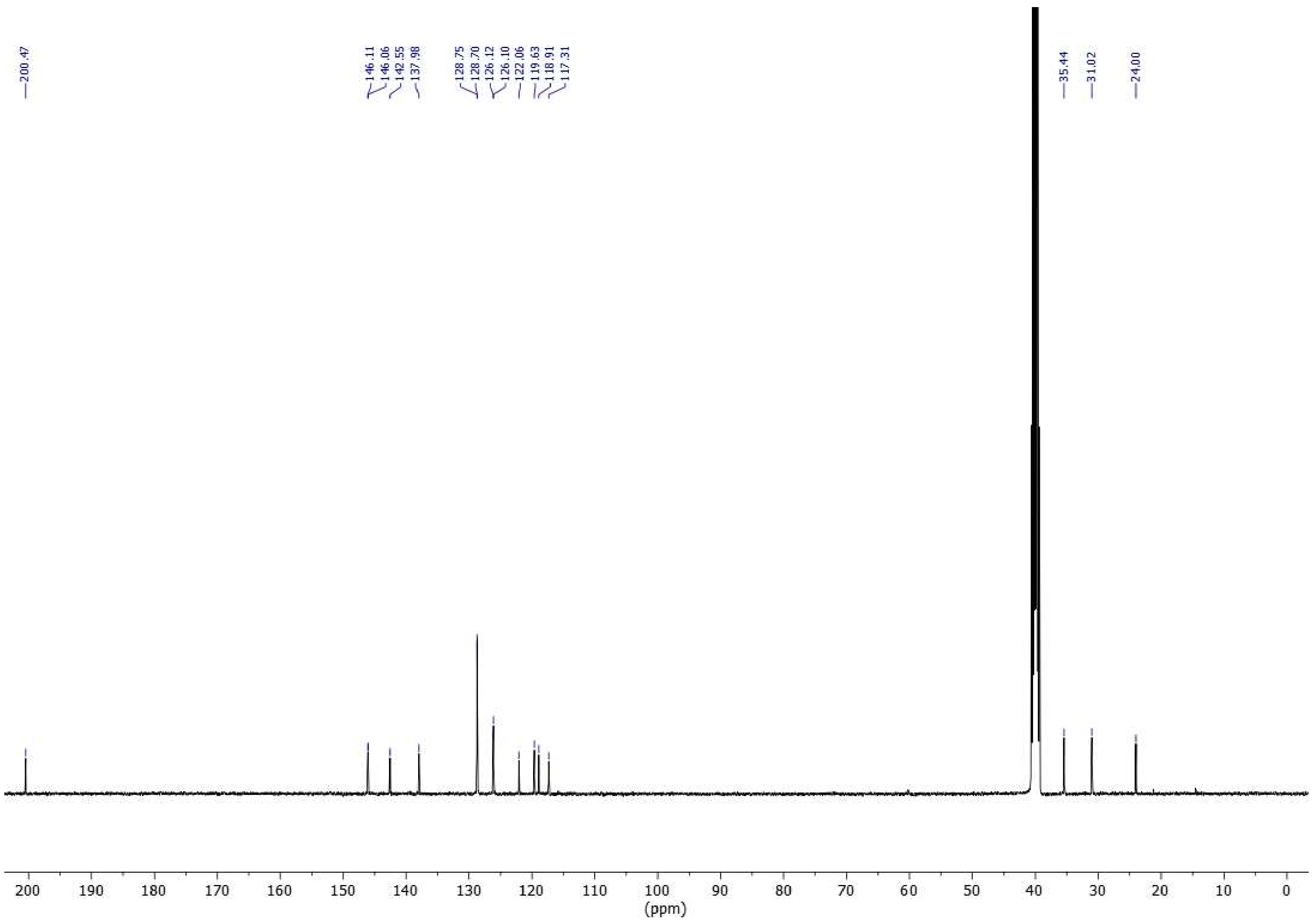
200.47
146.11
146.06
142.55
137.98
128.75
128.70
126.12
126.10
122.06
119.63
118.91
117.31
35.44
31.02
24.00
200
190
180
170
160
150
140
130
120
110
100
(ppm)
90
80
70
60
50
40
30
20
10
0

HRMS

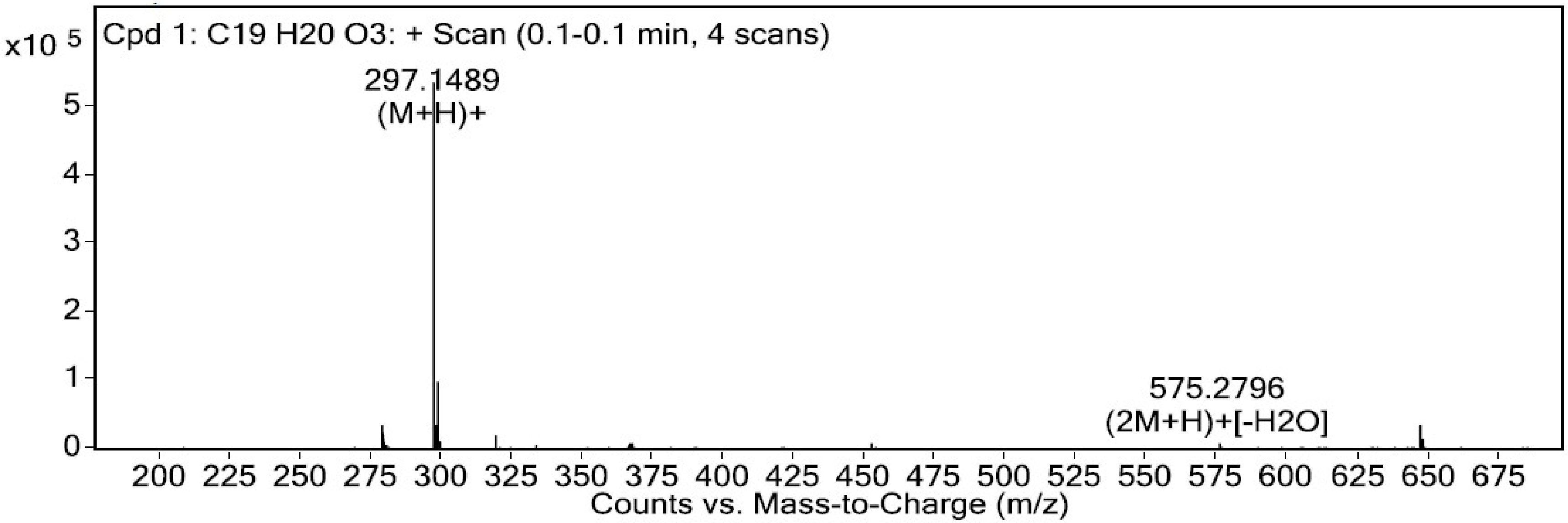

x10 5
Cpd 1: C19 H20 O3: + Scan (0.1-0.1 min, 4 scans)
297.1489
(M+H)+
575.2796
(2M+H)+[-H2O]
Counts vs. Mass-to-Charge (m/z)

HPLC

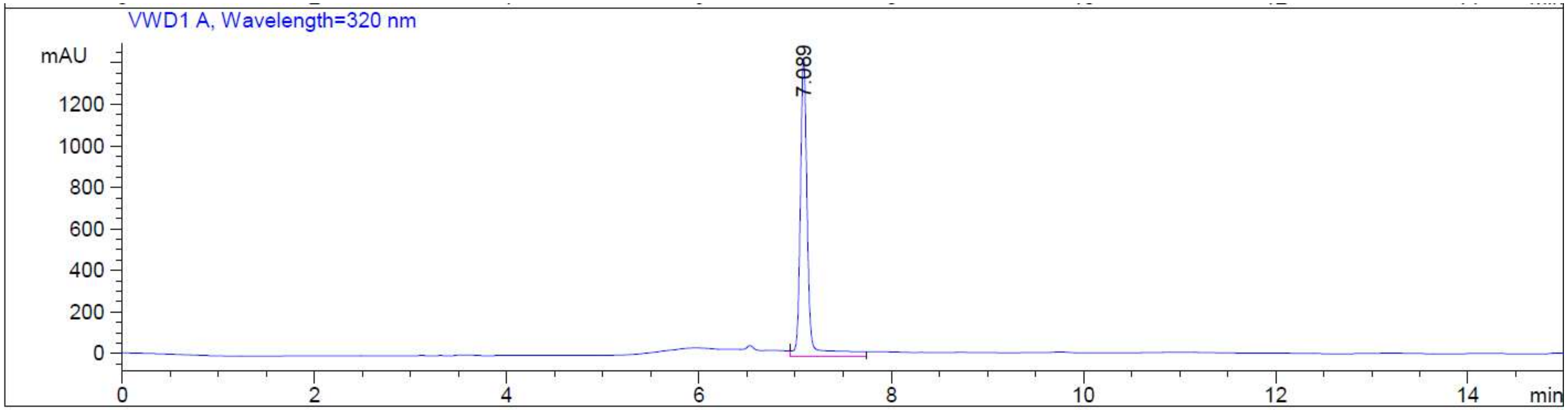

VWD1 A, Wavelength=320 nm
mAU
7.089
min

1H NMR

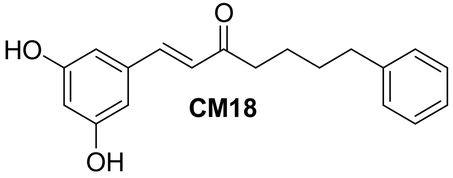
HO
OH
O

CM18

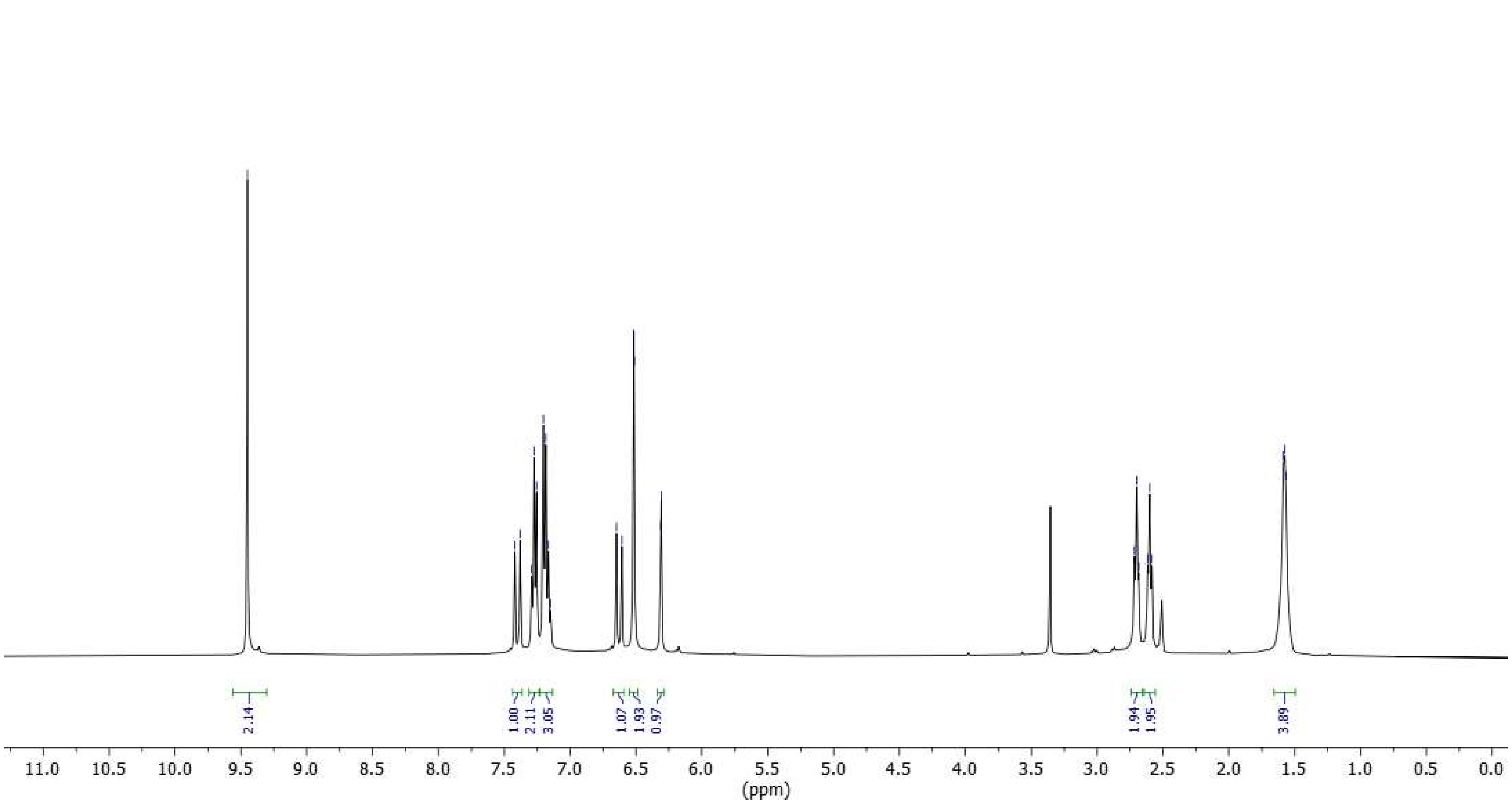
9.45
7.42
7.38
7.29
7.27
7.26
7.21
7.19
7.17
7.15
6.65
6.61
6.52
6.51
6.31
6.31
2.72
2.70
2.68
2.62
2.60
2.58
1.58
1.58
1.57
2.14
1.00
2.11
3.05
1.07
1.93
0.97
1.94
1.95
3.89
11.0 10.5 10.0 9.5 9.0 8.5 8.0 7.5 7.0 6.5 6.0 5.5 5.0 4.5 4.0 3.5 3.0 2.5 2.0 1.5 1.0 0.5 0.0
(ppm)

13C NMR

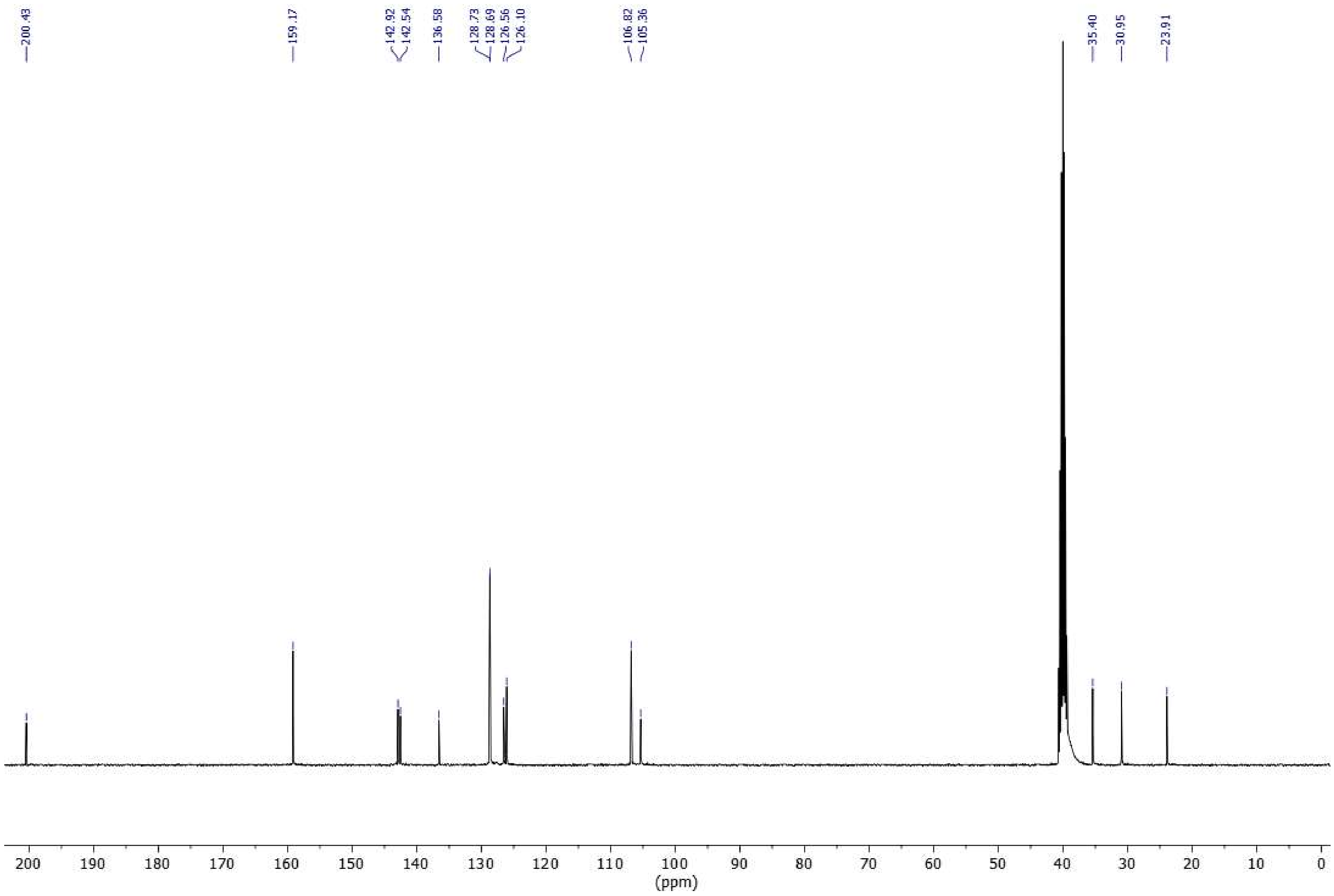
200.43
159.17
142.92
142.54
136.58
128.73
128.69
126.56
126.10
106.82
105.36
35.40
30.95
23.91
200 190 180 170 160 150 140 130 120 110 100 90 80 70 60 50 40 30 20 10 0
(ppm)

HRMS

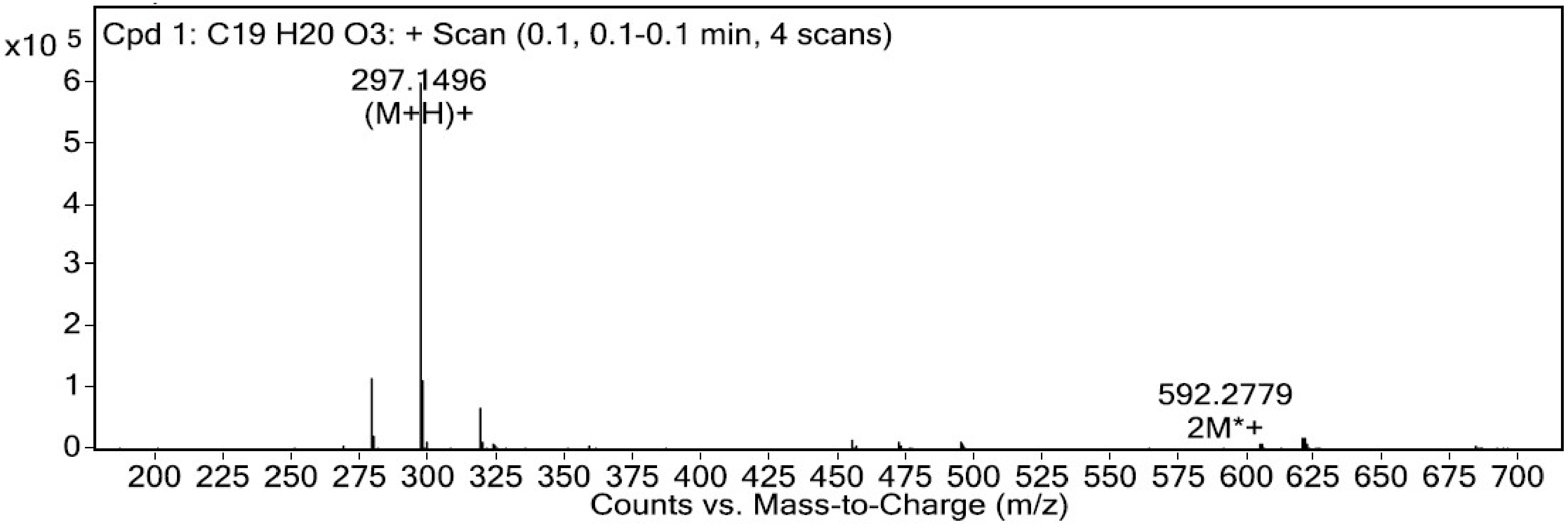

x10 5
Cpd 1: C19 H20 O3: + Scan (0.1, 0.1-0.1 min, 4 scans)
297.1496
(M+H)+
592.2779
2M*+
6
5
4
3
2
1
0
200 225 250 275 300 325 350 375 400 425 450 475 500 525 550 575 600 625 650 675 700
Counts vs. Mass-to-Charge (m/z)

HPLC

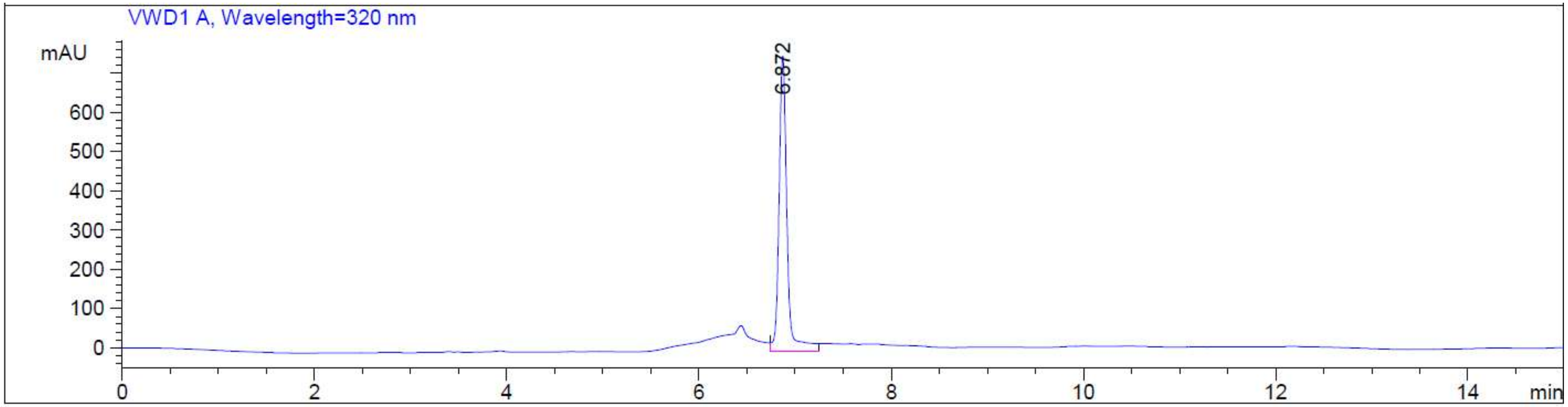

VWD1 A, Wavelength=320 nm
mAU
6.872
600
500
400
300
200
100
0
0 2 4 6 8 10 12 14 min

1H NMR

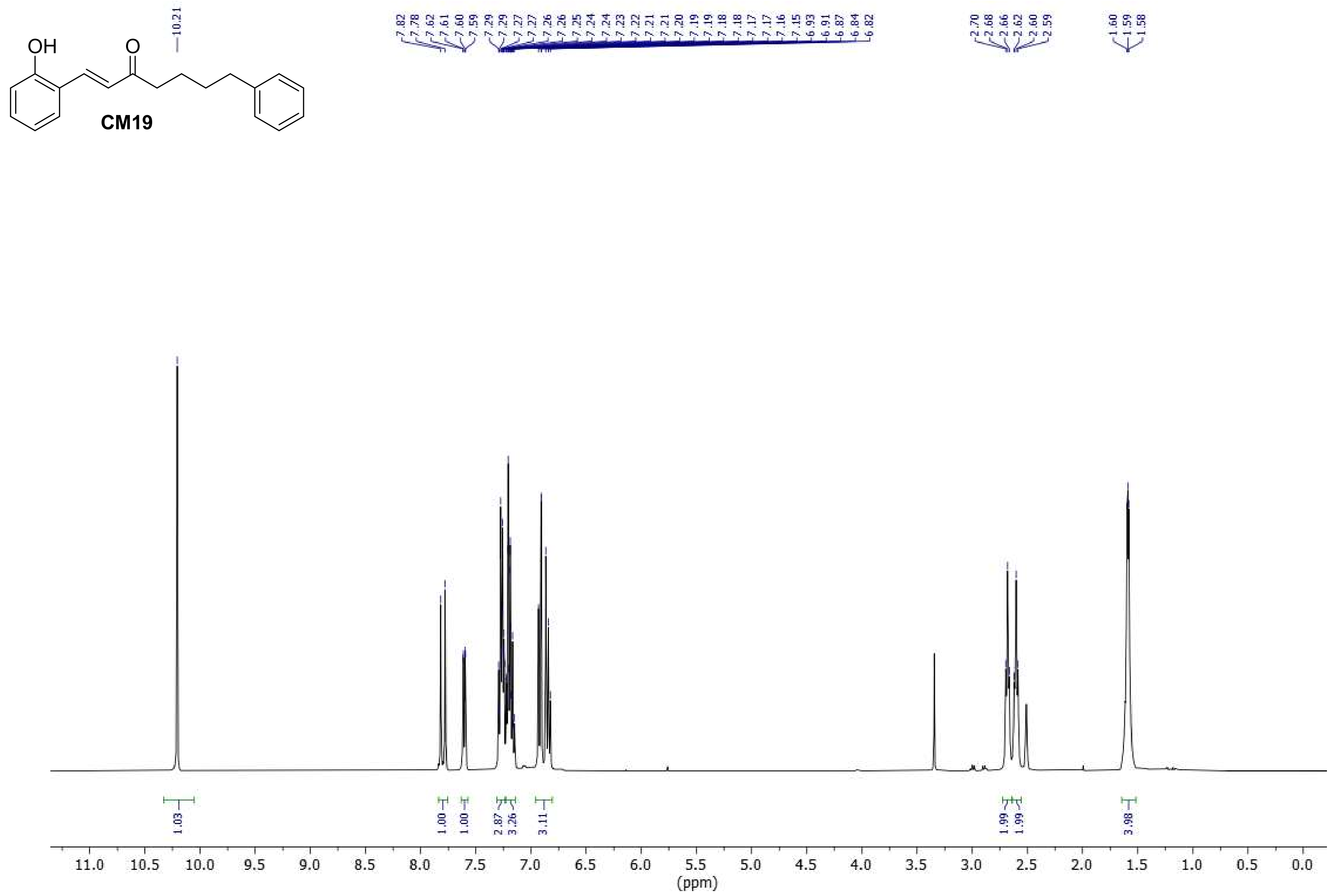

OH
O
CM19
10.21
7.82
7.78
7.62
7.61
7.60
7.59
7.29
7.29
7.27
7.27
7.26
7.26
7.25
7.24
7.24
7.23
7.22
7.21
7.21
7.20
7.19
7.19
7.18
7.18
7.17
7.17
7.16
7.15
6.93
6.91
6.87
6.84
6.82
2.70
2.68
2.66
2.62
2.60
2.59
1.60
1.59
1.58
1.03
1.00
1.00
2.87
3.26
3.11
1.99
1.99
3.98
11.0
10.5
10.0
9.5
9.0
8.5
8.0
7.5
7.0
6.5
6.0
5.5
5.0
4.5
4.0
3.5
3.0
2.5
2.0
1.5
1.0
0.5
0.0
(ppm)

13C NMR

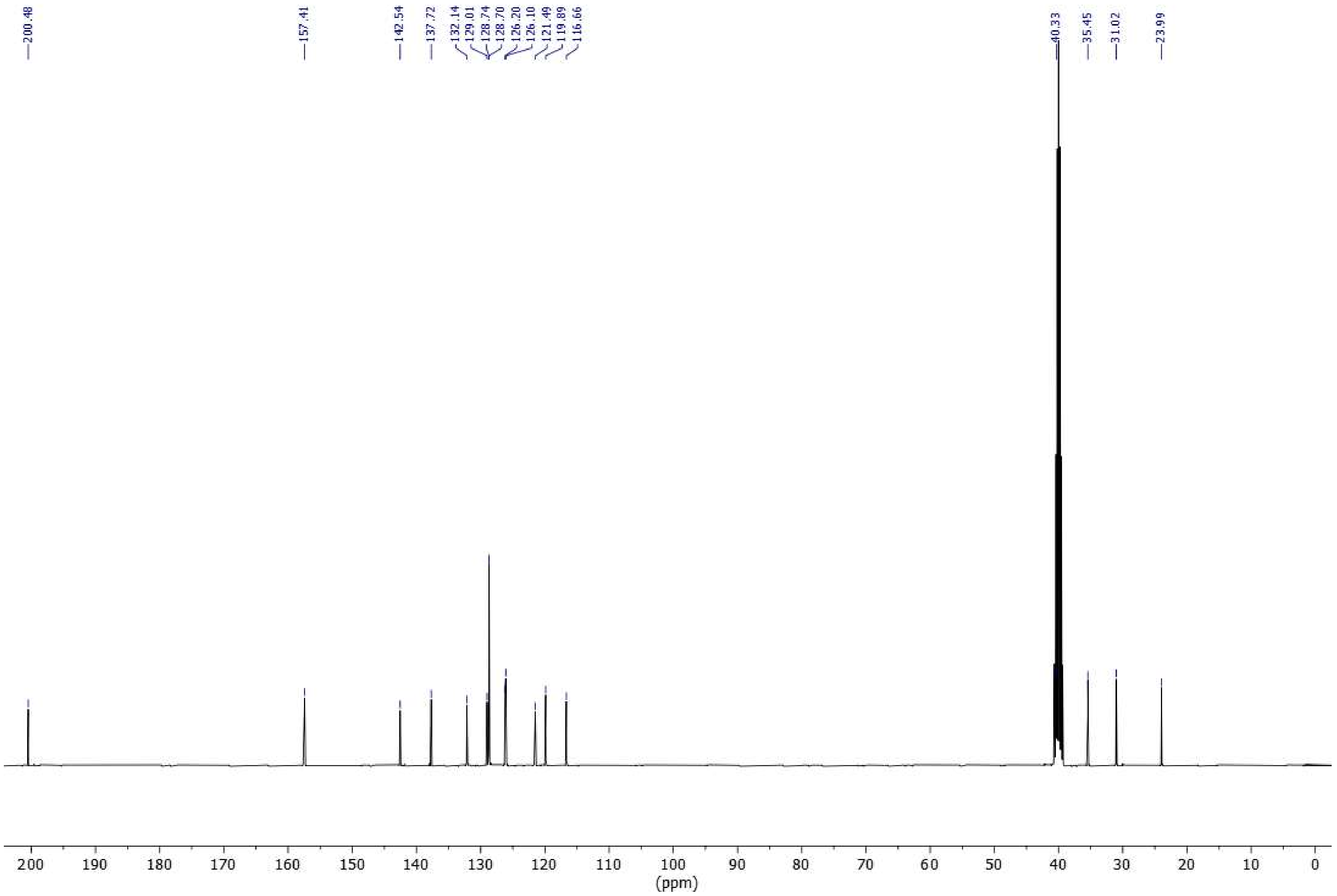
200.48
157.41
142.54
137.72
132.14
129.01
128.74
128.70
126.20
126.10
121.49
119.89
116.66
40.33
35.45
31.02
23.99
200
190
180
170
160
150
140
130
120
110
100
90
80
70
60
50
40
30
20
10
0
(ppm)

HRMS

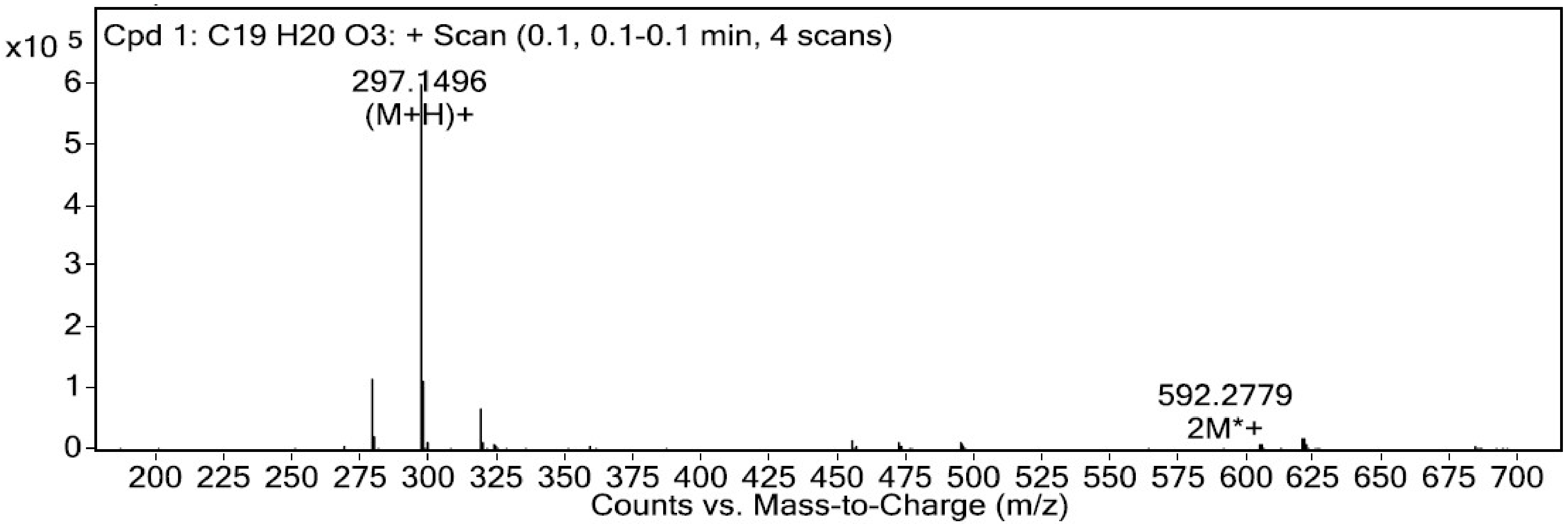

Cpd 1: C19 H20 O3: + Scan (0.1, 0.1-0.1 min, 4 scans)
x10 5
297.1496
(M+H)+
592.2779
2M*+
Counts vs. Mass-to-Charge (m/z)

HPLC

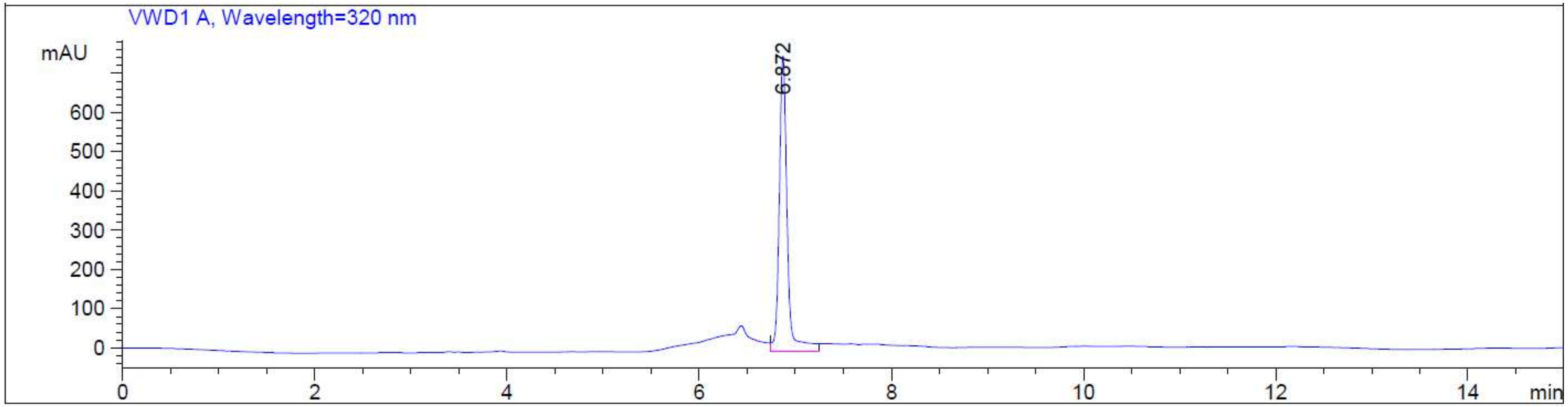

VWD1 A, Wavelength=320 nm
mAU
6.872
min

1H NMR

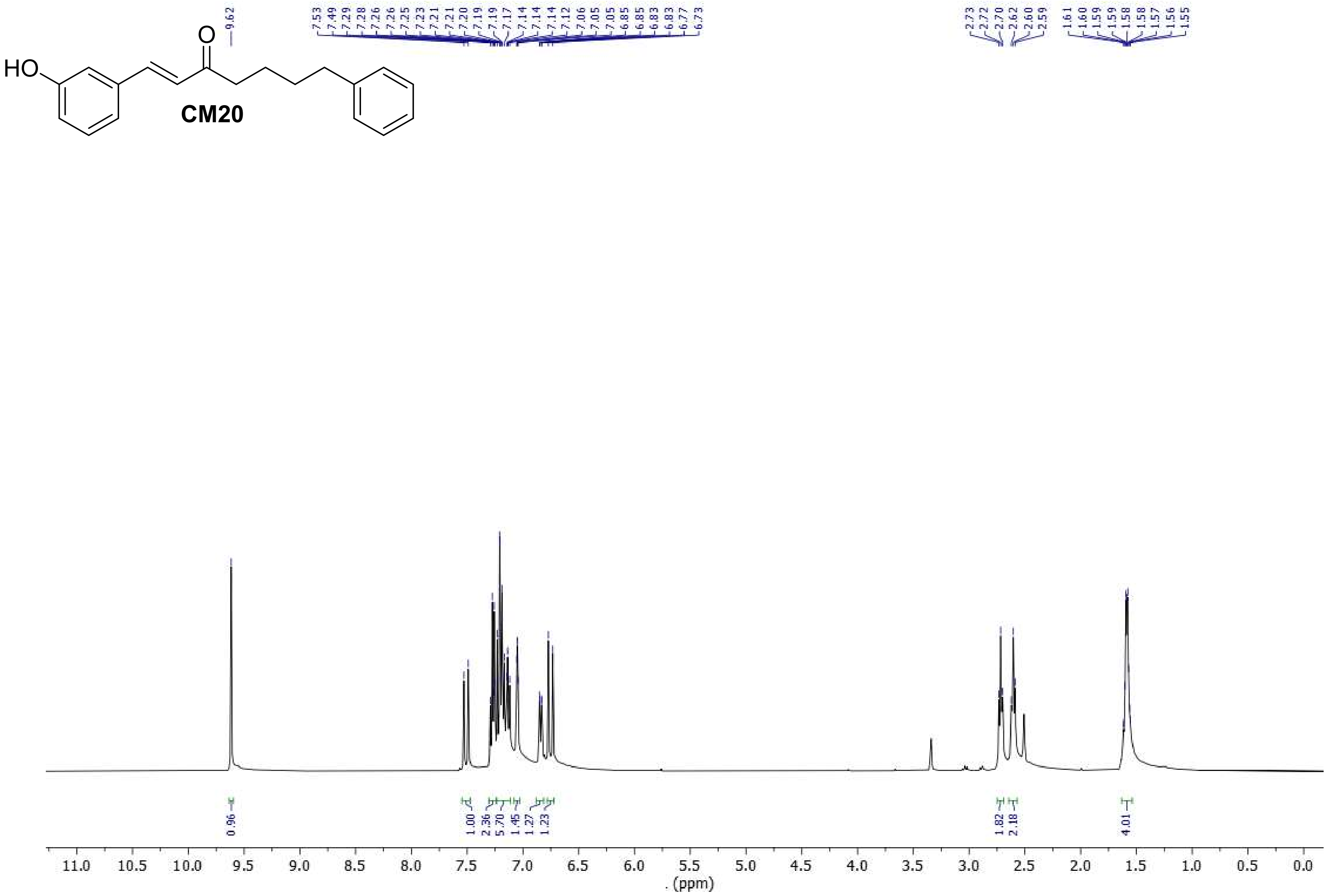
HO
O
CM20
9.62
7.53 7.49 7.29 7.28 7.26 7.26 7.25 7.23 7.21 7.21 7.20 7.19 7.19 7.17 7.14 7.14 7.14 7.12 7.06 7.05 7.05 6.85 6.85 6.83 6.83 6.77 6.73
2.73 2.72 2.70 2.62 2.60 2.59
1.61 1.60 1.59 1.59 1.58 1.58 1.57 1.56 1.55
0.96
1.00 2.36 5.70 1.45 1.27 1.23
1.82 2.18
4.01
11.0 10.5 10.0 9.5 9.0 8.5 8.0 7.5 7.0 6.5 6.0 5.5 5.0 4.5 4.0 3.5 3.0 2.5 2.0 1.5 1.0 0.5 0.0
. (ppm)

13C NMR

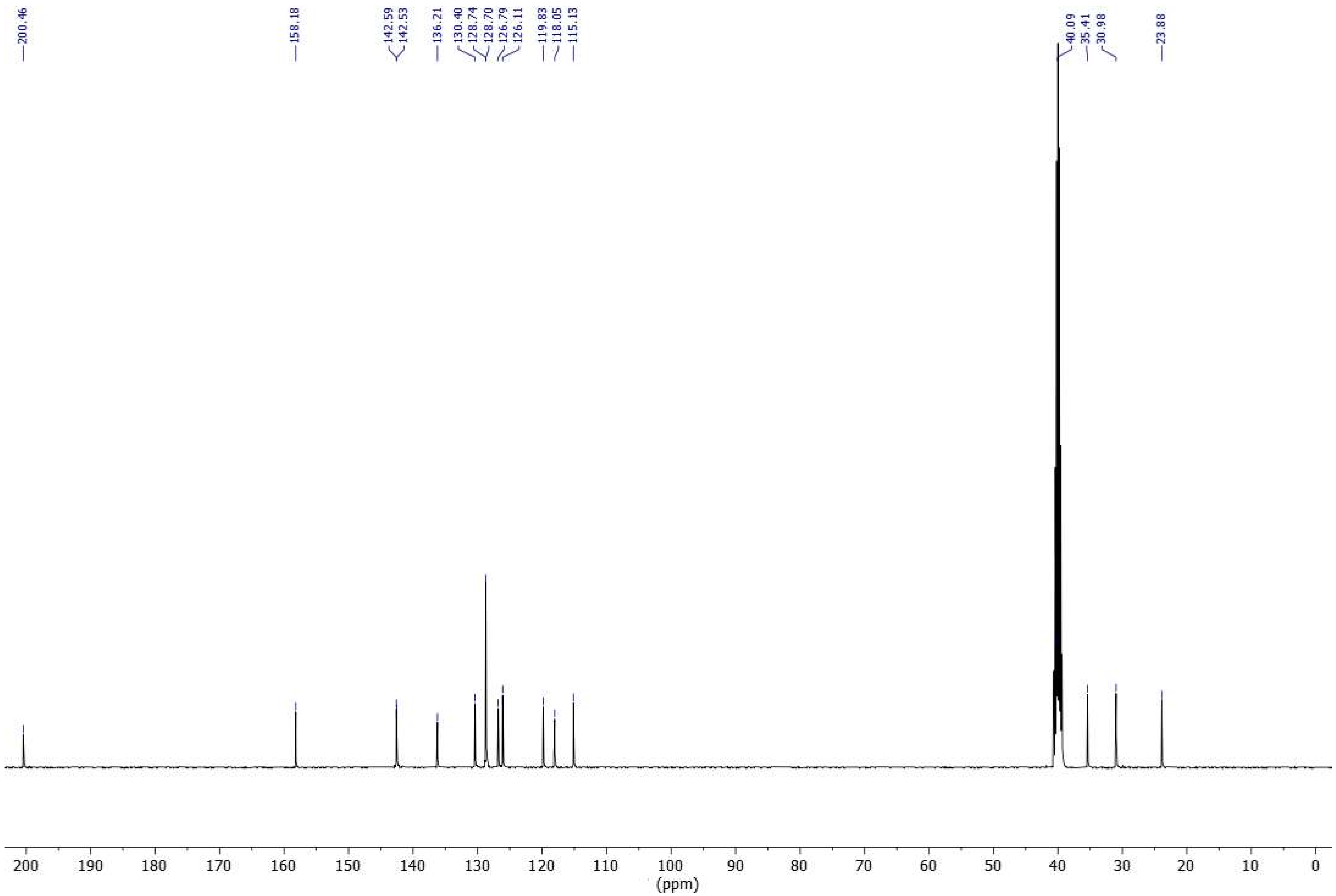
200.46
158.18
142.59
142.53
136.21
130.40
128.74
128.70
126.79
126.11
119.83
118.05
115.13
40.09
35.41
30.98
23.88
200
190
180
170
160
150
140
130
120
110
100
90
80
70
60
50
40
30
20
10
0
(ppm)

HRMS

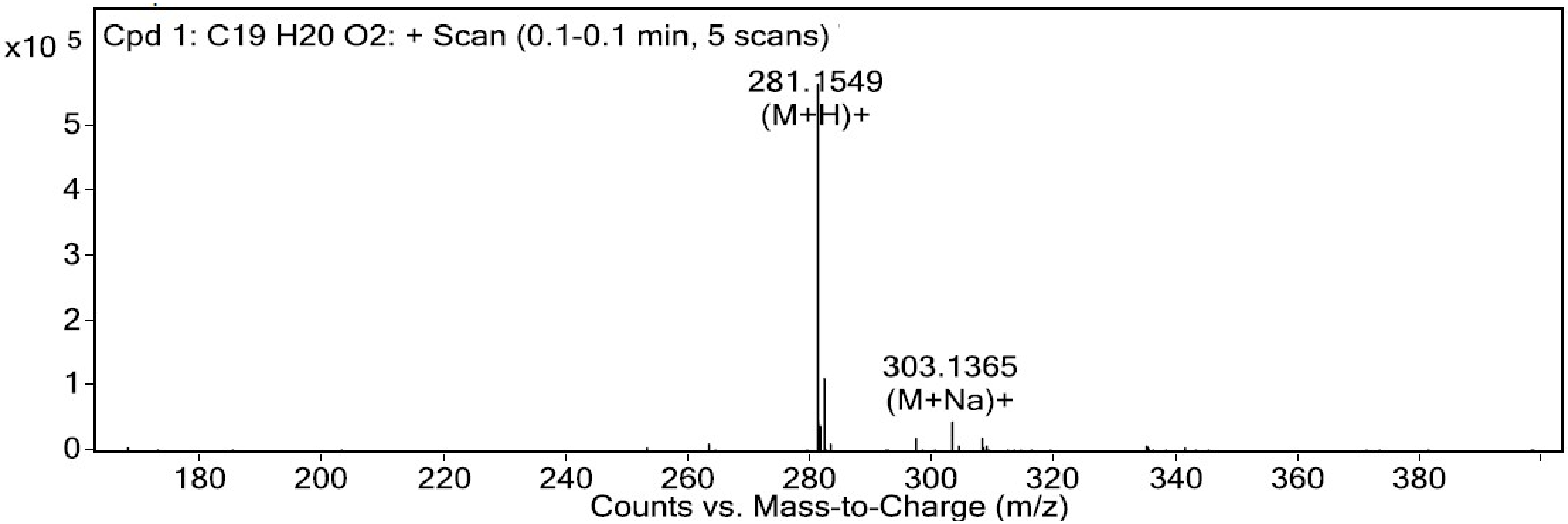
x10 5
Cpd 1: C19 H20 O2: + Scan (0.1-0.1 min, 5 scans)
281.1549
(M+H)+
303.1365
(M+Na)+
0
1
2
3
4
5
180
200
220
240
260
280
300
320
340
360
380
Counts vs. Mass-to-Charge (m/z)

HPLC

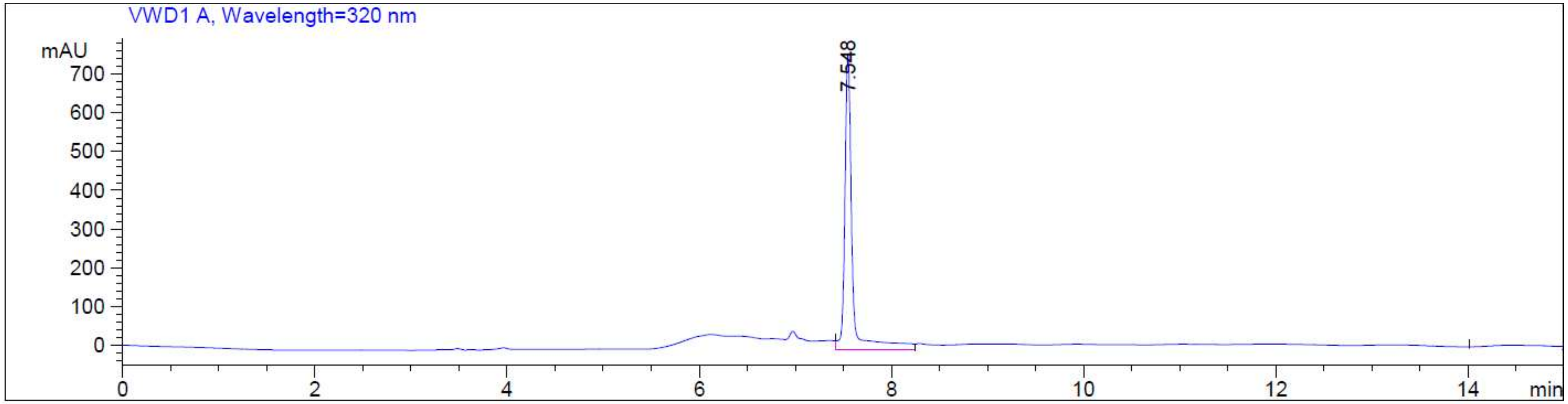
VWD1 A, Wavelength=320 nm
mAU
700
600
500
400
300
200
100
0
7.548
0
2
4
6
8
10
12
14
min

1H

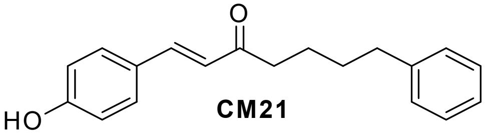
HO
O

CM21

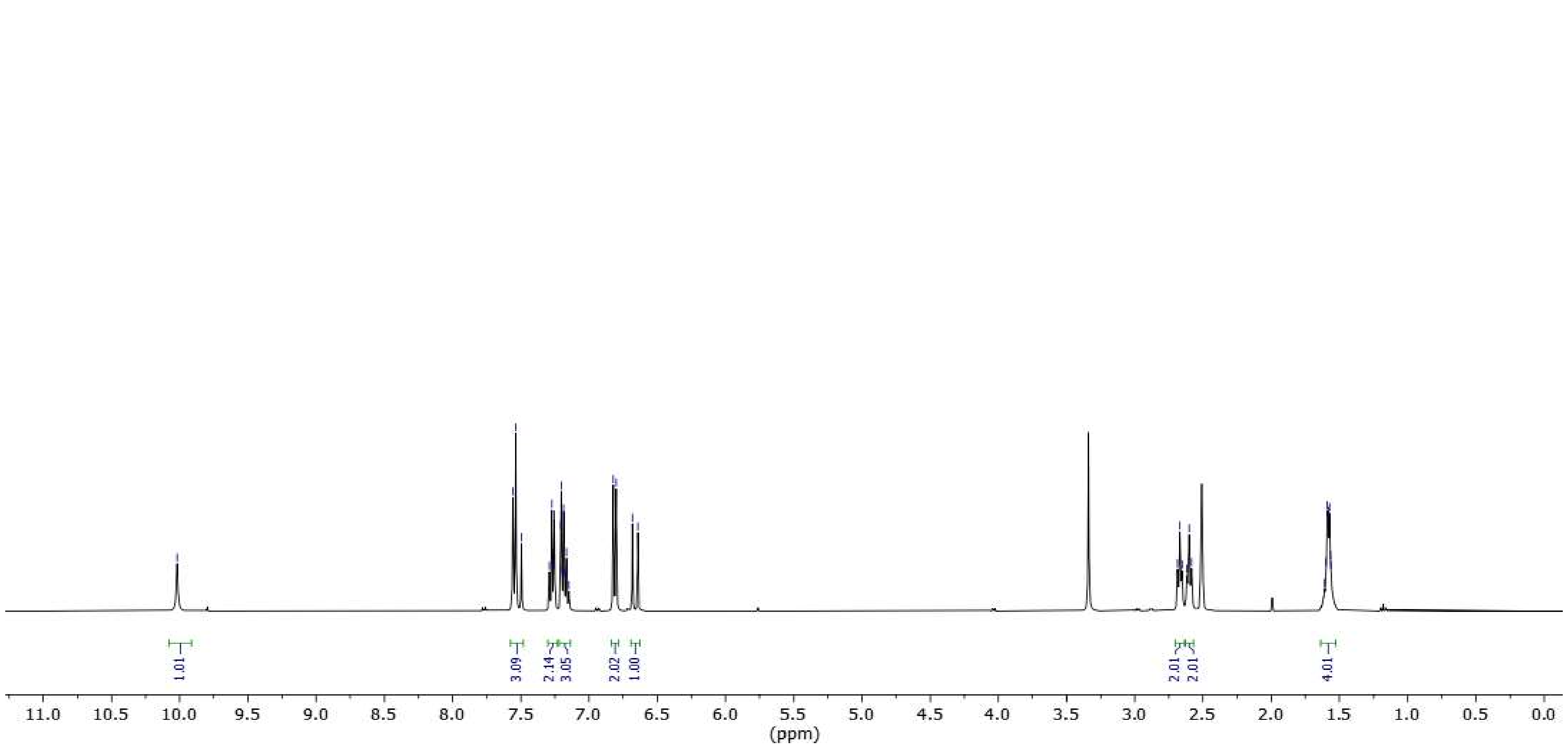
10.02
7.56 7.54 7.50 7.29 7.27 7.26 7.25 7.21 7.20 7.20 7.19 7.18 7.18 7.17 7.17 7.17 7.15 6.82 6.80 6.68 6.64
2.69 2.67 2.65 2.62 2.60 2.58 1.61 1.60 1.59 1.58 1.58 1.57 1.56 1.56
1.01
3.09 2.14 3.05 2.02 1.00
2.01 2.01
4.01
11.0 10.5 10.0 9.5 9.0 8.5 8.0 7.5 7.0 6.5 6.0 5.5 5.0 4.5 4.0 3.5 3.0 2.5 2.0 1.5 1.0 0.5 0.0
(ppm)

HRMS

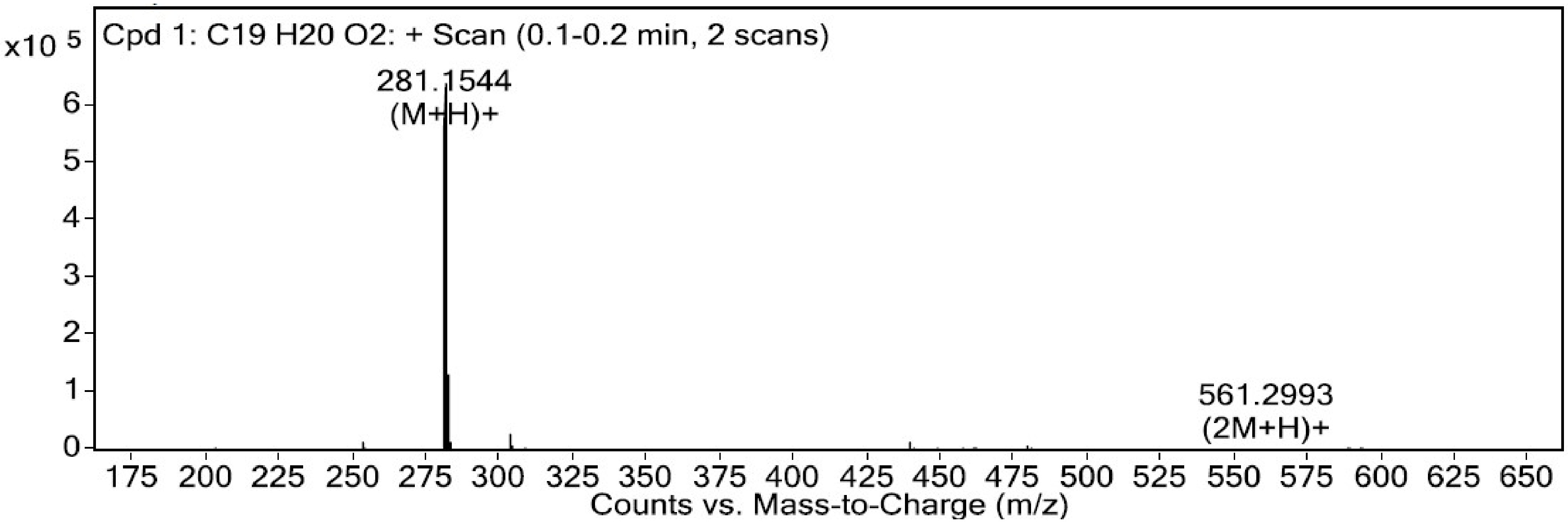

x10 5
Cpd 1: C19 H20 O2: + Scan (0.1-0.2 min, 2 scans)
281.1544
(M+H)+
561.2993
(2M+H)+
0
1
2
3
4
5
6
175 200 225 250 275 300 325 350 375 400 425 450 475 500 525 550 575 600 625 650
Counts vs. Mass-to-Charge (m/z)

HPLC

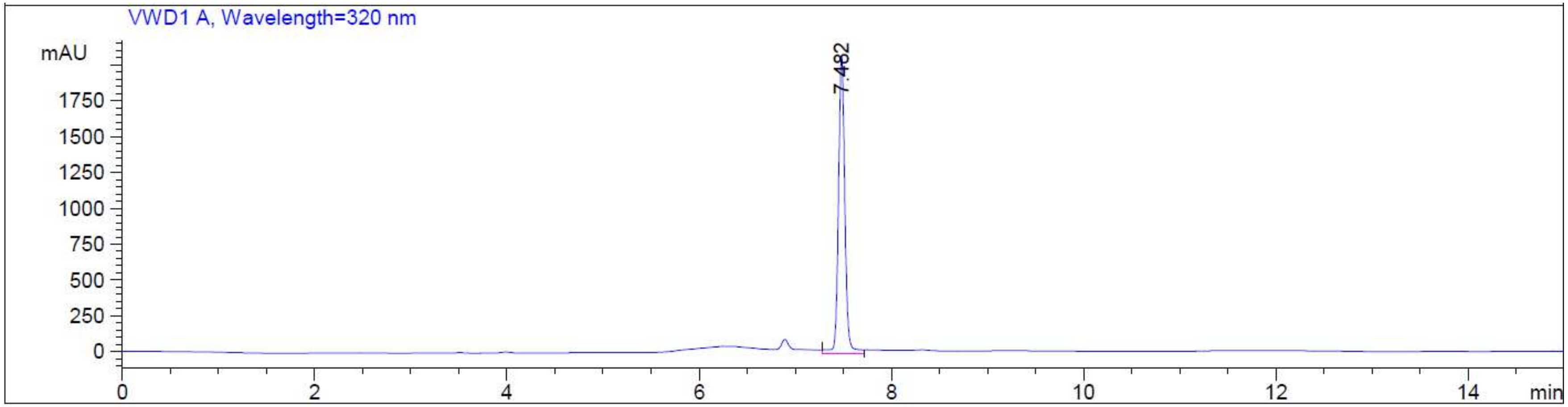

VWD1 A, Wavelength=320 nm
mAU
1750
1500
1250
1000
750
500
250
0
7.482
0
2
4
6
8
10
12
14
min

1H NMR

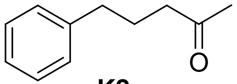

K2

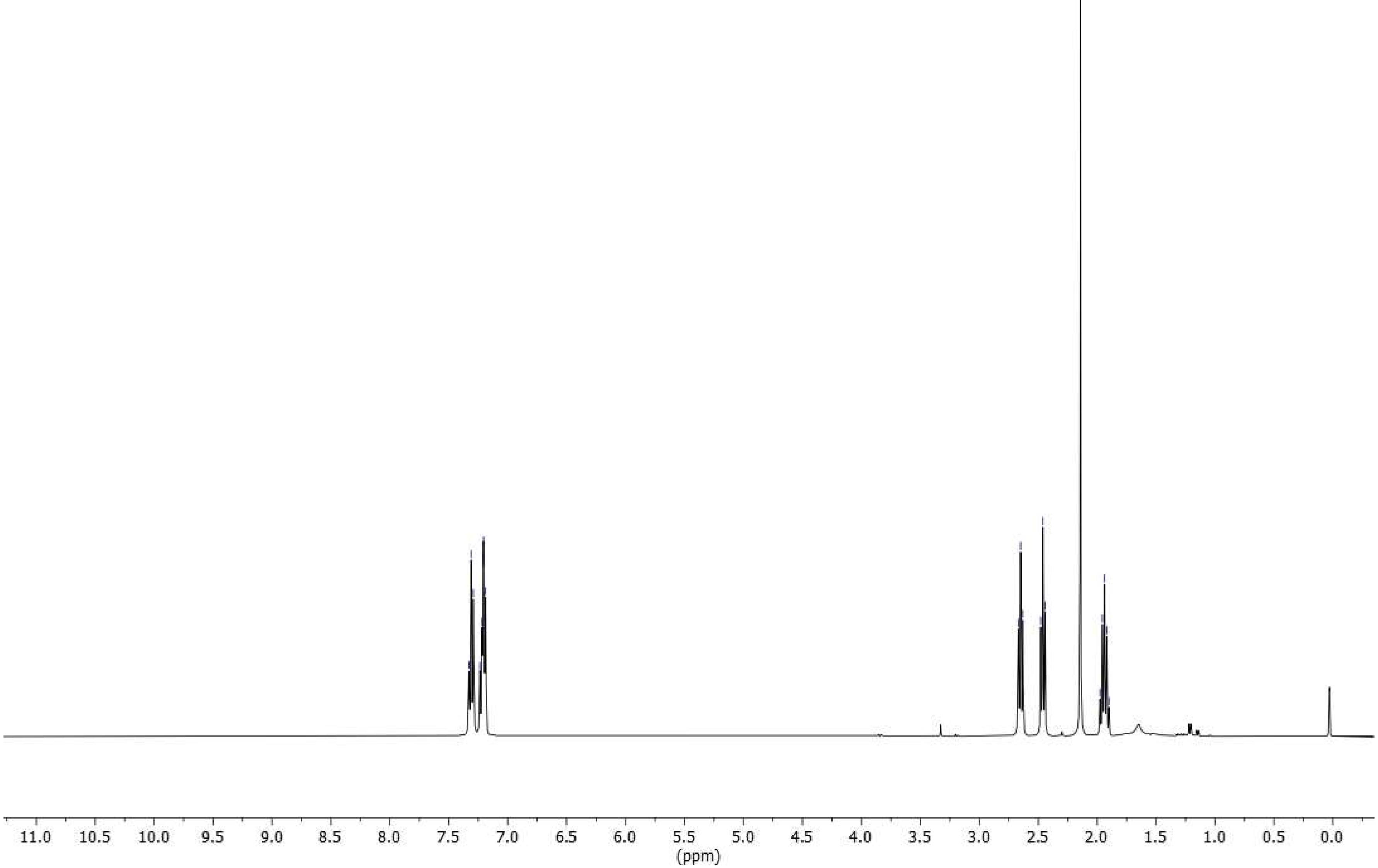
7.33
7.31
7.29
7.24
7.22
7.21
7.20
7.19
2.67
2.65
2.63
2.48
2.46
2.44
2.14
1.97
1.96
1.94
1.92
1.90
11.0 10.5 10.0 9.5 9.0 8.5 8.0 7.5 7.0 6.5 6.0 5.5 5.0 4.5 4.0 3.5 3.0 2.5 2.0 1.5 1.0 0.5 0.0
(ppm)

13C NMR

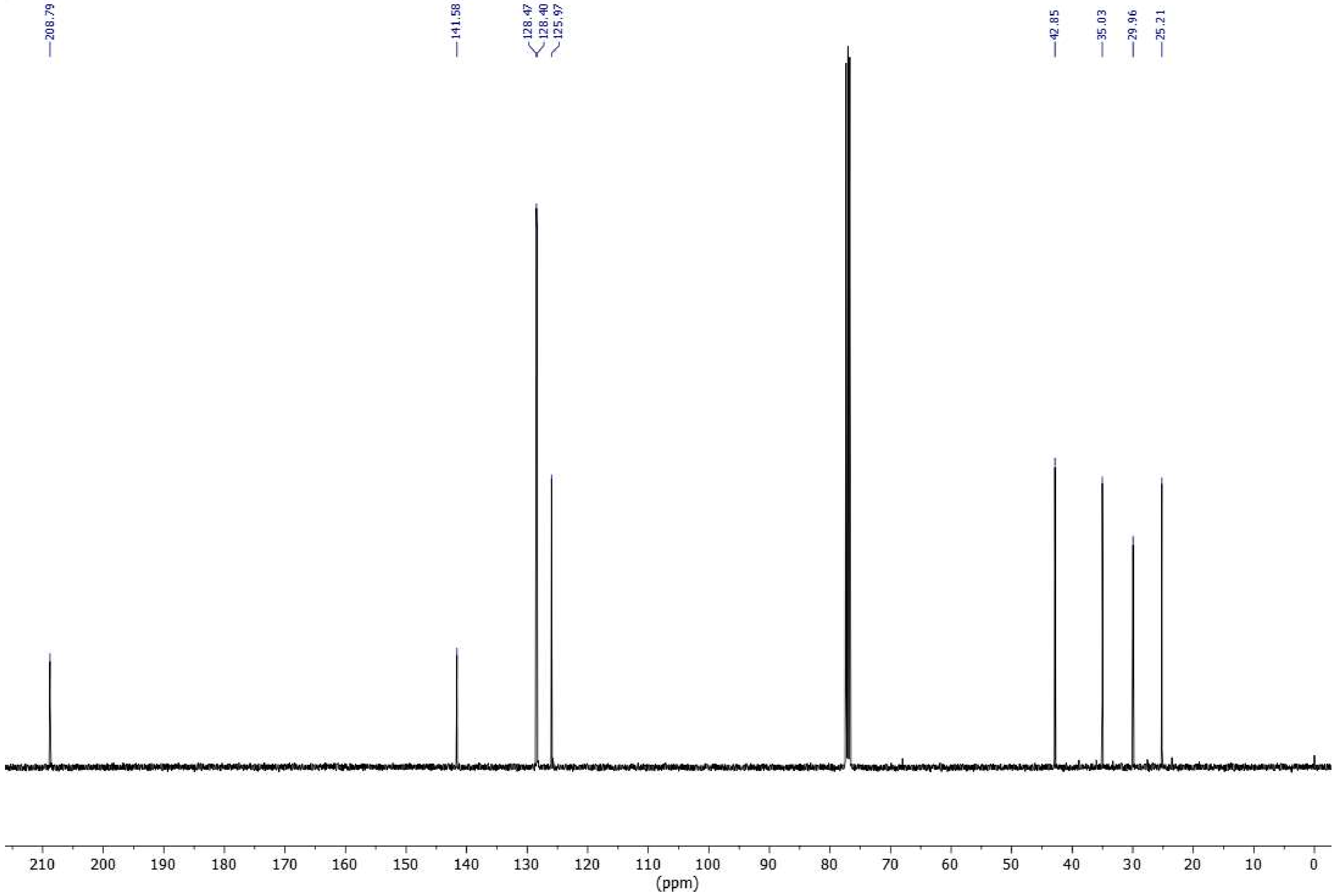

208.79
141.58
128.47
128.40
125.97
42.85
35.03
29.96
25.21
210
200
190
180
170
160
150
140
130
120
110
100
90
80
70
60
50
40
30
20
10
0
(ppm)

1H NMR

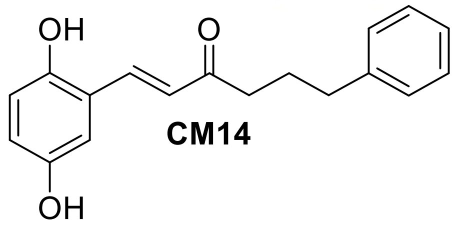

CM14

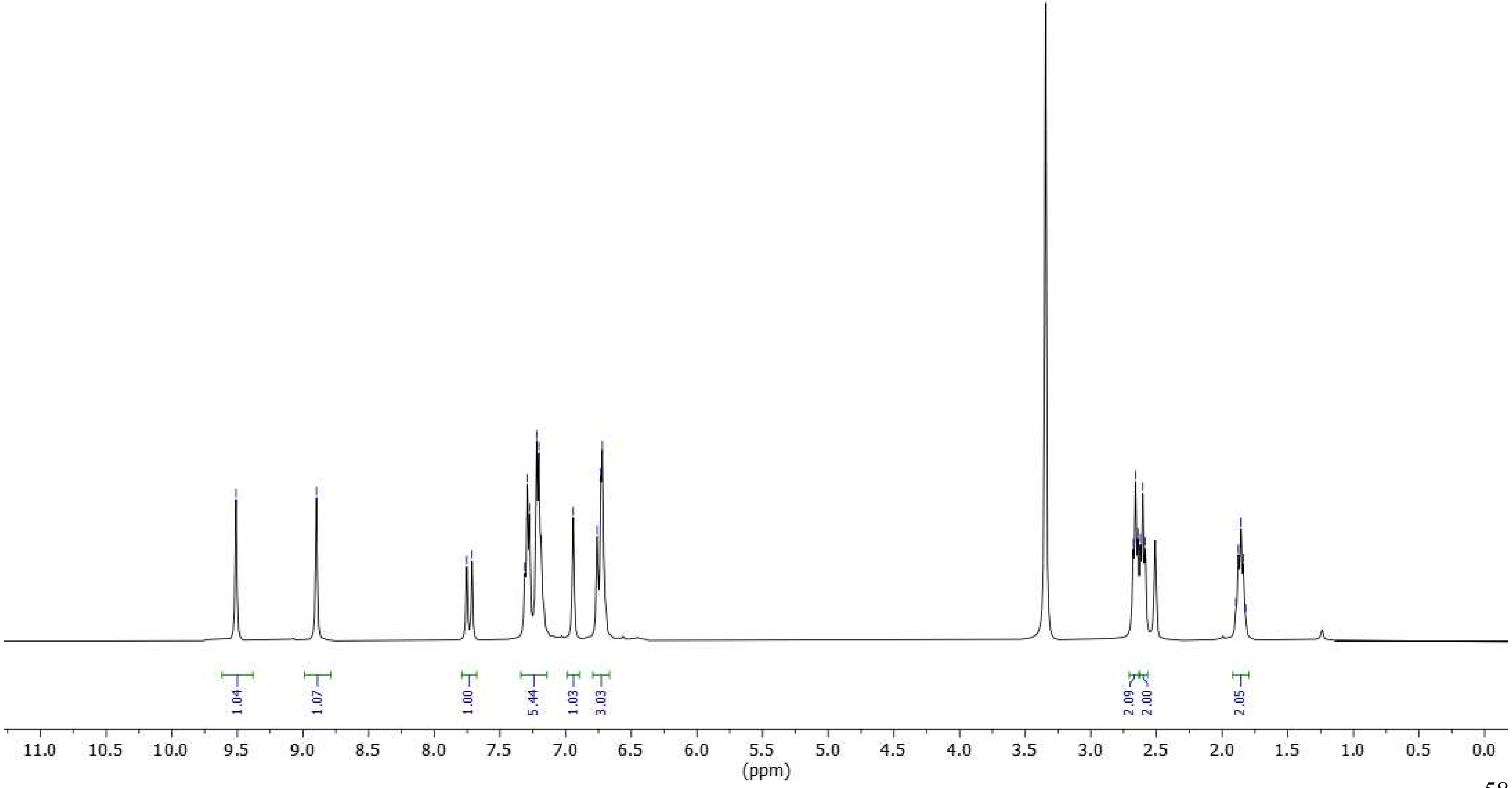
9.51
8.90
7.75
7.71
7.31
7.29
7.27
7.22
7.20
7.19
6.94
6.76
6.73
6.72
2.68
2.66
2.64
2.62
2.60
2.59
1.90
1.88
1.86
1.84
1.82
1.04
1.07
1.00
5.44
1.03
3.03
2.09
2.00
2.05
11.0
10.5
10.0
9.5
9.0
8.5
8.0
7.5
7.0
6.5
6.0
5.5
5.0
4.5
4.0
3.5
3.0
2.5
2.0
1.5
1.0
0.5
0.0
(ppm)

13C NMR

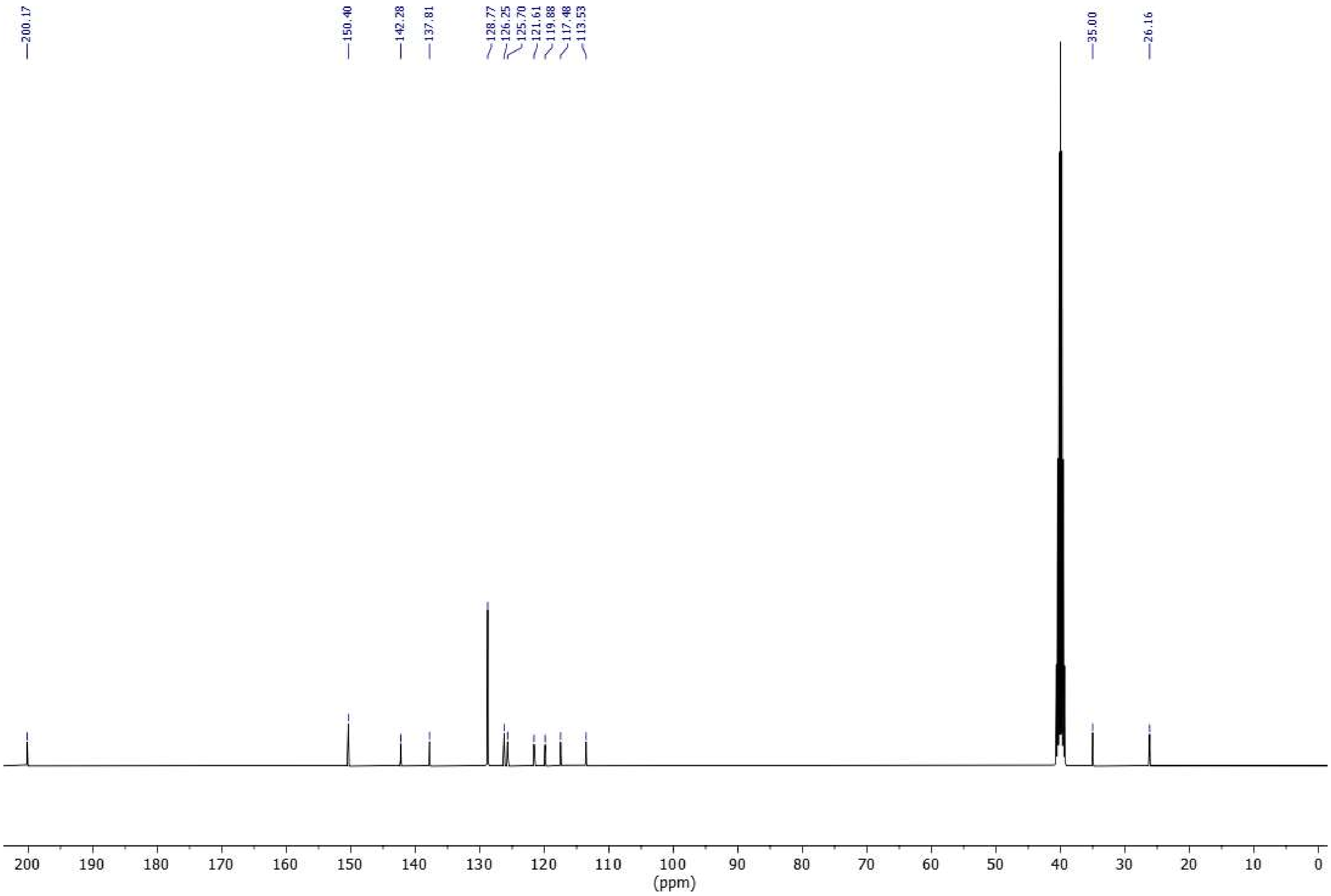
200.17
150.40
142.28
137.81
128.77
126.25
125.70
121.61
119.88
117.48
113.53
35.00
26.16
200 190 180 170 160 150 140 130 120 110 100 90 80 70 60 50 40 30 20 10 0
(ppm)

HRMS

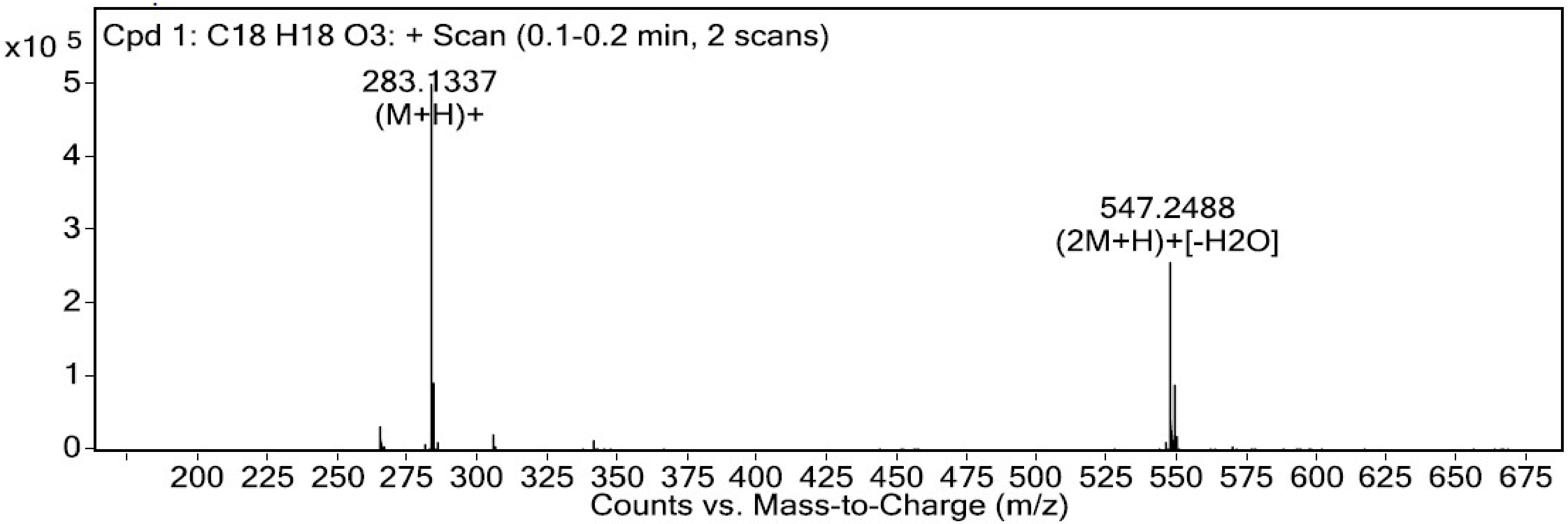

x10 5
Cpd 1: C18 H18 O3: + Scan (0.1-0.2 min, 2 scans)
283.1337
(M+H)+
547.2488
(2M+H)+[-H2O]
5
4
3
2
1
0
200 225 250 275 300 325 350 375 400 425 450 475 500 525 550 575 600 625 650 675
Counts vs. Mass-to-Charge (m/z)

HPLC

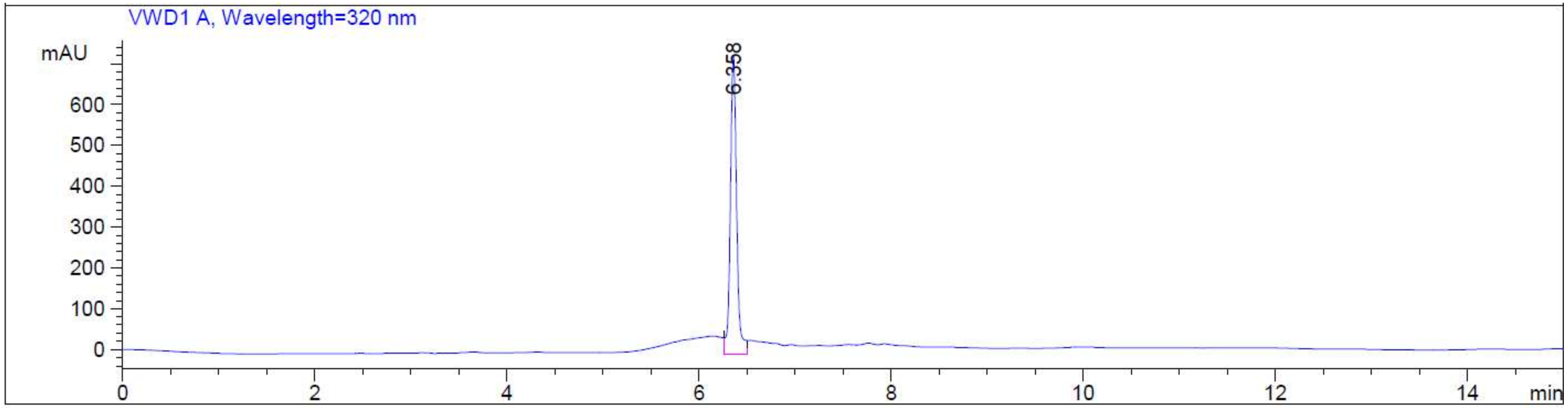

VWD1 A, Wavelength=320 nm
mAU
600
500
400
300
200
100
0
6.358
0
2
4
6
8
10
12
14
min

1H NMR

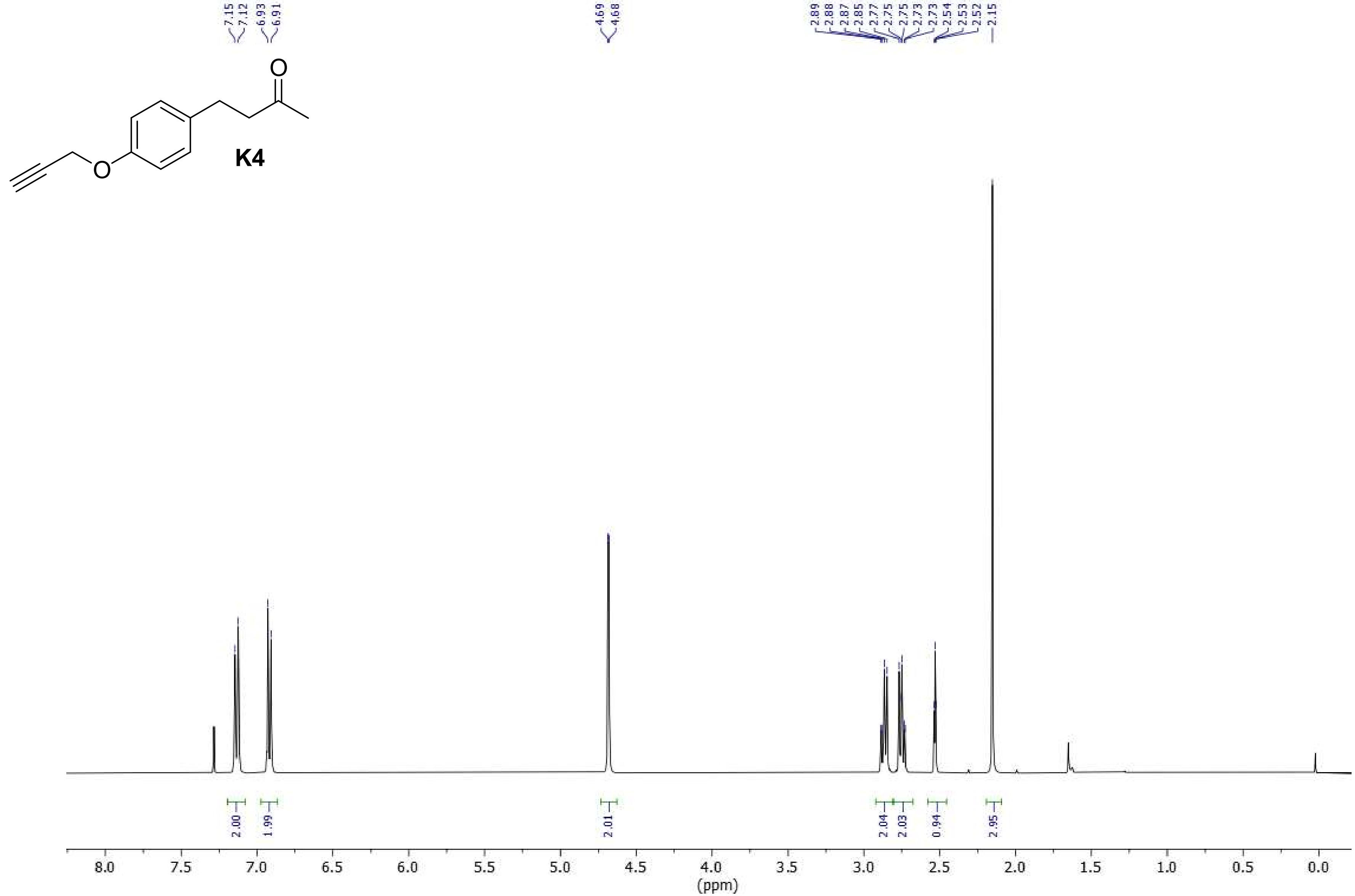
7.15
7.12
6.93
6.91
4.69
4.68
2.89
2.88
2.87
2.85
2.77
2.75
2.75
2.73
2.73
2.54
2.53
2.52
2.15
O
O
K4
2.00
1.99
2.01
2.04
2.03
0.94
2.95
8.0
7.5
7.0
6.5
6.0
5.5
5.0
4.5
4.0
3.5
3.0
2.5
2.0
1.5
1.0
0.5
0.0
(ppm)

13C NMR

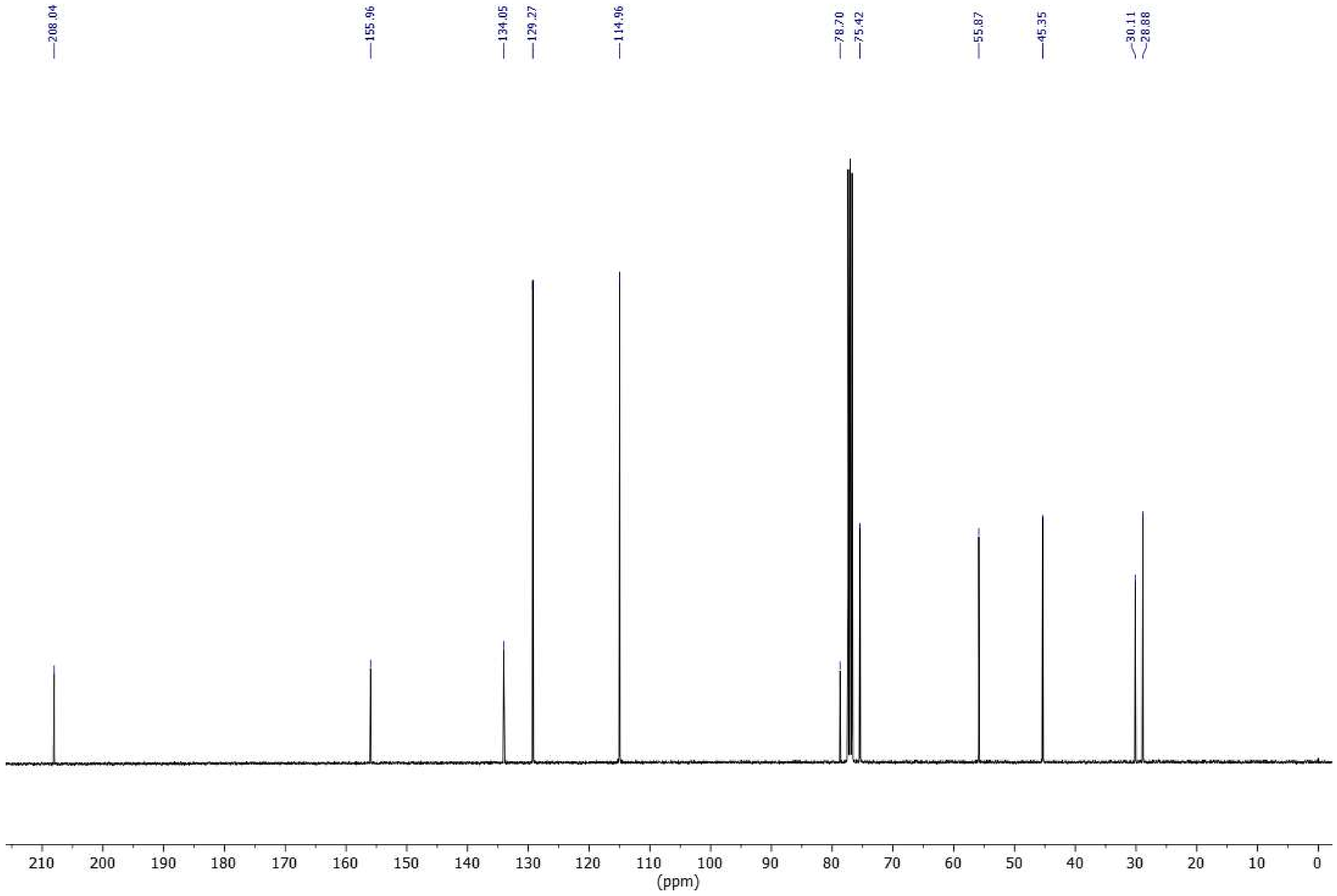

208.04
155.96
134.05
129.27
114.96
78.70
75.42
55.87
45.35
30.11
28.88
210 200 190 180 170 160 150 140 130 120 110 100 90 80 70 60 50 40 30 20 10 0
(ppm)

HRMS

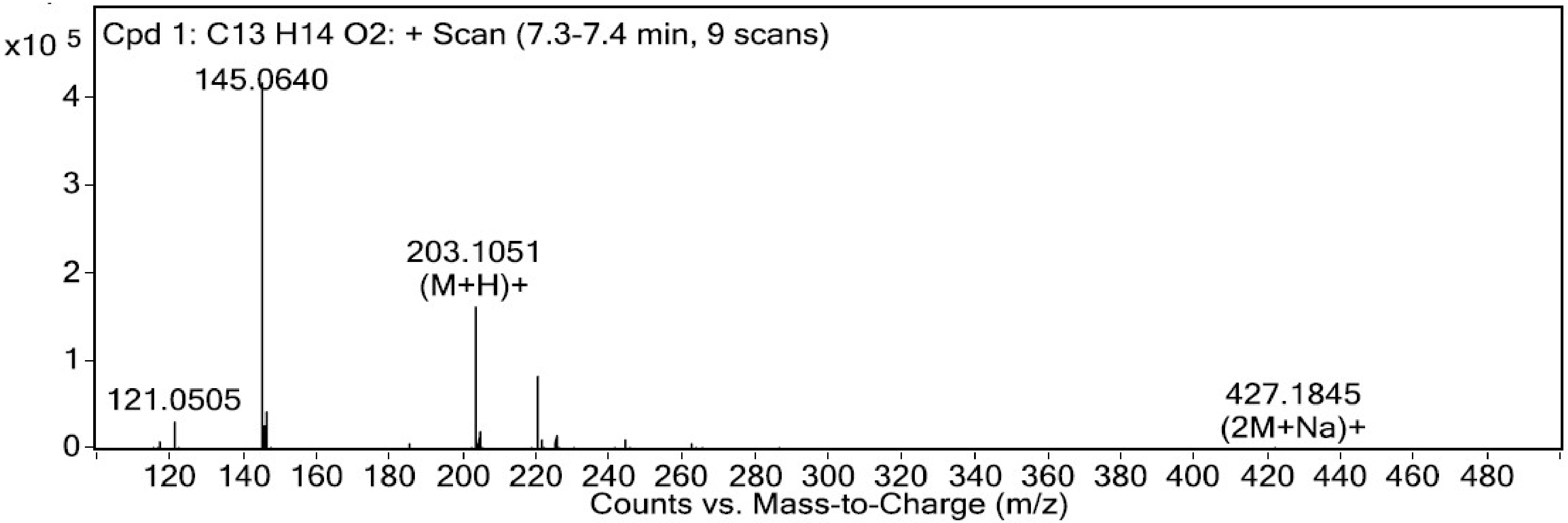

x10 5
Cpd 1: C13 H14 O2: + Scan (7.3-7.4 min, 9 scans)
145.0640
203.1051
(M+H)+
121.0505
427.1845
(2M+Na)+
0
1
2
3
4
120 140 160 180 200 220 240 260 280 300 320 340 360 380 400 420 440 460 480
Counts vs. Mass-to-Charge (m/z)

1H NMR

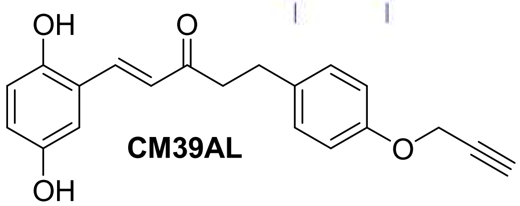

CM39AL

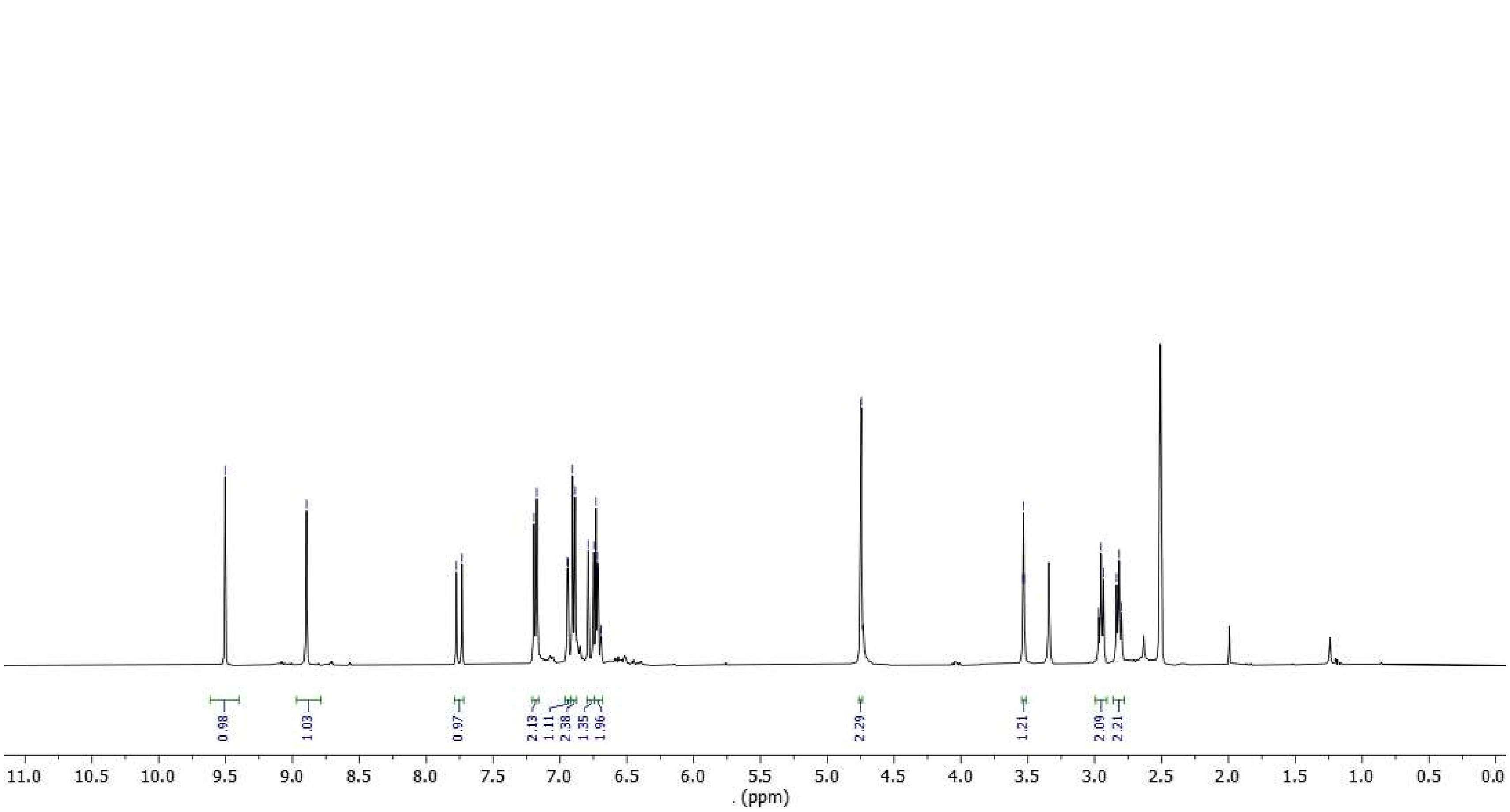

13C NMR

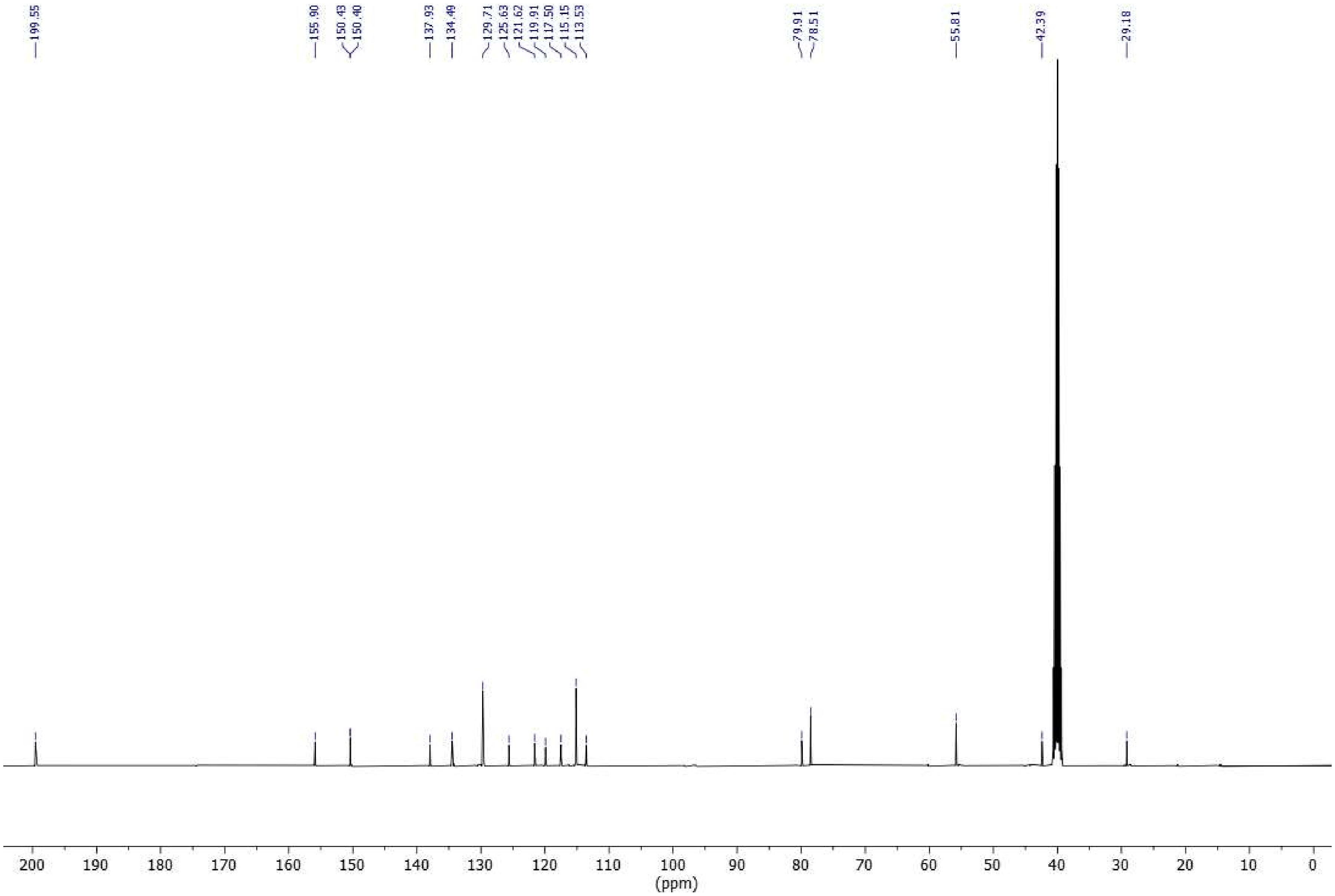

199.55
155.90
150.43
150.40
137.93
134.49
129.71
125.63
121.62
119.91
117.50
115.15
113.53
79.91
78.51
55.81
42.39
29.18
200
190
180
170
160
150
140
130
120
110
100
90
80
70
60
50
40
30
20
10
0
(ppm)

HRMS

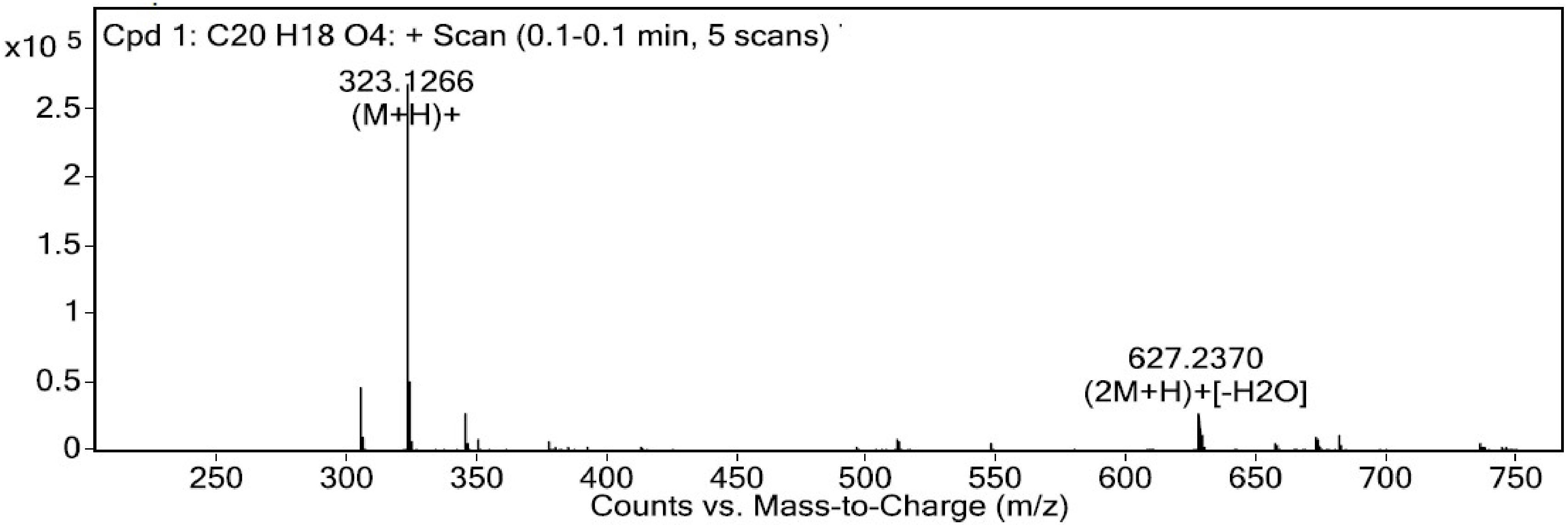

x10 5
Cpd 1: C20 H18 O4: + Scan (0.1-0.1 min, 5 scans)
323.1266
(M+H)+
627.2370
(2M+H)+[-H2O]
2.5
2
1.5
1
0.5
0
250
300
350
400
450
500
550
600
650
700
750
Counts vs. Mass-to-Charge (m/z)

HPLC

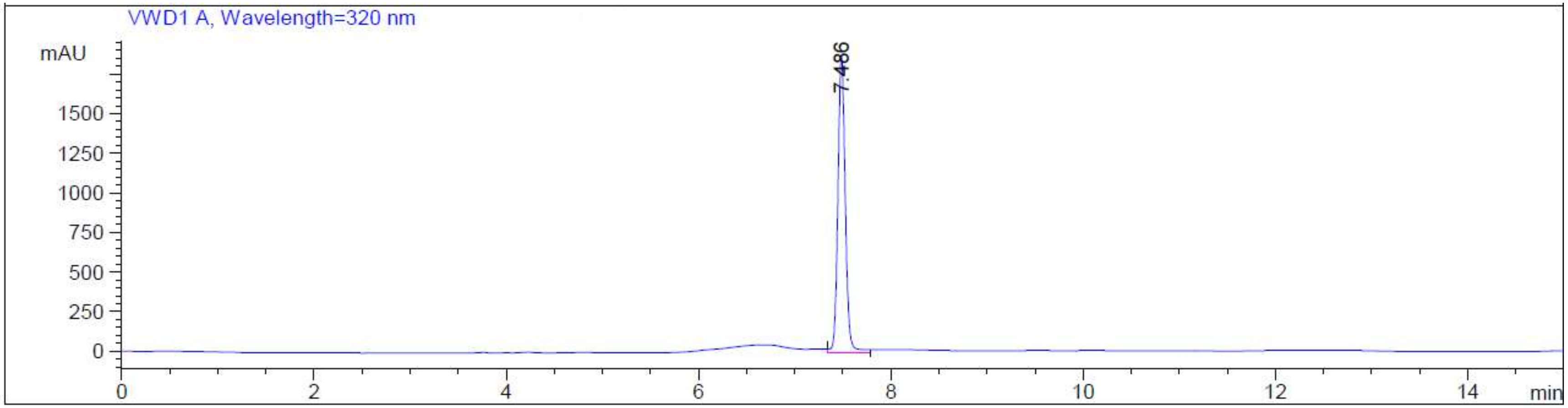

VWD1 A, Wavelength=320 nm
mAU
1500
1250
1000
750
500
250
0
7.486
0
2
4
6
8
10
12
14
min

References

[1] Demichev V, Messner CB, Vernardis SI, Lilley KS, Ralser M. DIA-NN: neural networks and interference correction enable deep proteome coverage in high throughput. Nat Methods 2020;17:41–4. https://doi.org/10.1038/S41592-019-0638-X.

[2] Lindahl E, Hess B, van der Spoel D. GROMACS 3.0: A package for molecular simulation and trajectory analysis. J Mol Model 2001;7:306–17. https://doi.org/10.1007/S008940100045.

[3] Lindahl E, Hess B, van der Spoel D. GROMACS 3.0: A package for molecular simulation and trajectory analysis. J Mol Model 2001;7:306–17. https://doi.org/10.1007/S008940100045.

[4] Huang J, Mackerell AD. CHARMM36 all-atom additive protein force field: Validation based on comparison to NMR data. J Comput Chem 2013;34:2135–45. https://doi.org/10.1002/jcc.23354.

[5] Jorgensen WL, Chandrasekhar J, Madura JD, Impey RW, Klein ML. Comparison of simple potential functions for simulating liquid water. J Chem Phys 1983;79:926–35. https://doi.org/10.1063/1.445869.

[6] Wieczorek M, Urnavicius L, Ti SC, Molloy KR, Chait BT, Kapoor TM. Asymmetric Molecular Architecture of the Human γ-Tubulin Ring Complex. Cell 2020;180:165-175.e16. https://doi.org/10.1016/J.CELL.2019.12.007.

[7] Liu Y, Yang X, Gan J, Chen S, Xiao ZX, Cao Y. CB-Dock2: improved protein–ligand blind docking by integrating cavity detection, docking and homologous template fitting. Nucleic Acids Res 2022;50:W159–64. https://doi.org/10.1093/NAR/GKAC394.

[8] Yang X, Liu Y, Gan J, Xiao ZX, Cao Y. FitDock: protein–ligand docking by template fitting. Brief Bioinform 2022;23:1–11. https://doi.org/10.1093/BIB/BBAC087.

[9] Selka A, Doiron JA, Lyons P, Dastous S, Chiasson A, Cormier M, et al. Discovery of a novel 2,5-dihydroxycinnamic acid-based 5-lipoxygenase inhibitor that induces apoptosis and may impair autophagic flux in RCC4 renal cancer cells. Eur J Med Chem 2019;179:347–57. https://doi.org/10.1016/J.EJMECH.2019.06.060.

[10] Murugesan A, Lassalle-Claux G, Hogan L, Vaillancourt E, Selka A, Luiker K, et al. Antimyeloma Potential of Caffeic Acid Phenethyl Ester and Its Analogues through Sp1 Mediated Downregulation of IKZF1-IRF4-MYC Axis. J Nat Prod 2020;83:3526–35. https://doi.org/10.1021/ACS.JNATPROD.0C00350.

[11] Sanderson JT, Clabault H, Patton C, Lassalle-Claux G, Jean-François J, Paré F, et al. Antiproliferative, antiandrogenic and cytotoxic effects of novel caffeic acid derivatives in LNCaP human androgen-dependent prostate cancer cells 2013. https://doi.org/10.1016/j.bmc.2013.08.057.

[12] Touaibia M, Hébert MJG, Levesque NA, Doiron JA, Doucet MS, Jean-François J, et al. Sinapic acid phenethyl ester as a potent selective 5-lipoxygenase inhibitor: Synthesis and structure–activity relationship. Chem Biol Drug Des 2018;92:1876–87. https://doi.org/10.1111/cbdd.13360.

[13] Bian L, Cao S, Cheng L, Nakazaki A, Nishikawa T, Qi J. Semi-synthesis and Structure–Activity Relationship of Neuritogenic Oleanene Derivatives. ChemMedChem 2018;13:1972–7. https://doi.org/10.1002/cmdc.201800352.

[14] Ferreira IM, Meira EB, Rosset IG, Porto ALM. Chemoselective biohydrogenation of α,β- and α,β,γ,δ-unsaturated ketones by the marine-derived fungus Penicillium citrinum CBMAI 1186 in a biphasic system. J Mol Catal B Enzym 2015;115:59–65. https://doi.org/10.1016/j.molcatb.2015.01.017.

[15] Touaibia M, Selka A, Levesque NA, St-Onge PA. Green hydrogenation: Solvent-free hydrogenation of pinenes for an undergraduate organic chemistry laboratory. J Chem Educ 2020;97:2296–301. https://doi.org/10.1021/ACS.JCHEMED.9B01026/SUPPL_FILE/ED9B01026_SI_001.PDF.
[16] Hattori K, Sajiki H, Hirota K. Chemoselective control of hydrogenation among aromatic carbonyl and benzyl alcohol derivatives using Pd/C(en) catalyst. Tetrahedron 2001;57:4817–24. https://doi.org/https://doi.org/10.1016/S0040-4020(01)00421-5.
[17] Verdoes M, Florea BI, Hillaert U, Willems LI, Van Der Linden WA, Sae-Heng M, et al. Azido-BODIPY Acid Reveals Quantitative Staudinger-Bertozzi Ligation in Two-Step Activity-Based Proteasome Profiling n.d. https://doi.org/10.1002/cbic.200800231.
