# Supplementary figures and images for: Anti-Cancer Potential of a new Derivative of Caffeic Acid Phenethyl Ester targeting the Centrosome
Source: Redox Biol. 2025 Mar 5;81:103582. doi: 10.1016/j.redox.2025.103582 (PMC11951030; doi:10.1016/j.redox.2025.103582)

Supplementary Figure 1

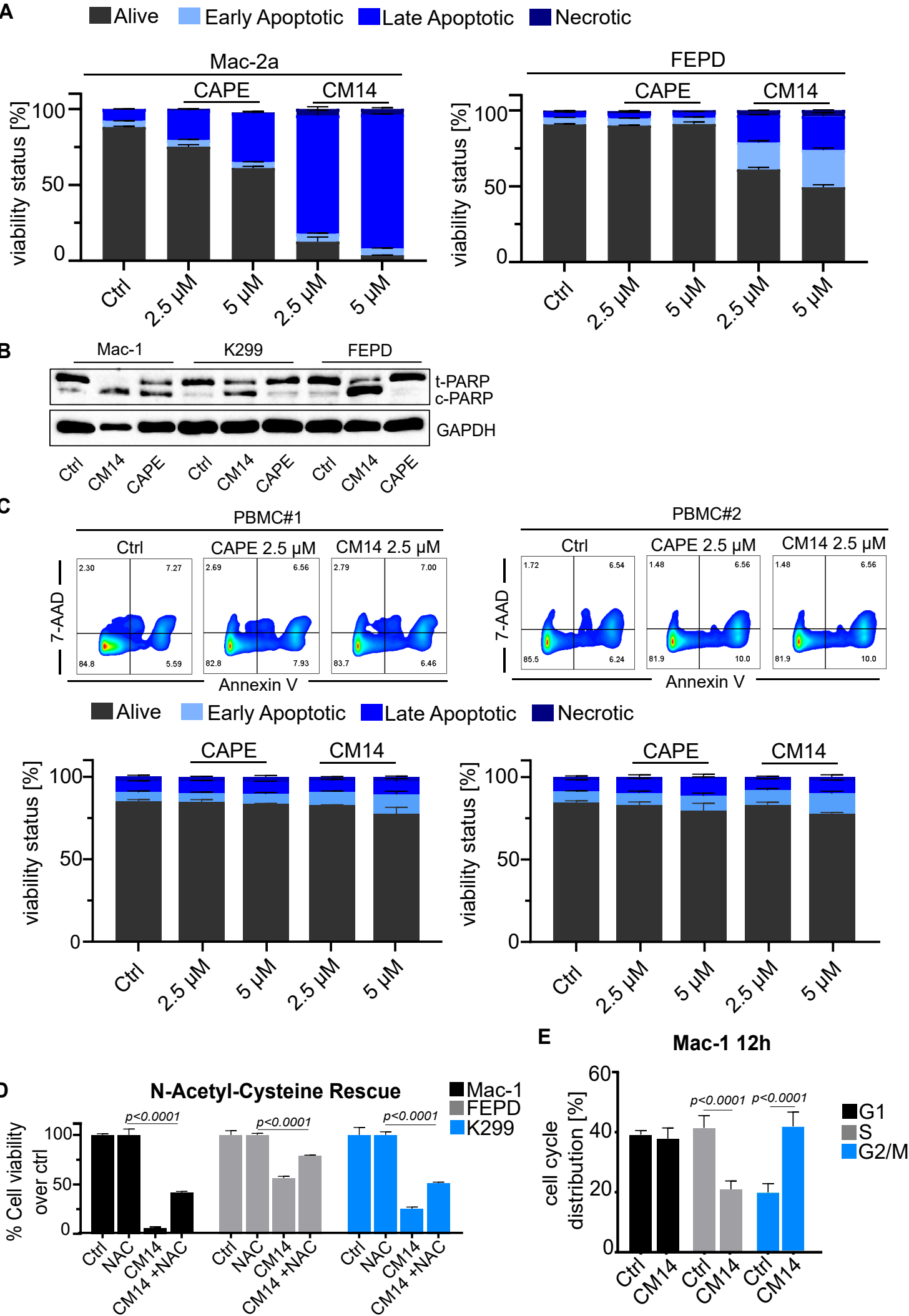

Supplementary Figure 2

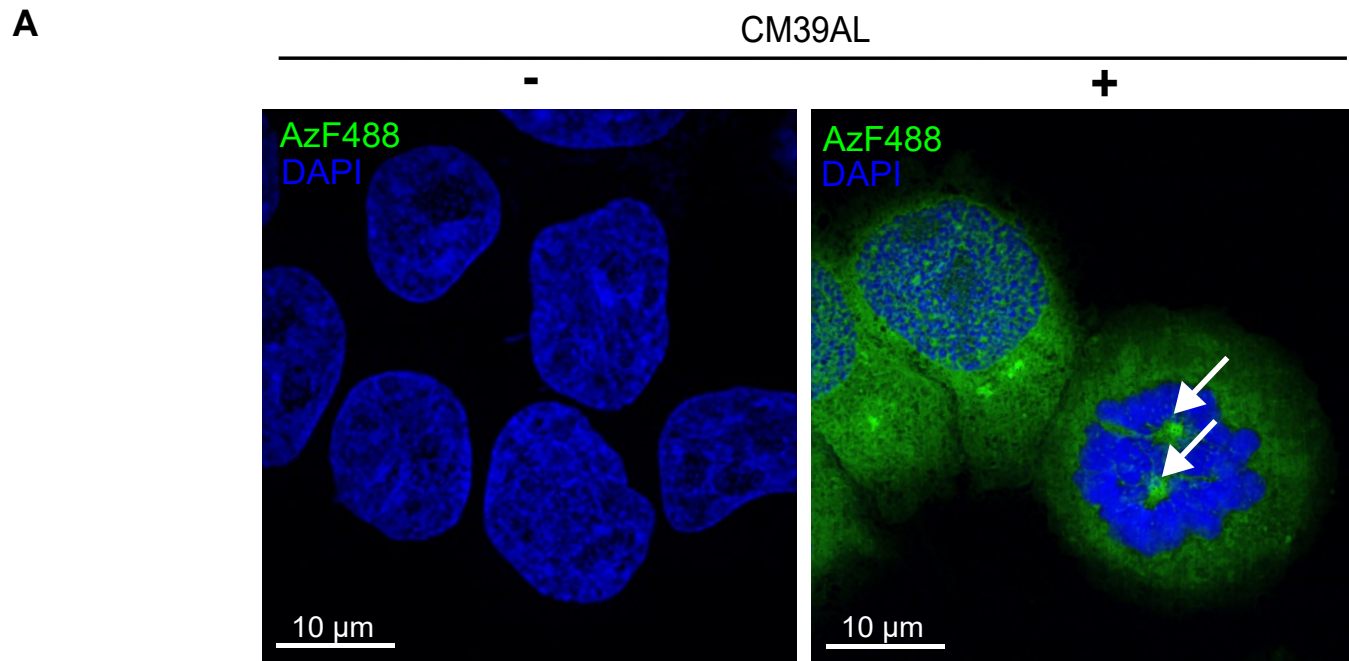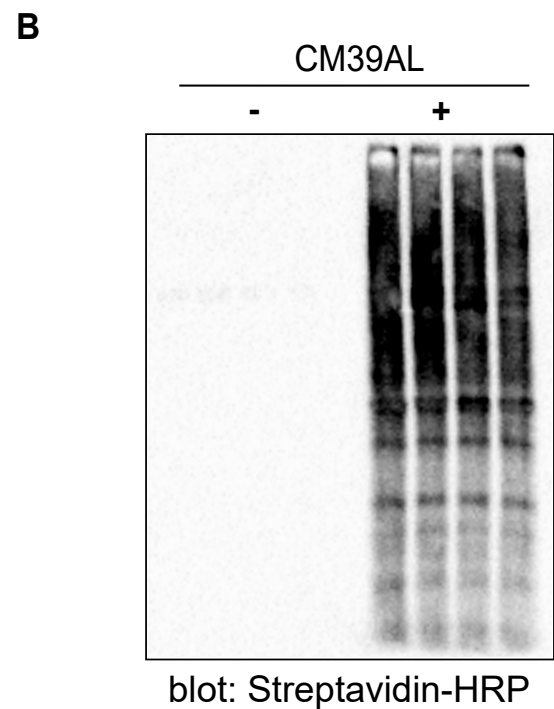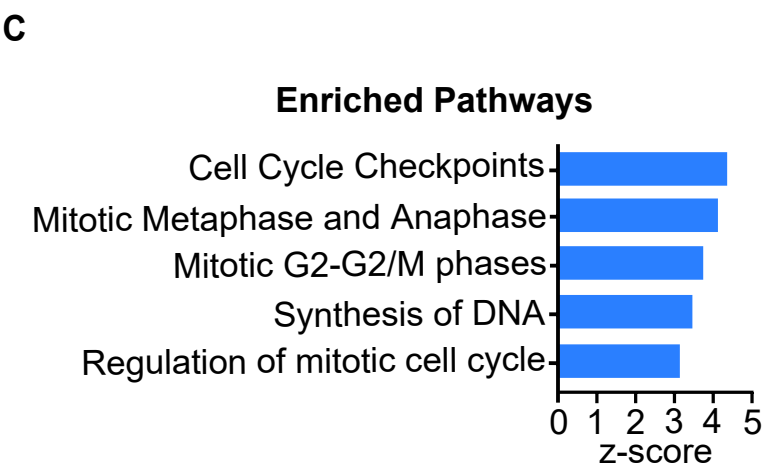

Supplementary Figure 3

A

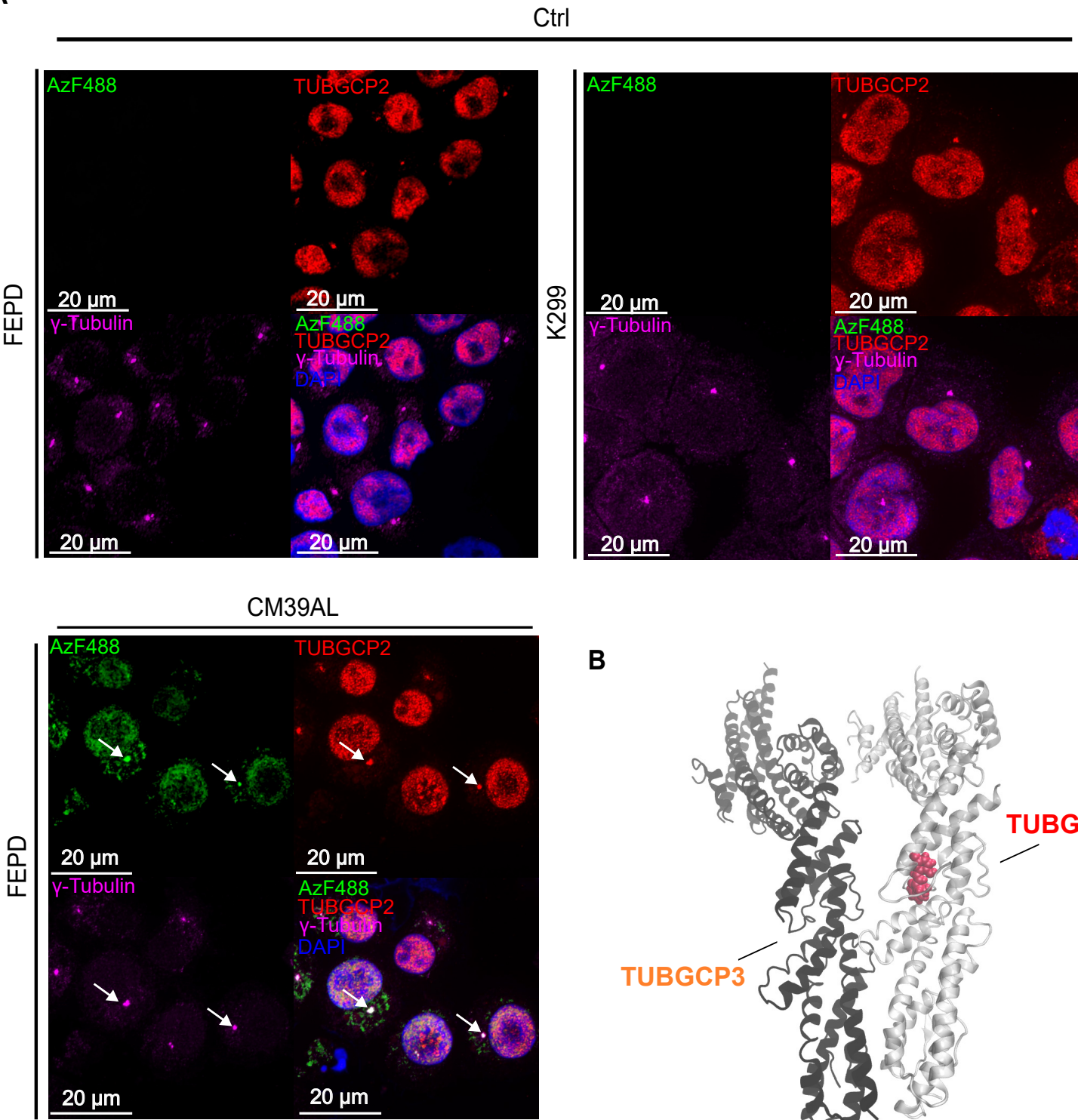

Supplement: Multimedia component 2 [file mmc2.pdf]
